# Exposure to Arsenic Alters the Microbiome of Larval Zebrafish

**DOI:** 10.3389/fmicb.2018.01323

**Published:** 2018-06-21

**Authors:** Dylan Dahan, Brooke A. Jude, Regina Lamendella, Felicia Keesing, Gabriel G. Perron

**Affiliations:** ^1^Department of Biology, Reem-Kayden Center for Science and Computation, Bard College, Annandale-On-Hudson, NY, United States; ^2^Department of Biology, Juniata College, Huntingdon, PA, United States

**Keywords:** microbiome, zebrafish, heavy metals, arsenic, integrons, DADA2, QIIME

## Abstract

Exposure to environmental toxins such as heavy metals can perturb the development and stability of microbial communities associated with human or animal hosts. Widespread arsenic contamination in rivers and riparian habitats therefore presents environmental and health concerns for populations living near sources of contamination. To investigate how arsenic affects host microbiomes, we sequenced and characterized the microbiomes of twenty larval zebrafish exposed to three concentrations of arsenic that are found in contaminated water—low (10 ppb), medium (50 ppb), and high (100 ppb) for 20 days. We found that even a small concentration of arsenic changed the overall microbial composition, structure and diversity of microbial communities, causing dysbiosis in developing larval zebrafish microbiota. In addition, we found that a high concentration of arsenic also increased the abundance of a class 1 integron, an integrase-dependent system facilitating the horizontal transfer of genes conferring resistance to heavy metals and antibiotics.

## Introduction

Arsenic, a metalloid element that can cause poisoning due the inhibition of acetyl-CoA and succinic dehydrogenase, is naturally present in many aquifers used for drinking water (Mukherjee and Bhattacharya, 2001; Bhadury, 2014). Since industrialization, however, runoff produced from mining, agricultural and industrial waste has vastly increased arsenic contamination in the environment (Sarkar and Bhattacharya, 2002). In humans, extended exposure to arsenic contamination can result in arsenicosis, which in turn can lead to skin cancer, keratosis, or important metabolic diseases such as diabetes (Guha Mazumder et al., 1998; Saha et al., 2003; Navas-Acien et al., 2008; Martinez et al., 2011). The effects of arsenic contamination have been extensively documented in Bangladesh, where up to 65 million people are exposed to drinking water with high concentrations of arsenic far exceeding the limit set by the World Health Organization (i.e., 50 ppb) or by local government (i.e., 10 ppb) (Smith et al., 2000; Mukherjee and Bhattacharya, 2001; World Health Organization, 2001). Still, more than seventy countries, including the United States, reported severe cases of arsenic poisoning in the past decades (Smedley and Kinniburgh, 2002; Saha et al., 2003), making arsenic contamination one of the principal environmental causes of cancer in humans (Hughes et al., 2011).

In addition to causing arsenicosis, arsenic in drinking water can also impact microbial communities associated with exposed hosts, also known as microbiota, or microbiomes (Palmer et al., 2007; de Theije et al., 2014; Narrowe, 2015; Claus et al., 2016). Previous studies showed that adult mice exposed to arsenic present important changes in their gastrointestinal intestinal (GI) tract microbiota (Lu et al., 2014; Dheer et al., 2015). Indeed, high concentrations of arsenic inhibit the growth of many bacterial species common in the GI (Summers et al., 1993; Lu et al., 2014). Arsenic can also alter hosts' microbiota by supporting the growth of arsenic-metabolizing bacteria such as *Sediminibacterium* and *Methylobacterium* (Oremland and Stolz, 2005; Mateos et al., 2006; Liao et al., 2011; Andres and Bertin, 2016). Such bacteria can metabolize arsenite or arsenate, often producing arginine metabolites toxic to the host (Dheer et al., 2015).

Microbial communities associated with hosts carry multiple functions that are essential to the hosts (Yan et al., 2016; Raymann and Moran, 2018). For this reason, microbiome aberrations, also called dysbiosis, are linked to disease occurrence in hosts ranging from diabetes and cardiovascular diseases to allergies and inflammatory diseases among other things (Turnbaugh et al., 2006; Foxx-Orenstein and Chey, 2012; Kong et al., 2012; Olszak et al., 2012; Qin et al., 2012). Because microbiomes are highly variable and are least stable during early developmental stages (As discussed by Palmer et al., 2007; Costello et al., 2012; Vallès et al., 2014), the effects of contaminants such as arsenic on dysbiosis are likely to be accentuated during early developmental stages (Neu and Rushing, 2011; Torrazza and Neu, 2011; Olszak et al., 2012). Investigating the effects of arsenic on microbial communities during the initial microbial establishment within hosts is thus crucial to predict outcomes on host health.

In this study, we investigate the potential effects of arsenic contamination on the developing microbiota of zebrafish (*Danio rerio*). In addition to being a robust model organism for the study of the microbiome and development in vertebrates (Veldman and Lin, 2008; Roeselers et al., 2011; Fritz et al., 2013; Kostic et al., 2013), zebrafish are native to arsenic-contaminated regions of Bangladesh and West Bengal (Spence et al., 2006). We exposed experimental populations of larval zebrafish to 10 ppb, 50 ppb, and 100 ppb sodium meta-arsenite for 20-days and used targeted 16S rRNA gene sequencing to characterize the fish microbiota after exposure. Additionally, we quantified the abundance of *int1*, a type 1 integron associated with the presence of antimicrobial resistance genes, in tested microbiomes. Our approach not only investigates how an environmental contaminant alters communities of microbes in developing hosts, but also how such contaminants may contribute to the spread of antibiotic resistance.

## Materials and methods

### Zebrafish husbandry and maintenance

Zebrafish (*Danio rerio*) were maintained in the laboratories of the Biology Department of Bard College in accordance with standard protocols for zebrafish husbandry (Lawrence, 2007). Zebrafish strain Et20, a transgenic animal expressing GFP in the neuromast supporting cells (Moon et al., 2011), were raised in a 14-h light:10-h dark cycle in standard recirculating rack water kept at 28.5°C with pH ranging from 7.0 to 7.4 unless specified. Prior to starting the experiment, embryos (0 days post-fertilization—or dpf) from a single mating were bleached twice in 0.5% sodium hypochlorite solution for 4 min. Embryos were then pooled into four groups of 30–60 animals and housed in petri dishes filled with 1x E3 media (5 mM NaCl, 0.17 mM KCl, 0.33 mM CaCl2, 0.33 mM MgSO4, 0.5 mg/L methylene blue) and one of the following treatments: 0, 10, 50, or 100 ppb sodium *meta*-arsenite (NaAsO_2_; A^3+^). At 48 h intervals, 80% of the media was exchanged for fresh media and sodium meta-arsenite solution. This was done to minimize cross-contamination due to dead cell tissue and to maintain a constant concentration of arsenite throughout the experiment.

Following larval hatching, between 72 and 96 h post-fertilization (hpf), populations were transferred to respective sterile glass dishes with growth conditions and experimental conditions similar to those described above. Again, media and sodium meta-arsenite concentrations were changed every 48 h. Animals were fed generously three times daily with micro powder food containing rotifers and *Paramecium*. At 20 dpf, zebrafish were sacrificed using sterile tricaine methane sulfonate solution (250 mg/l) according to established euthanasia techniques (NIH, 2009; Matthews and Varga, 2012). Fish were removed from tricaine following cessation of opercular movement (~10 min) and rinsed several times with nuclease free water to minimize the presence of free-living bacteria. All protocols were approved by the Bard College Institutional Animal Care and Use Committee (IACUC; most recent approval ID “Perron 2018”).

### DNA extraction

For each treatment, microbial DNA was extracted and purified from five individual fish larvae using a modified protocol for the DNeasy Blood and Tissue kit (QIAGEN, Germantown, MD) described in Hang et al. (2014). Briefly, sacrificed fish were treated with an enzymatic lysis buffer (20 mM Tris HCL, pH8.0; 2 mM sodium EDTA; 1.2% Triton X-100; and 20 mg/ml egg lysozyme) and incubated at 37°C for 60 min. To reduce protein contamination, we added 25 μl of Proteinase K solution (10 mg/ml; Qiagen, Germantown, MD) combined to 200 μl of Buffer AL (Germantown, MD), which we incubated at 56°C for 120 min. Following larval lysis and protein degradation, we used the DNeasy Blood & Tissue kit (Qiagen, Germantown, MD) to purify DNA from individual larval zebrafish. When necessary, gDNA concentrations were increased by evaporation using the SpeedVac System (ThermoFisher Scientific, Asheville, NC). Purified genomic DNA (gDNA) samples were stored in nuclease free H_2_O at −20°C.

### Primers and PCR amplification

Fish microbiota were characterized via targeted gene amplification of the 16S rRNA V4 region using Golay-barcoded primers 515F and 806R as described by Caporaso et al. (2012). Duplicate 25 μl PCR reactions (12.5 μl NEB Taq 2X Master Mix, 5.5 μl nuclease free PCR water, 1.0 μl of each forward and reverse primers (0.4 μM final concentration), and 3.0 μl genomic DNA template) were cycled as follows: denaturation at 94°C for 3 min; 35 cycles for 94°C/45 s, 50°C/60 s, and 72°C/90 s; and a final extension at 72°C for 10 min to complete amplification. Libraries were then were gel-purified, pooled at equimolar ratios, and sequenced on the MiSeq paired-end Illumina platform adapted for 150-bp paired-end reads (Wright Labs, Juniata College, Huntingdon, PA). The unfiltered forward reads are available on the Sequence Read Archive of the National Center for Biotechnology Information (SRA: SRP082969).

### Processing of 16S rRNA sequence data

Microbial communities were analyzed with two different microbiome pipelines to identify the presence and abundance of different microbial taxa based on the assembly of the 16S *rRNA* sequence reads. Before processing the 16S *rRNA* sequence data, phiX control reads were removed by mapping raw sequence reads against an indexed phiX genome provided by Illumina (San Diego, CA) using the Bowtie2 platform. Sequence reads were then processed, aligned, and categorized independently using either the DADA2 1.2 (Callahan et al., 2016: see scripts in Datasheet 1) or the QIIME 1.9.1 pipeline primarily using QIIME defaults parameters (Caporaso et al., 2010b).

The DADA2 microbiome pipeline (available at https://github.com/benjjneb/dada2) describes microbial communities using unique sequence variants present in the data, known as amplicon sequence variants (ASVs) (Callahan et al., 2016), rather than clustering groups of similar sequences into operational taxonomic units (OTUs) used by many other pipelines such as QIIME (Callahan et al., 2016). In brief, sequence reads were first filtered using DADA2's recommended parameters (i.e., an expected error threshold of 2 combined with the trimming of 10 nucleotides from the start and end of each read). Filtered reads were then de-replicated and de-noised using DADA2 default parameters. De-replication combines identical reads into unique sequences and constructs consensus quality profiles for each combined lot of sequences; the consensus quality profiles then inform the de-noising algorithm which infers error rates from samples and removes identified sequencing errors from the samples. After building the ASV table and removing chimeras, taxonomy was assigned using the Ribosomal Database Project (RDP) classifier (v2.2) (Wang et al., 2007) natively implemented in DADA2 and trained against the Greengenes reference database (13.8) (Werner et al., 2011) (McDonald et al., 2011). A phylogenetic tree was built using FastTree (v2.1.3) (Price et al., 2010) from a multiple sequence alignment made with the PyNAST alignment tool (Caporaso et al., 2010a), against the Greengenes Core reference alignment (DeSantis et al., 2006). Lastly, we pruned sequences positively mapping to zebrafish mitochondrial gDNA (GenBank: KT624622.1) using a BLAST alignment.

For comparison purposes, we also processed the raw reads using QIIME 1.9.1 (available at https://github.com/biocore/qiime) as described elsewhere (Caporaso et al., 2010b; Edgar et al., 2011). We first quality-filtered reads using default parameters, removing reads with Phred quality scores below 30, and removed chimeric sequences using UCHIME (Edgar, 2010; Edgar et al., 2011). The OTU table was built using the uclust method (Edgar, 2010) at a ≥97% identity threshold and the open reference picking method using the PyNAST sequence aligner against the Greengenes Core reference alignment (DeSantis et al., 2006; Caporaso et al., 2010a) for the closed reference alignment. Again, we pruned sequences identified as zebrafish mitochondria DNA.

### Data visualization and statistical analyses of 16S rRNA sequence data

Patterns of diversity within the ASV and OTU tables were analyzed using a custom bioinformatic pipeline implemented in R 3.2.3 (http://www.r-project.org) and described in Datasheet 2 (mapping file linking sample names and arsenic concentration is also included in Datasheet 3). First, we visualized phylum and family level community composition using phyloseq (v1.14.0) (available at https://joey711.github.io/phyloseq/) (McMurdie and Holmes, 2013) implemented in ggplot2 (v.2.0.0) (Wickham, 2009), with visualization optimized by filtering taxa at cutoffs of 0.1 and 0.3% for phylum and family respectively. Using the RNA-Seq DESeq2 differential abundance comparison (Love et al., 2014) adapted for use with microbial count data (McMurdie and Holmes, 2014), we investigated changes in community composition at the taxa level. This method stabilizes sample variance using an implementation for count data that is built on a negative binomial distribution, therefore allowing for the use of a non-normalized and not rarefied ASV table. This is preferred to rarefying since subsampling inflates variance, resulting in a loss of power and often producing false positive results (McMurdie and Holmes, 2014).

We then estimated alpha diversity metrics using phyloseq's estimate_richness function on rarefied ASV tables. More specifically, we estimated observes species *S*, i.e., observed ASVs or OTUs, Simpson's metric, Shannon diversity measurements *H*′, as well as Pielou's evenness index. Pielou's evenness metric was computed with the following equation (Pielou, 1966):

$$\begin{array}{l} J^{\prime }=\frac{H^{\prime }}{{H^{\prime }}_{\max}} \end{array}$$

where ${H\prime }_{max}$ is the maximum value of *H*′ if every species were equally abundant:

$$\begin{array}{l} {H^{\prime }}_{\max}=\overset{S}{\underset{i=1}{\sum }}\frac{1}{S}\ln\frac{1}{S}=lnS \end{array}$$

We compared statistical models using Akaike's Information Criterion (AIC) in R's base stats package.

### Beta-diversity

Phyloseq was used to perform ordinations, where Principle Coordinate Analysis (PCoA) was employed on unweighted UniFrac distance scores (Lozupone and Knight, 2005). To confirm multivariate homogeneity of variances first among treatments and second within treatments, the vegan (v2.3.2) package implementation of PERMDISP2 via the betadisper method was used (Oksanen et al., 2015). To test the effect of arsenic as a continuous variable on group differences according to UniFrac scores, a permutational analysis of variance (PERMANOVA) using the adonis function as part of the vegan package was used (Oksanen et al., 2015). An analysis of similarity (ANOSIM) was conducted to compare the variation in UniFrac distances between a grouped arsenic distance matrix and control group distance matrix as again implemented in the vegan package.

### Comparing ASVs and OTUs

We first tested the null hypothesis that the observed richness of OTUs and ASVs after processing remained the same by conducting a Wilcoxon test between sample counts. Differences between log-transformed observed richness of OTUs and ASVs from the same treatments were compared using an analysis of variance (ANOVA) with treatment condition as a factor, followed by Tukey's honest significant difference (HSD) tests. Differentially abundant ASVs and OTUs were identified using the DESeq2 package (Love et al., 2014), adapted for use with microbial community count data (McMurdie and Holmes, 2014), with a local fit type. When performing Procrustes rotation between matching points from ASV and OTU UniFrac distance matrices, the ade4 package (v.1.7.3) was used as implemented in R (Thioulouse and Dray, 2007). A PROcrustean randomization TEST (PROTEST) was conducted to test the significance of Procrustes matrix concordance again using the package ade4 package (Jackson, 1995). Data was visualized using ggplot2 (v.2.0.0) (Wickham, 2009). Finally, we used biom (v0.3.12) to handle BIOM formatted files, dada2 (v0.10.6) for read processing (Callahan et al., 2016), optparse (v1.3.2) to parse command line options, stats (v3.2.3) to conduct R's internal statistics, and data.table (v1.9.6) to handle data frames.

### qPCR of 16S rRNA and *Int1* genes

We quantified 16S rRNA and *Int1* gene copy numbers using quantitative real-time PCR as described in Gaze et al. (2011). For each sample, triplicate PCR reactions using the PowerUp SYBR Green Master Mix (Applied Biosystems, Foster City, CA) were cycled using the Bio-Rad CFX96 Real-Time PCR Detection System (Bio-Rad Laboratories, Hercules, CA) with the following cycling conditions: Hot start at 50°C for 2 min; denaturation at 95°C for 10 min; followed by 40 cycles of 95°C/20 s and 60°C/60 s; with an added dissociation step. An internal standard curve constructed from a serial dilution of *Escherichia coli* SK4903, an engineered *E. coli* strains harboring seven 16S rRNA copies and six *int1* copies, was processed with each qPCR run. The relative abundance of *int1* genes was normalized to 16S rRNA copies of each sample before being analyzed via linear modeling with *int1* relative abundance as response variable and arsenic concentrations as explanatory variable.

## Results

### Processing 16S V4 reads using DADA2

**Figure 1:**
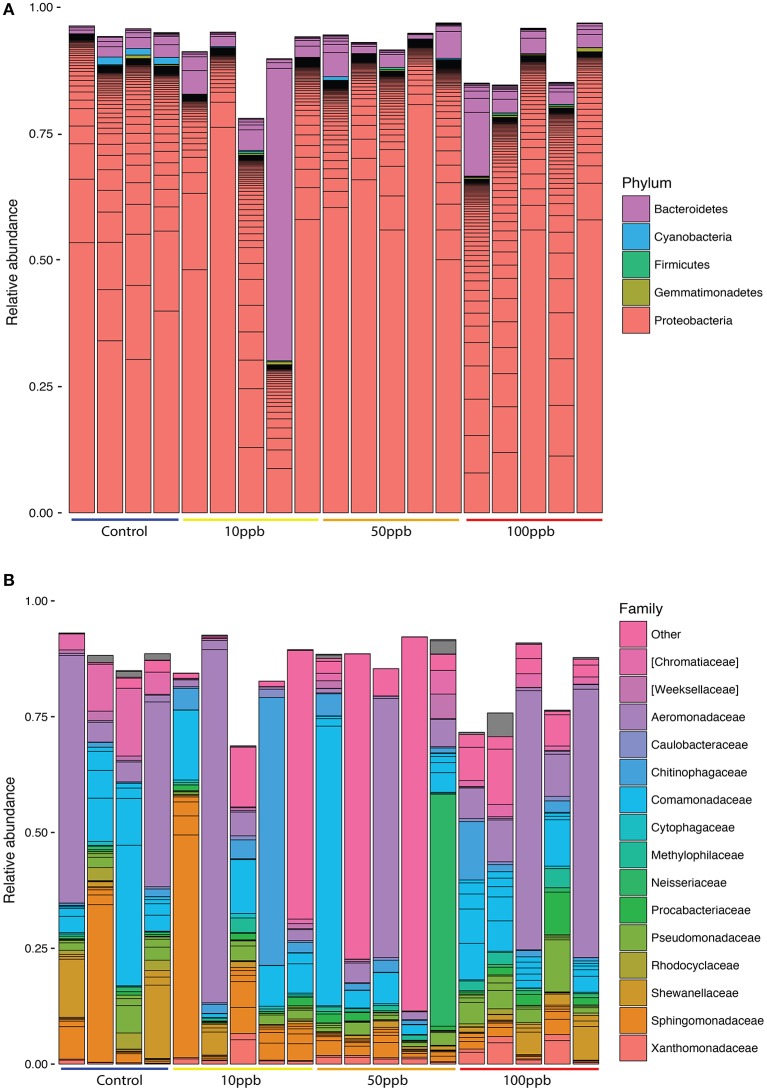
Relative abundance of taxa across treatments. **(A)** Phylum level classification, filtered to remove taxa below 0.001 abundance. **(B)** Family level classification, filtered to remove taxa below 0.003 abundance. Lines within same-colored bars are of different ASVs of the same classification. Category “Other” represents ASVs that were not classified to the family level. Grey areas are unclassified ASVs. Control *n* = 4; 10 ppb *n* = 5; 50 ppb *n* = 5; 100 ppb *n* = 5.

We characterized the microbiomes of twenty individual zebrafish larvae, five individuals per treatment (0, 10, 50, or 100 ppb of arsenic) by sequencing the V4 region of the 16S rRNA. We obtained a total of 10,108,755 forward reads with an average read length of 151 base pairs, totaling ~1.5 G bases. The median sequencing depth per sample was 497,470 reads. Reverse reads were of poor quality due to a sequencing error and were therefore removed from subsequent analyses along with one control sample. After filtering, denoising, and removing chimeras, we retained 8,678,233 (85.86% of initial) reads. With these reads, we assigned taxa (see Datasheet 4 for unfiltered representative ASVs) and plotted relative abundance of taxa across samples (Figure 1).

We identified 120 ASVs as part of the core microbiome among the four zebrafish sampled from the control population (Table 1; Table S1). Interestingly, we found that the core microbiome described in this study, even though extracted from the whole larvae, was compositionally similar to that of healthy zebrafish gut microbiomes either living in wild or laboratory conditions (Roeselers et al., 2011). This is most likely due to the fact that, as in animals (Sender et al., 2016), fish microbiota are most dense in the gastrointestinal tract.

**Table 1:** Relative abundance (RA) for core phyla abundances ± s.e.

**Phyla**	**Mean RA (%)**	**s.e. (%)**
*Proteobacteria*	94.10	2.20
*Bacteroidetes*	4.17	0.29
*Cyanobacteria*	1.06	0.34
*Gemmatimonadetes*	0.29	0.12
*Firmicutes*	0.12	0.02

### Arsenic exposure alters microbial community composition

**Figure 2:**
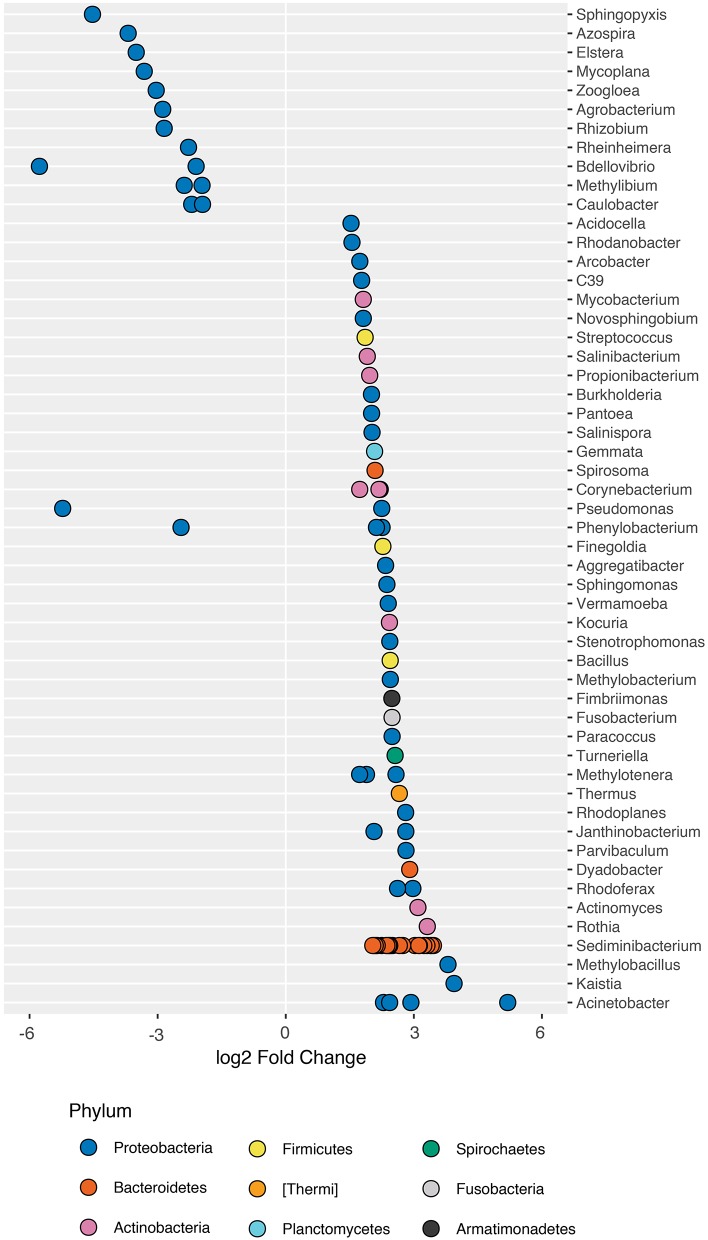
ASVs that significantly differ in abundance in the presence of arsenic. 186 ASVs were identified as significantly different in abundance in the presence of arsenic (DESeq2; adj-*P* < 0.05; ST1 for complete list of differentially abundance ASVs). 43 ASVs that increased in abundance and 38 that decreased were not assigned taxonomy to the genus level and were removed for plotting purposes. Genus-level classification is provided where available.

When comparing the composition of healthy fish microbiomes to that of fish exposed to arsenic, we found that a total of 78 ASVs significantly differed in abundance (DESeq2; adj-*P* ≤ 0.01; Figure 2; Table S2). ASVs that increased in abundance included genera such as *Acinetobacter* (base mean: 125)*, Sediminibacterium* (base mean: 201) and *Janthinobacterium* (base mean: 315), whereas genera that decreased in abundance included *Bdellovibrio* (base mean: 110), and *Pseudomonas* (base mean: 131) among others. Interestingly a *Legionellales* ASV (ASV3) dominated the increase in the arsenic treatment (base mean: 8,464), but could not be identified at the genus level.

When considering a less conservative alpha-level (α = 0.05) for the detection of trends in taxa that changed in abundance in response to arsenic treatment, we found that 186 ASVs differed in abundance (DESeq2; adj-*P*s < 0.05; Table S3). We found that ASVs within individual genera at times responded differently to arsenic exposure, indicative of phenotypic heterogeneity within the genus. For example, within *Pseudomonas*, variant ASV32 (base mean: 1,200) increased by a ~2.3x while ASV152 (base mean: 131.1) decreased in abundance by a ~5.2x under the effect of arsenic when compared to its abundance in control populations. Interestingly, certain species within the genus such as *P. aeruginosa* are associated with antimicrobial resistance (Perron et al., 2007).

### Arsenic exposure alters alpha diversity in fish microbiome

**Figure 3:**
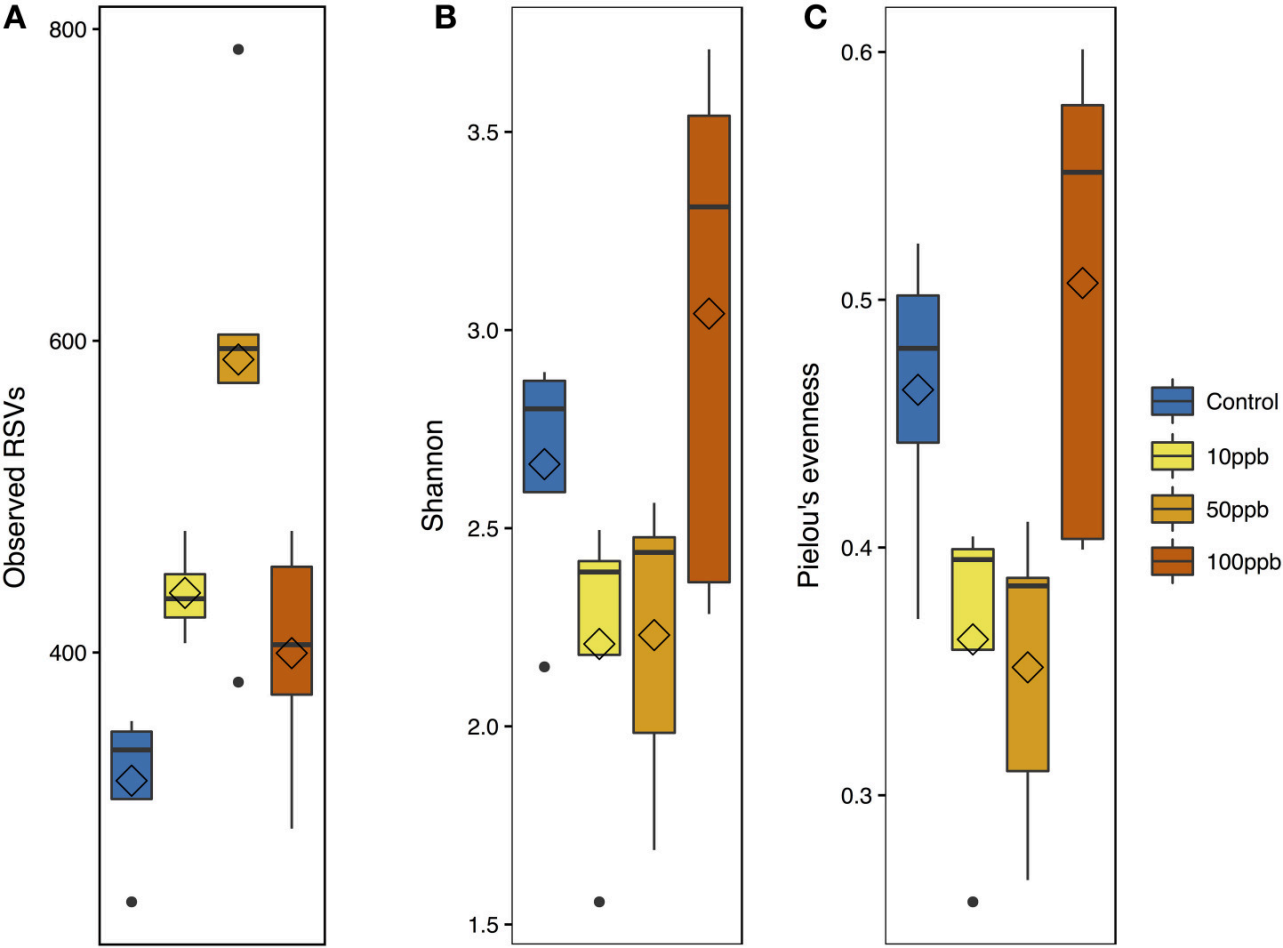
Alpha diversity metrics with arsenic exposure. **(A)** Observed ASV measurements [*F*_(2, 15)_ = 9.83; adj-*P* < 0.01]. **(B)** Shannon diversity measurements [*F*_(2, 15)_ = 4.32; adj-*P* = 0.03]. **(C)** Pielou's evenness measurements [*F*_(2, 15)_ = 6.04; adj-*P* = 0.01]. All metrics are plotted against arsenic concentration gradient; with mean (diamond) and median (line), and hinges as first and third quartiles (25th and 75th percentiles).

We compared the overall taxonomic diversity across treatments using different metrics of alpha diversity that incorporate species richness and evenness (Table 2). We estimated observed richness (i.e., number of ASVs), as well as the Shannon index and Pielou's evenness metric from the ASV table rarefied to the lowest sampling depth (119,609 ASVs). Looking at richness, we found a quadratic relationship between the number of observed ASVs and arsenic concentrations (*F*_(2, 15)_ = 9.83; *P* < 0.01; Figure 3A). More specifically, we found that the highest number of ASVs was observed at intermediate arsenic concentrations: the number of ASVs increasing by 1.9-fold from ~319.3 ASVs at 0 ppb to ~580.0 ASVs at 50 ppb before decreasing when exposed to ~393.2 ASVs 100 ppb. When considering both Shannon diversity and Pielou's evenness, however, we found that arsenic generally decreased diversity at 10 and 50 ppb and increased diversity at 100 ppb. Specifically, we found that, relative to the control, Shannon diversity decreased by 1.2x from 0 to 50 ppb, but increased by 1.1x when compared to the control at 100 ppb (*F*_(2, 15)_ = 4.32, *P* = 0.03; Figure 3B). Similarly, Pielou's evenness decreased by 1.3x from 0 to 50 ppb and increased by 1.1x at 100 ppb (*F*_(2, 15)_ = 6.04, *P* = 0.01; Figure 3C; Table S6). Because Shannon diversity index is calculated using both observed ASVs and their relative abundance, the lack of congruence between the latter and observed ASVs suggest that changes in ecosystem diversity are likely due to fluctuations in relative abundance of different ASVs following arsenic exposure. In other words, even though the number of ASVs observed in arsenic treatment is higher than in control population, the microbiome exposed to arsenic is likely dominated by relatively few taxa.

**Table 2:** Means for alpha diversity metrics of zebrafish microbial communities ± s.e.

**Treatment**	**Observed**	**Shannon**	**Pielou**
Control	319.25 ± 32.9	2.66 ± 0.30	0.46 ± 0.043
10 ppb	431.75 ± 15.1	2.19 ± 0.23	0.36 ± 0.037
50 ppb	580.00 ± 66.8	2.23 ± 0.17	0.35 ± 0.028
100 ppb	393.20 ± 26.0	3.03 ± 0.17	0.51 ± 0.033

### Arsenic exposure alters community composition in fish microbiome

**Figure 4:**
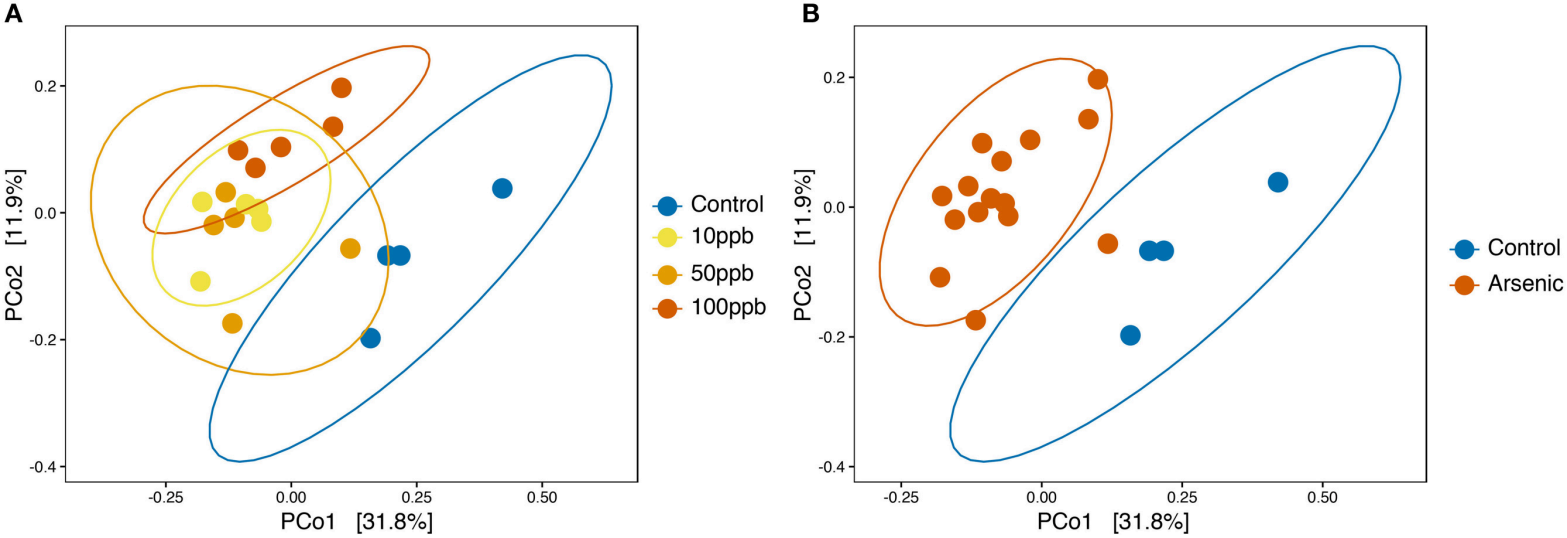
Principle coordinates analysis (PCoA) based on unweighted UniFrac scores of zebrafish microbiota. **(A)** PCoA on unweighted UniFrac scores with all treatments labeled. Significant dissimilarity between samples when arsenic was treated as a continuous variable (ADONIS; adj-*P* = 0.036; *R*^2^ = 0.124; perm = 999). **(B)** PCoA on unweighted UniFrac scores with arsenic pooled group and control. Significant dissimilarity between the pooled arsenic group and control group (ANOSIM; adj-*P* < 0.01, *R* = 0.786, perm = 999). Ellipses are drawn at 0.95 C.I.

We then tested whether arsenic exposure resulted in changes in overall microbial community composition, or beta-diversity. Using principle coordinate analysis (PCoA) on unweighted UniFrac scores, we found that the two highest-ranked dimensions, PCo1 and PCo2, explained 31.8 and 11.9% of variance respectively (Figure 4A) and that arsenic exposure itself explained 78.4% of the variance in bacterial composition, resulting in clustering of arsenic treated microbiomes compared to control microbiomes (ANOSIM; *R* = 0.784; adj-*P* < 0.01; perm = 999; ANOSIM; Figure 4A; Table S4). When treating arsenic concentration as a continuous variable, we found that UniFrac scores tended to diverge away from control microbiomes as concentration increased (ADONIS; *R*^2^ = 0.12; perm = 999; adj-*P* = 0.034; Figure 4B; Table S5). We found no significant differences in sample distances to their treatment centroids, suggesting homogeneity of variance (adj-*P*s > 0.05; ANOVA; Tables S7,S8). Not surprisingly, many of the genera found to increase in abundance in the presence of arsenic (Figure 2), e.g., *Sediminibacterium, Methylotenera, Sphingomonas*, and *Dyella*, also clustered with arsenic samples.

### Comparing OTUs and ASVs

We replicated the above analyses using the OTU table produced by QIIME defaults, utilizing a method based on the uclust algorithm which cluster reads into OTUs based on ≥97% similarity (see Datasheet 5 for unfiltered representative OTUs). On average, we observed five times more OTUs per sample (mean: 3,502 ± s.e. 125.9) than ASVs (mean: 598.0 ± s.e. 46.16.) (Wilcoxon test; W = 361; *P* < 0.01; Figure S1). We also observed higher variance in the number of OTUs identified between samples (Fligner-Killeen test; χ^2^ = 6.58; *P* = 0.0126). This difference was partly explained by the fact that 40.80% of OTUs were doubletons while only 5.55% of ASVs were doubletons, most likely due to DADA2 correcting probable sequencing errors in sequences at low abundance (Callahan et al., 2016).

When looking at species composition, we observed a similar pattern among OTUs distribution: 148 OTUs significantly differed in abundance in the presence of arsenic (DESeq2; adj-*P*s ≤ 0.01; Figure S2; Table S10) with an important overlap between genera identified via OTUs and ASVs. However, we did not detect significant changes in alpha-diversity between arsenic and control treatments when using the rarefied OTU table (using a depth of 96,879 reads per sample; Figure S3). The differences between the two analyses are most likely due to fewer reads post-quality filtering in the QIIME pipeline (median: 245,577) compared to the DADA2 methods (median: 497,470) and increased spurious resolution when using OTUs rather than ASVs. Despite the lack of difference at the alpha-diversity level, we found that the presence of arsenic explained a high degree of the variance (54.7%) in overall bacterial composition when comparing arsenic and control sample measured as unweighted UniFrac scores, in accordance with DADA2 (ANOSIM; *R* = 0.547; *P* < 0.01; perm = 999; Figure S4). Finally, a Procrustes analysis revealed a high level of similarity between the two analyses (PROTEST; *P* < 0.01; Observation = 0.758; perm = 999), confirming that despite differences between OTU and ASV results, the two methods reveal similar taxonomic composition throughout the experiment.

### High concentrations of arsenic increase *Int1* abundance in fish microbiome

**Figure 5:**
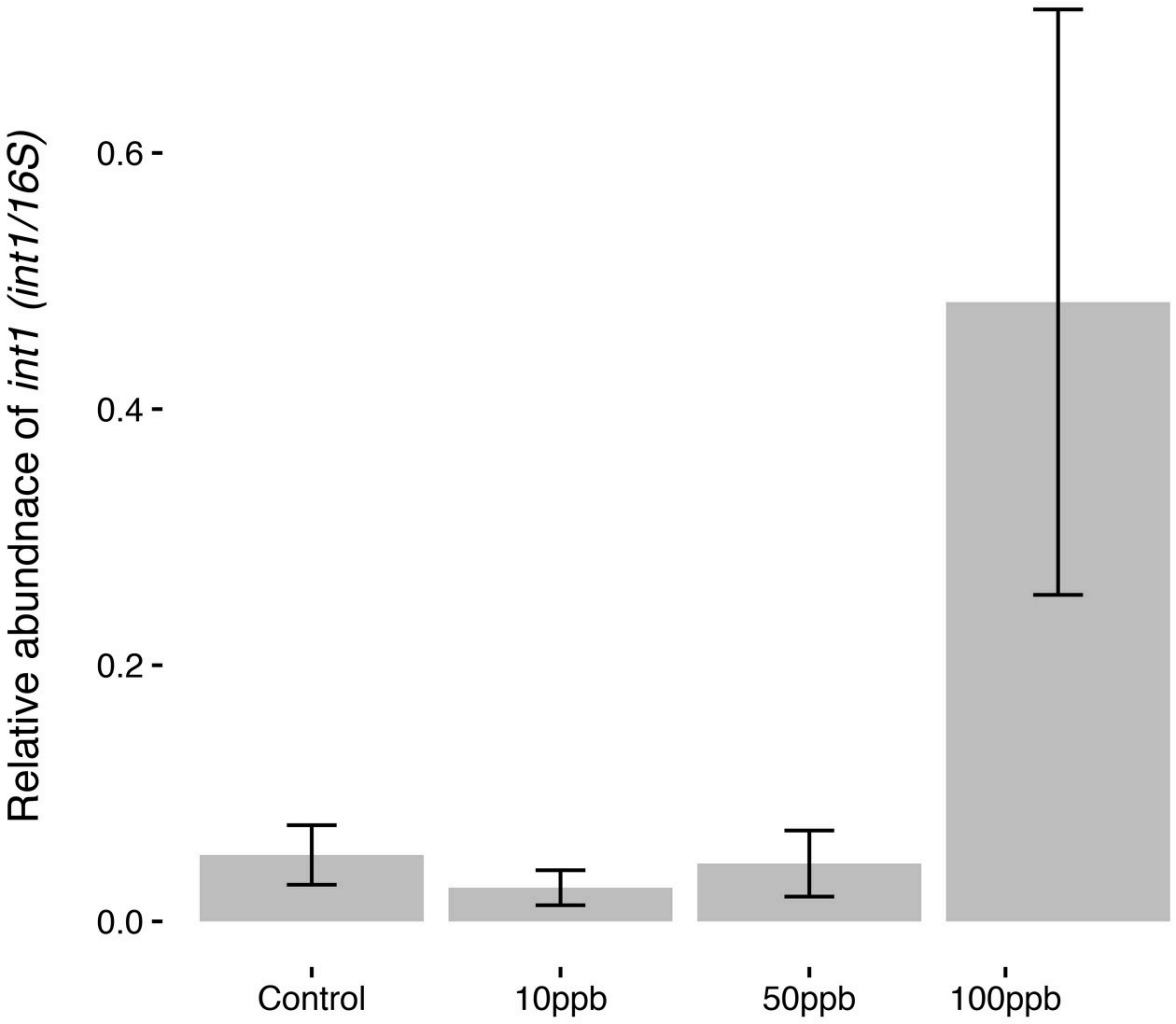
*Int1* abundance in response to arsenic concentration. Relative abundance of *int1* is taken as the proportion of *int1* copies per 16S rRNA copies, where copy numbers are calculated using standard curves and qPCR. Error bars are ± standard error. Quadratic model suggests highest arsenic concentration (100 ppb) significantly increased *int1* abundance (*F*_(2,14)_ = 13.27; *P* < 0.01).

Finally, following the observation that certain taxa increasing in abundance in the presence of arsenic were previously associated with antimicrobial resistance, we measured the relative abundance of *int1* to investigate the possible link between arsenic exposure and the spread of integron-like structures in microbial communities. We found that that arsenic concentration had a significant effect on the relative abundance of *int1*: exposure to high arsenic concentrations correlated with an increase in *int1* copies (*F*_(2, 14)_ = 13.27; *P* < 0.01; Figure 5). The effect of arsenic was especially strong at the highest concentration where *int1* relative copy number was 9 times higher than in control populations. Using a dissimilarity matrix comparing principle coordinate analysis (PCoA) to *int1* abundance, we found that *int1* did not significantly correlate with UniFrac clustering between the different samples (ADONIS; *R* = 0.056; *P* = 0.56; perm = 999; Figure S5), indicating that increases in *int1* relative copy number was not likely associated with one or a few specific taxa.

## Discussion

Mounting concern over arsenic contamination in drinking water has led to a better understanding of both arsenic's negative outcomes on human health (Smith et al., 2000) and its influence on microbial communities in the environment (Cai et al., 2009; Sarkar et al., 2012; Escudero et al., 2013; Bhadury, 2014; Farias et al., 2015; Paul et al., 2015; Sultana et al., 2017). In this study, we investigated whether environmental concentrations of arsenic can perturb microbiota in developing zebrafish larvae. We found that exposure to arsenic had a destabilizing effect on the zebrafish microbiome, leading to dysbiosis both in terms of microbial diversity and in terms of microbial composition between arsenic treatments. We also found that exposure to a high concentration of arsenic increased the frequency of *int1*, a genetic mechanism responsible for the horizontal transfer of resistance genes. To the best of our knowledge, this is the first study examining how arsenic affects developing zebrafish microbiomes.

Overall, we found that arsenic created distinguishable microbial communities comparable across the different level of arsenic exposure. We found that even a concentration as low as 10 ppb, considered safe by the World Health Organization, altered the larval microbiome. Interestingly, 12 out of the 15 most abundant families present in the core microbiome of healthy larvae significantly changed in abundance in the presence of arsenic. We also observed a reduction in taxa evenness at all levels of arsenic exposure and an increase in the total number of observed taxa at 10 and 50 ppb. While a decrease in evenness was associated with adverse health effects in premature infants with necrotizing enterocolitis (Chang et al., 2008; McMurtry, 2015), increases in taxa richness at low concentrations of arsenic were also observed in contaminated aquifers (Li et al., 2015). These observations agree with the perturbation theory of the subsidy-stress gradient, stating that low levels of stress can potentially enhance ecosystem productivity via the input of energy (Odum et al., 1979).

Many of the taxa that increased in abundance in the presence of arsenic were also observed in arsenic-contaminated environments (Oremland and Stolz, 2005; Liao et al., 2011; Costa et al., 2015). For example, *Sediminibacterium* and *Acinetobacter*, found in streams contaminated with arsenic (Costa et al., 2015; Li et al., 2015), were also found to harbor arsenic resistance genes (e.g., *ars* operon and ACR3 protein). Interestingly, while the 26 ASVs belonging to *Sediminibacteirum* significantly increased in abundance, this was not the case in other genera such as *Phenylobacterium*, where two ASVs increased in abundance and one decreased. These results suggest that the response of different taxa to environmental perturbations is not predictable from the phylogenetic relatedness of microbes alone. Not only bacterial lineages can vary a great deal within a genus or even within a species, but the spread of arsenic resistance genes such as *ars* can also be influenced by the horizontal transfer of genetic material (Sarkar et al., 2012).

Indeed, our results show that exposure to arsenic can increase the abundance of *int1* genes in microbial communities, an important genetic factor responsible for the transmission of gene cassettes conferring resistance to heavy metals and different antibiotics (Naas et al., 2001; Fluit and Schmitz, 2004; Gaze et al., 2011; Koenig et al., 2011). Interestingly, while our data suggest that high concentrations of arsenic (i.e., 100 ppb) strongly increased *int1* abundance, Guo et al. (2014) noted that type 1 integrons did not increase in the gut microbiota of mice exposed to 3,000 ppb of arsenic trioxide. Our study thus implies that arsenic contamination possibly exerts different selective pressures in different hosts or could be stronger in aquatic environments. Taken together, our results suggest that arsenic exposure not only select directly for taxa harboring resistance mechanisms, but also for strains of bacteria with high recombinogenic activity, possibly contributing to the presence of antibiotic resistance reservoirs in aquifers and animals (Baker-Austin et al., 2006; Gullberg et al., 2014; Chen et al., 2015).

The combination of two computational methods to analyze the effect of arsenic on microbial communities offered the opportunity to compare microbial diversity at different levels. While uclust's OTU picking method clusters similar sequences based on sequence differences at a fixed 97% similarity threshold (Caporaso et al., 2010b; Edgar, 2010), DADA2 integrates sequence variation, quality scores and an error model in order to infer exact sample sequences from amplicon reads (Callahan et al., 2016). The coarse-grain method favored by OTU-picking defined by uclust can be a good indicator of broad-scale patterns in microbial communities, especially when prior functional clades are known (Koeppel and Wu, 2013).

Crucially, the overall effect of arsenic on microbial composition remained a significant factor when considering both OTUs and ASVs, highlighting the fact that arsenic plays an important role in dysbiosis both at the fine and coarse-grain levels. Still, our results also suggest that sequence variants-based analyses are more powerful for detecting changes associated with final-scale microbial diversity. While OTU-based analyses revealed more taxa overall, such analyses failed to detect changes in abundance associated with important taxa. This result likely reflects the fact that uclust clusters polyphyletic reads into OTUs and misses variation at lower phylogenetic levels (Koeppel and Wu, 2013). The higher number of taxa observed in OTUs is likely due to the high false-positive rates associated with Illumina sequencing (Callahan et al., 2016), as supported by the fact that 40.8% of OTUs were doubletons and only 5.55% of ASVs were doubletons. In addition to providing a more accurate description of microbial communities, sequence variant-based analyses are likely to provide the opportunity to distinguish between similar sequences that can be crucial to revealing the difference between harmless and virulent strains as more information is gathered (McElroy et al., 2013).

Finally, even though we replaced media every 48 h to reduce cross-contamination between individual larvae, it is possible that the environment in which the fish were raised confounded the effect observed in this study. The fact that fish from separate treatments were raised in the same dishes could explain part of the variance observed among the different microbiomes, a phenomenon known as batch effect (Hurlbert, 1984). Still, microbiomes exhibited high inter-samples variability as expected with most 16S amplicon studies (Hiergeist et al., 2016), suggesting that the level of variability in our study was not limited by specific batches. Furthermore, we observed a strong overall effect of arsenic on the investigated microbiomes as well as linear trends in both *int1* relative abundance and diversity measurements in relation to arsenic concentration, indicating that the data structure follows a gradient along arsenic concentration rather than being randomly assigned to batches. Taken together, these results suggest arsenic most likely exerted a predictable effect on the microbial communities.

In conclusion, our study demonstrates that concentrations of arsenic, even below maximum concentration limits mandated by local authorities, can alter the developing microbiota of zebrafish and increase the relative abundance of integron 1 genetic elements. The possible risk of pathogenicity associated with highly recombinogenic strains remains to be investigated in this system (Stecher et al., 2013). Future studies should also integrate multi-omics approaches with physiological studies to determine how arsenic exposure alters the microbiome functions in developing hosts with possible health consequences, as well as the consequences of these changes for the health of humans and other animals.

## Author contributions

DD, FK, and GP designed the experiment. DD, GP, and BJ designed and conducted experimental procedures, including zebrafish arsenic exposures, gDNA extractions, MiSeq library preparation, and qPCR. RL led the sequencing project as well as provided bar-coded reverse primers. DD and GP conducted bioinformatic and statistical analyses. DD and GP were the major contributors in writing the manuscript. All authors read and approved the final manuscript.

### Conflict of interest statement

The authors declare that the research was conducted in the absence of any commercial or financial relationships that could be construed as a potential conflict of interest.

## Supplementary material

Figure S1 Compared observed richness of ASVs and OTUs.

Figure S2 OTUs differentially abundant in the presence of arsenic (*P* ≤ 0.01).

Figure S3 Alpha diversity metrics, measured with OTUs, with arsenic exposure.

Figure S4 Principle coordinates analysis (PCoA) based on unweighted UniFrac scores of zebrafish microbiota, using OTU table.

Figure S5 PCoA based on unweighted UniFrac scores of ASVs from zebrafish microbiota amplified for *int1*.

Datasheet 1 R markdown file with scripts outlining DADA2 forward read processing.

Datasheet 2 R markdown file with scripts outlining community analyses.

Datasheet 3 Mapping file with sample data.

Datasheet 4 Fasta file with representative ASVs.

Datasheet 5 Fasta file with representative OTUs.

Table S1 Core microbiome in control population.

Table S2 ASVs differentially abundant in the presence of arsenic (*P* ≤ 0.01).

Table S3 ASVs differentially abundant in the presence of arsenic *(P* ≤ 0.05).

Tables S4, S5 ANOVA tables for PCoA comparisons.

Table S6 OTUs differentially abundant in the presence of arsenic.

Table S7 Genera counts of differentially abundant ASVs and OTUs.

Tables S8–S10 Polynomial regressions for effect of arsenic on OTU alpha diversity.
