# Supplementary material for: Exposure to Arsenic Alters the Microbiome of Larval Zebrafish
Source: Front Microbiol. 2018 Jun 21;9:1323. doi: 10.3389/fmicb.2018.01323 (PMC6021535; doi:10.3389/fmicb.2018.01323)
Supplement: Figure S1 — Compared observed richness of ASVs and OTUs. [file Image_1.PDF]

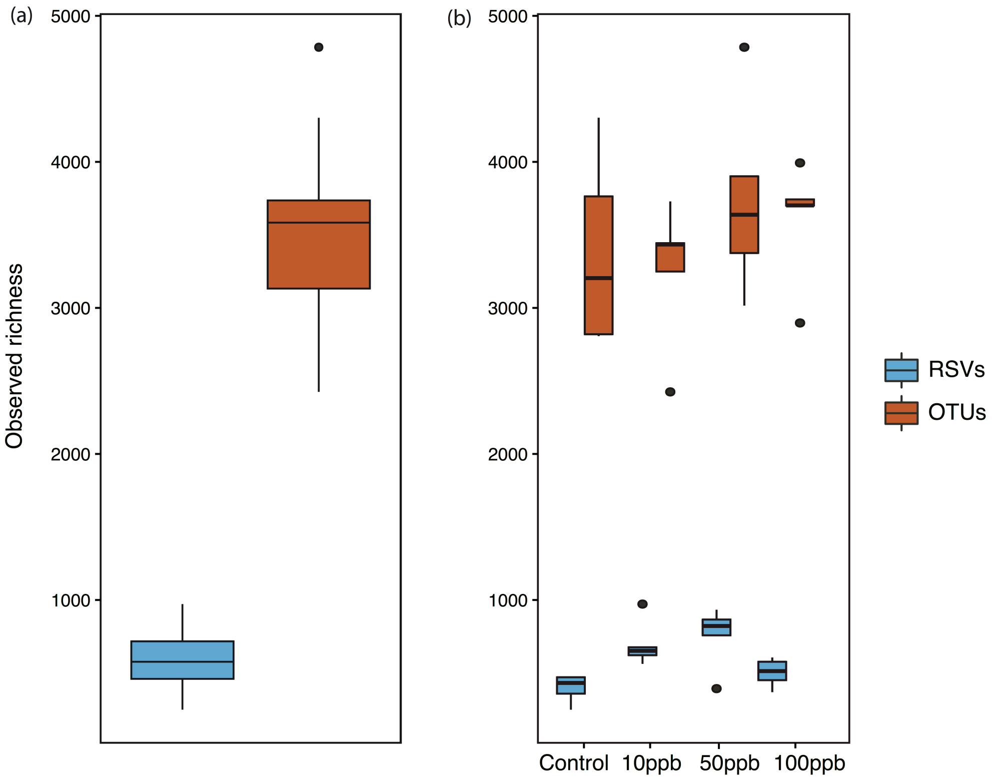

Figure S1. Comparing observed richness of RSVs and OTUs. RSVs were identified using the DADA2 pipeline and OTUs through uclust with the QIIME pipeline. **(a)** Observed richness from all samples. We observed significantly more OTUs than RSVs (Wilcoxon rank sum test; W = 361; P-adj << 0.01), and significantly more variation in amount of OTUs per sample than RSVs (Fligner- Killeen; χ^2 = 6.58; P-adj = 0.026). **(b)** Observed richness by treatment. All comparisons yield significantly more OTUs than RSVs (ANOVA; Tukey HSD; α = 0.01; Table S1). Counts are plotted with median, and hinges as first and third quartiles (25th and 75th percentiles).
