# Supplementary material for: Exposure to Arsenic Alters the Microbiome of Larval Zebrafish
Source: Front Microbiol. 2018 Jun 21;9:1323. doi: 10.3389/fmicb.2018.01323 (PMC6021535; doi:10.3389/fmicb.2018.01323)
Supplement: Figure S2 — OTUs differentially abundant in the presence of arsenic (P ≤ 0.01). [file Image_2.PDF]

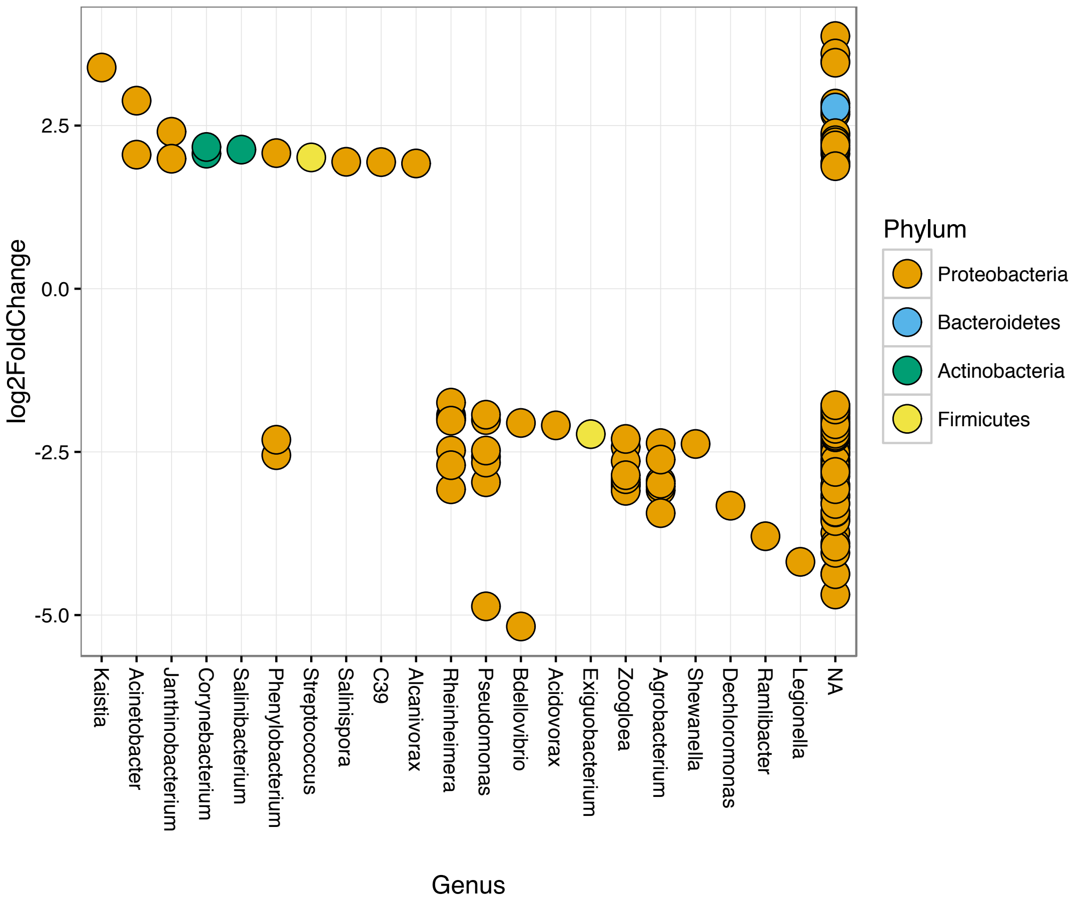

Figure S2. OTUs that significantly differ in abundance in the presence of arsenic. Genus level classification is provided where available. 206 OTUs were identified as significantly different in abundance in the presence of arsenic (DESeq2; adj-*P*s ≤ 0.01).
