# Supplementary material for: Exposure to Arsenic Alters the Microbiome of Larval Zebrafish
Source: Front Microbiol. 2018 Jun 21;9:1323. doi: 10.3389/fmicb.2018.01323 (PMC6021535; doi:10.3389/fmicb.2018.01323)
Supplement: Figure S3 — Alpha diversity metrics, measured with OTUs, with arsenic exposure. [file Image_3.PDF]

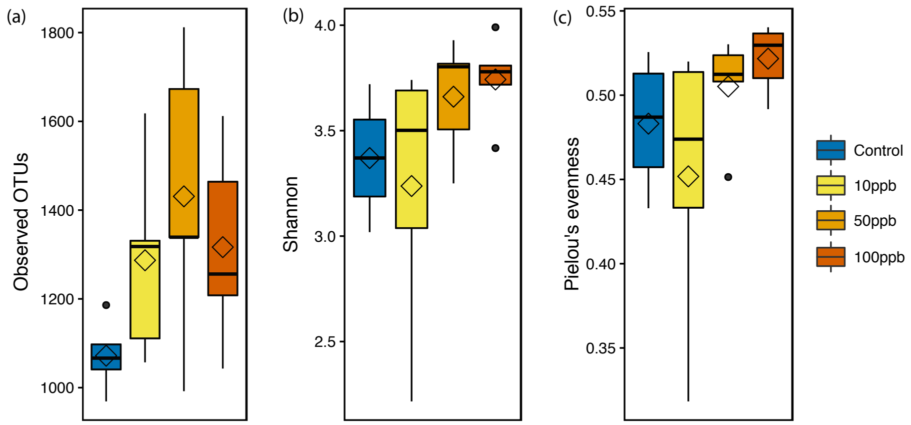

Figure S3. Alpha diversity metrics, measured with OTUs, with arsenic exposure. (**a**) Observed OTUs measurement ($F_{(2,15)}$= 2.12; P = 0.15) (**b**) Shan non diversity measurements ($F_{(2,15)}$ = 2.31; P = 0.13). (**c**) Pielou's evenness measurements ($F_{(2,15)}$= 1.91; P = 0.18). All metrics are plotted against arsenic concentration gradient; with mean (diamond) and median (line), and hinges as first and third quartiles (25th and 75th percentiles).
