# Supplementary material for: Exposure to Arsenic Alters the Microbiome of Larval Zebrafish
Source: Front Microbiol. 2018 Jun 21;9:1323. doi: 10.3389/fmicb.2018.01323 (PMC6021535; doi:10.3389/fmicb.2018.01323)
Supplement: Figure S4 — Principle coordinates analysis (PCoA) based on unweighted UniFrac scores of zebrafish microbiota, using OTU table. [file Image_4.PDF]

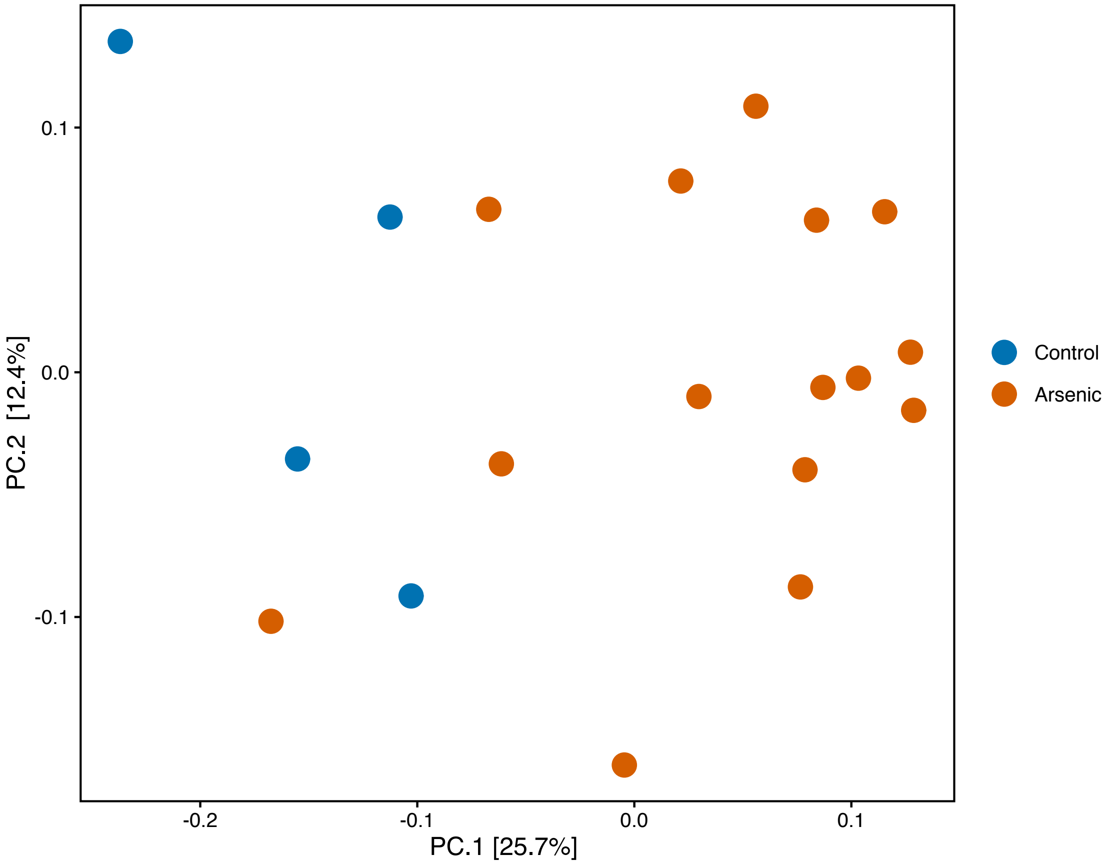

Figure S4. Principle coordinates analysis (PCoA) based on unweighted UniFrac scores of zebrafish microbiota, using OTU table. Analysis revealed that there was significant dissimilarity between the pooled arsenic group and control group ($P < 0.01$, R= 0.547, perm = 999, ANOSIM).
