# Supplementary material for: Exposure to Arsenic Alters the Microbiome of Larval Zebrafish
Source: Front Microbiol. 2018 Jun 21;9:1323. doi: 10.3389/fmicb.2018.01323 (PMC6021535; doi:10.3389/fmicb.2018.01323)
Supplement: Figure S5 — PCoA based on unweighted UniFrac scores of ASVs from zebrafish microbiota amplified for int1. [file Image_5.PDF]

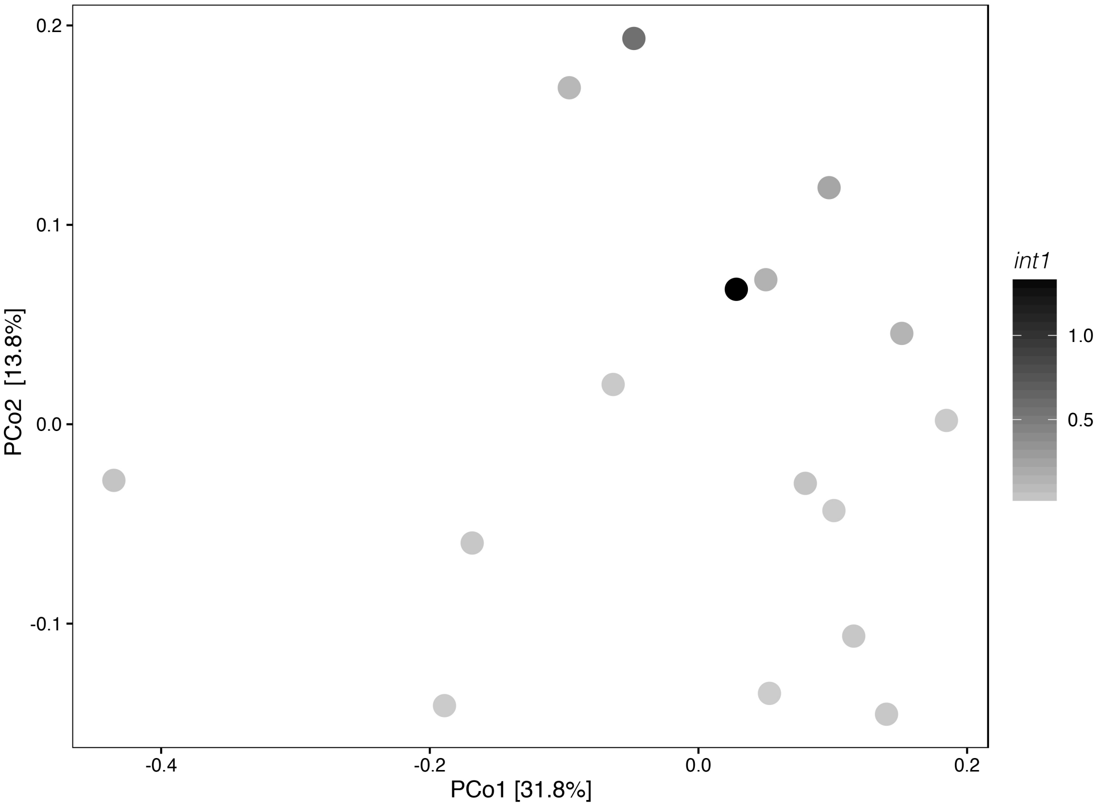

Figure S10. PCoA based on unweighted UniFrac scores of RSVs from zebrafish microbiota amplified for *int1*. No significant dissimilarity between samples with *int1* as an explanatory variable. (ADONIS; R2 = 0.056; *P* = 0.59; perm = 999; n = 16).
