# Supplementary material for: Exposure to Arsenic Alters the Microbiome of Larval Zebrafish
Source: Front Microbiol. 2018 Jun 21;9:1323. doi: 10.3389/fmicb.2018.01323 (PMC6021535; doi:10.3389/fmicb.2018.01323)
Supplement: Datasheet 1 — R markdown file with scripts outlining DADA2 forward read processing. [file Data_Sheet_1.PDF]

Processing forward reads - using DADA2 pipeline

Arsenic Alters the Larval Zebrafish Microbiome, Dahan et al. 2017

October 2017

Contents

Outline of analysis 1

Remove phiX sequences from reads **1**
Build index 1
Align R1 reads against index 2

Split sequences by sample **2**

DADA2 **2**
Loading packages and grabbing files 3
Quality visualization 3
Filter and trim reads 4
Dereplication 4
Sample inference 5
Make sequence table and identify and remove chimeras 6
Assign Taxonomy 8

Build a multiple sequence alignment and phylogenetic tree **8**
Multiple sequence alignment 10
Build tree 11
Handoff to phyloseq and add metadata 11
Metadata 11
Make phyloseq object 12

Outline of analysis

Here is our main forward read processing analysis. This includes removing phiX with Bowtie2, splitting sequences by sample in QIIME, processing and filtering reads with DADA2, building a multiple sequence alignment with PyNAST, a phylogenetic tree with FastTree, and assigning taxonomy with DADA2's native implementation of the RDP classifier.

Remove phiX sequences from reads

In order to increase sequence diversity and reduce sequencing errors, we added phiX to our 16S rRNA reads. We use bowtie2 to remove phiX sequences from sequence reads. This speeds things up significantly for DADA2 and will make analyses more accurate and relevant. **The following steps are conducted in bash** and require Bowtie2.

Build index

With the phiX genome downloaded from illumina support we create index for the phiX reference genome with the following commmand. The output files will all start with phix and end with .1.bt2, .2.bt2 etc..

```
#Path to your phiX genome
MacQIIME Macintosh-5:bowtie_dada2_phylo $ bowtie2-build /Users/Dylan/Documents/QIIME_fish/forward_read_a
```

Align R1 reads against index

Now we align our forward reads (R1s) against the phix index and create two output files, one with the R1s that aligned to phiX and another with reads failing alingment. These failed reads no longer contain phiX and are what we are going to use for downstream processing/analyses.

```
MacQIIME Macintosh-5:bowtie_dada2_phylo $ bowtie2 -x phiX -U /Users/Dylan/Documents/QIIME_fish/forward_
```

- -x -> index for the phiX reference genome. Basically referencing all the files we created in the previous step that start with phiX
- -U -> input fastq file. In this case it is a path the our forward reads in a different directory
- -S -> our output fastq file, which only contains reads that aligned to the phiX reference genome. So, these are only phiX reads.
- –un -> our output reads that fail to align to the phiX genome. You can pass an additional command of gz (–un-gz) if you want this output to be zipped.

The output should read:

```
0108755 reads; of these:
10108755 (100.00%) were unpaired; of these:
9922551 (98.16%) aligned 0 times
186204 (1.84%) aligned exactly 1 time
0 (0.00%) aligned >1 times
1.84% overall alignment rate
```

This is telling us that ~2.0% of our reads were phiX!

Split sequences by sample

To process our R1s in DADA2 we need to split them by sample. This is also conducted in terminal and requires and QIIME. We can do this with the QIIME scipt `split_sequence_file_on_sample_ids.py`.

```
#Make a new directory
Macintosh-5:bowtie_dada2_phylo Dylan$ mkdir R1_nophix_split_by_sample/
#Split seqs by sample
Macintosh-5:bowtie_dada2_phylo Dylan$ split_sequence_file_on_sample_ids.py -i  R1_seqs_nophix.fastq --fi
```

- -i -> input fastq file
- -o -> output directory for all the sequences split by sample

DADA2

Now we are working in R. And, methods and most documentation taken from Ben Callahan's DADA2 tutorial

In short: * Prefiltering - using a filtering threshold rather than quality scores * Dereplication - combines all identical reads into unique sequences and builds consensus quality scores * Sample inference - infers samples error rates and the likelihood that a low abundance sequence is due to errors * Build sequence table * Remove bimeras (two parent chimeras) * Assign taxonomy

Loading packages and grabbing files

If you don't have ShortRead, DADA2, phyloseq they can be installed via Bioconductor, and ggplot2, plyr, dplyr, and grid are available from CRAN.

```
.bioc_packages <- c("dada2","phyloseq","ShortRead")
.cran_packages <- c("dplyr","plyr","ggplot2","grid")

.inst <- .bioc_packages %in% installed.packages()
if(any(!.inst)) {
   source("http://bioconductor.org/biocLite.R")
   biocLite(.bioc_packages[!.inst], ask = F)
}

.inst <- .cran_packages %in% installed.packages()
if(any(!.inst)) {
   install.packages(.cran_packages[!.inst])
}

sapply(c(.bioc_packages,.cran_packages), require, character.only = TRUE)
```

```
##     dada2  phyloseq ShortRead     dplyr      plyr   ggplot2      grid
##      TRUE      TRUE      TRUE      TRUE      TRUE      TRUE      TRUE
```

```
set.seed(100)
```

Switch to the path with your R1 and make a list of files and then sort files to an object to only contain R1s with phiX removed.

```
path<-("~/Documents/QIIME_fish/forward_read_analysis/bowtie_dada2_phylo/R1_nophix_split_by_sample/")
fns<-list.files(path)
#Grab all the fastq files from this path
fastqs <- fns[grepl('fastq$',fns)]
fnFs <- fastqs[grepl(".fastq", fastqs)]
#Sort them
fnFs<-sort(fnFs)
fnFs<-paste0(path,fnFs)
```

Quality visualization

Visualize the quality of each file, with Q score on y-axis and position on x-axis. Visual inspection can suggest a trim length and which samples to omit.

```
#This shows quality profliing for the first read in our list, but to see all of them you can replace th
plotQualityProfile(fnFs[1])
```

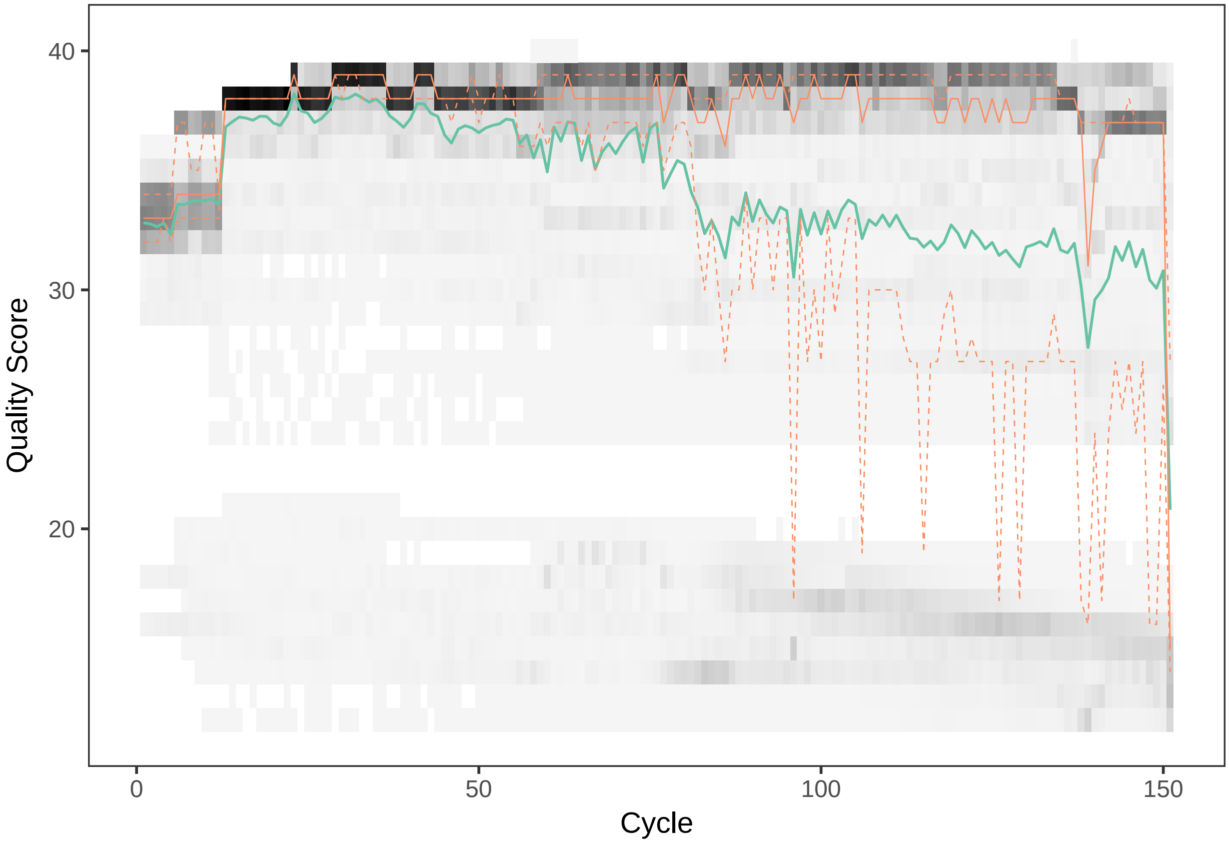

We see from the visualization that we have a pretty high Phred (>30) score throughout with a dropoff ~140bp. Illumina sequences normally have high error rates and low quality where they attach to the platform, towards the beginning and end of the reads, and so it is advised to trim the beginning and ends of the reads.

Filter and trim reads

Explanation of filtering taken from DADA2 tutorial:

"The filtering parameters we'll use are standard: maxN=0 (DADA2 requires no Ns), truncQ=2 (quality score 2 in Illumina means "stop using this read") and maxEE=2. The maxEE parameter sets the maximum number of "expected errors" allowed in a read."

In each of the samples we see how many reads were input and how many filtered sequences we output. We also see that one sample, DahanA2, only has 8 reads! This was likely due to a sequencing error and we will remove it later.

Dereplication

Again, the DADA2 tutorial explains this expertly so the following blurb is taken from the tutorial:

"In the dereplication step, all reads with identical sequences are combined into "unique sequences" with a corresponding abundance, i.e. the number of reads with that same sequence. Dereplication is a part of most pipelines because it reduces computation time by eliminating redundant comparisons between sequences."

"Dereplication in the DADA2 pipeline has one crucial addition: DADA2 retains a summary of the quality information associated with each unique sequence. DADA2 constructs a "consensus" quality profile for each

unique sequence by averaging the positional qualities from the dereplicated reads. These consensus quality profiles inform the error model of the subsequent denoising step, significantly increasing DADA2's accuracy."

```
## derep-class: R object describing dereplicated sequencing reads
## $uniques: 477679 reads in 169892 unique sequences
##   Sequence lengths: min=130, median=130, max=130
## $quals: Quality matrix dimension:  169892 130
##   Consensus quality scores: min=12, median=37, max=40
## $map: Map from reads to unique sequences:  86840 73243 2 134553 12426 ...
```

Sample inference

Sample inference via the core sample inference algorithm. See documentation on `dada` for details. This step took about 20h on my laptop, which uses the intel i3, has 2 cores and 8gb of ram. Alternatively, to speed things up you can infer error rates from one sample and the process the rest of the reads with these inferences.

Blurb taken from DADA2 tutorial: *"an EM-like algorithm in which the error rates and the sample are alternately estimated until convergence."*

"To perform this joint inference with dada(. . .) we pass it the selfConsist=TRUE flag, and specify the errorEstimationFunction = loessErrfun (the current default option). As is common in optimization problems we still must provide an initial guess at the error rates. For this we take a previously estimated set of error rates (tperr1, included with the package) and inflate them, as it is better to start with error rates that are too high than too low."

```
## Sample 1 - 477679 reads in 169892 unique sequences.
## Sample 2 - 481668 reads in 257179 unique sequences.
## Sample 3 - 515487 reads in 289652 unique sequences.
## Sample 4 - 595801 reads in 303263 unique sequences.
## Sample 5 - 8 reads in 8 unique sequences.
## Sample 6 - 379824 reads in 111497 unique sequences.
## Sample 7 - 308063 reads in 79423 unique sequences.
## Sample 8 - 401847 reads in 103906 unique sequences.
## Sample 9 - 342079 reads in 103844 unique sequences.
## Sample 10 - 563065 reads in 251853 unique sequences.
## Sample 11 - 669222 reads in 460964 unique sequences.
## Sample 12 - 500349 reads in 274284 unique sequences.
## Sample 13 - 532297 reads in 273558 unique sequences.
## Sample 14 - 642340 reads in 481701 unique sequences.
## Sample 15 - 395601 reads in 174187 unique sequences.
## Sample 16 - 869527 reads in 783069 unique sequences.
## Sample 17 - 787802 reads in 669810 unique sequences.
## Sample 18 - 328968 reads in 168573 unique sequences.
## Sample 19 - 568811 reads in 479389 unique sequences.
## Sample 20 - 435376 reads in 215884 unique sequences.
##    selfConsist step 2
##    selfConsist step 3
##    selfConsist step 4
##    selfConsist step 5
##
##
## Convergence after  5  rounds.
```

```
## $`~/Documents/QIIME_fish/forward_read_analysis/bowtie_dada2_phylo/R1_nophix_split_by_sample/DahanA1`
## dada-class: object describing DADA2 denoising results
## 2493 sample sequences were inferred from 169892 input unique sequences.
```

```
## Key parameters: OMEGA_A = 1e-40, BAND_SIZE = 16, USE_QUALS = TRUE
```

Above we can see that the dada method inferred 2512 real variants from 170778 unique sequences in the first sample! These are our ribosomal sequences variants (RSVs).

An earlier version of this manuscript called RSVs ISeVs, so where ISeV is used, know it is synonymous with RSV

We can then visualize these erorr rates for all possible nucleotide errors:

```
#Just plotting for samp 15, but can do any!
plotErrors(dada.consist[[15]],nominalQ=TRUE)
```

```
## Warning: Transformation introduced infinite values in continuous y-axis

## Warning: Transformation introduced infinite values in continuous y-axis
```

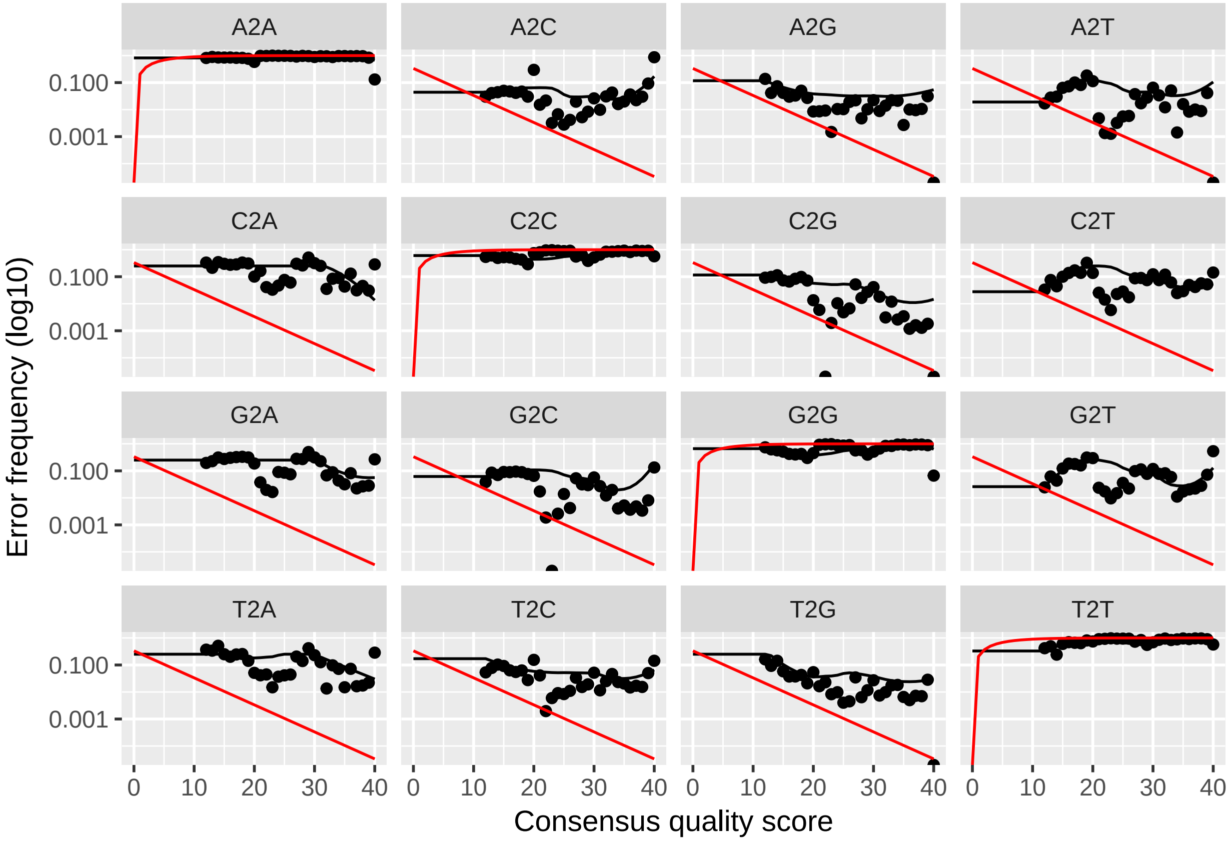

As we can see, there should be decreased error rates with increased quality scores. Our data does have pretty high observed error rates (points) but our estimate error rates (black line) do fit these observed values well. Though it is a bit concerning that we have higher error rates than expected "under the nominal defintion of the Q score" (red line), it is good that the estimates do fit our observed error rates, being that the model worked well, and so we proceed from here.

Make sequence table and identify and remove chimeras

Make a sequence table and then identify and remove bimeras Also, make a RSV table.

Making sequence table:

```r
seqtab <-makeSequenceTable(dada.consist)
```

What are the dimension of are sequence table? Should be the samples (20 in this case) by the RSVs (12577 here)

```r
dim(seqtab)
```

```
## [1]    20 12454
```

Looks good.

Next we identify and remove bimeras with the removeBimeraDenovo command. See the DADA2 tutorial and `?isBimeraDenovo` documentation for more information. There can be a lot of chimeras in our sequence table!

```r
seqtab.nochim <- removeBimeraDenovo(seqtab,verbose = TRUE)
```

```
## As of the 1.4 release, the default method changed to consensus (from pooled).
```

```
## Identified 8758 bimeras out of 12454 input sequences.
```

What proportion of the sequence variants were chimeras?

```r
1-dim(seqtab.nochim)[2]/dim(seqtab)[2]
```

```
## [1] 0.7032279
```

~74% is a lot of chimeric sequences! But another important question is what proportion of of the total sequence reads were chimeras?

```r
1 - sum(seqtab.nochim)/sum(seqtab)
```

```
## [1] 0.1041467
```

Since ~74% of the sequences were chimeras but ~12% of the total reads were chimeras this is suggesting that although a good amount of all the sequences were chimeras, they in fact did not make up a substantial proportion of our table.

We can also now see how many sequences we have in all:

```r
dim(seqtab.nochim)[2]
```

```
## [1] 3696
```

Removing chimeras was our final step in filtering our reads. We have now removed phiX, filtered low quality reads, corrected amplicon errors and removed chimeras. What percent of our total reads have we retained? The forward reads originally returned 10,108,755 reads. We can see this from the phiX output above or by **running the following in terminal.**

```
#Path to original fastq file before filtering out phiX
grep -c "@Dahan" /Users/Dylan/Documents/QIIME_fish/forward_R1_seqs/R1_seqs.fastq`
#Outputs 10108755
#Alternatively, for a quicker runtime you can count the number of line breaks and divide by 4
Macintosh-24:~ Dylan$ wc -l /Users/Dylan/Documents/QIIME_fish/forward_R1_seqs/R1_seqs.fastq
#Outputs 40435020 and 40435020/4 = 10108755
```

Then we can find out what proportion of seqs we retained after the DADA2 pipeline by dividing our new sequence sum over the original.

```r
sum(seqtab.nochim)/10108755
```

```
## [1] 0.86812
```

After processing our forward reads with DADA2 we retain ~86% of our reads. Great!

Assign Taxonomy

The latest version of DADA2 (v.0.99.5) has wonderfully supplied a native implemetation of the RDP's naive Bayesion classifier for assigning taxonomy. We assign taxonomy to our sequences using the GreenGenes 13.8 training, as was used when defining the core microbiome in zebrafish. Your reference dataset should be study specific, so see which one (RDP or GreenGenes or Silva) might be most appropriate. Download the appropriate dataset and run the `assignTaxonomy` function. Links to the different datasets can be found in the DADA2 tutorial.

```
#Before adding taxonomy and all let's just make our rownames our intended sample names
rownames(seqtab.nochim) = names(dada.consist)

#Assigned taxanomy against the Greengenes database.
taxa <- assignTaxonomy(seqtab.nochim, paste0(path, "gg_13_8_train_set_97.fa.gz"))
colnames(taxa) <- c("Kingdom", "Phylum", "Class", "Order", "Family", "Genus","Species")
```

Let's see who is most represented in these samples..

```
unname(head(taxa))
```

```
##      [,1]          [,2]                 [,3]
## [1,] "k__Bacteria" "p__Proteobacteria" NA
## [2,] "k__Bacteria" "p__Proteobacteria" "c__Gammaproteobacteria"
## [3,] "k__Bacteria" "p__Proteobacteria" "c__Gammaproteobacteria"
## [4,] "k__Bacteria" "p__Proteobacteria" "c__Betaproteobacteria"
## [5,] "k__Bacteria" "p__Bacteroidetes"  "c__[Saprospirae]"
## [6,] "k__Bacteria" "p__Proteobacteria" "c__Alphaproteobacteria"
##      [,4]                  [,5]                   [,6]
## [1,] NA                    NA                     NA
## [2,] "o__Aeromonadales"    "f__Aeromonadaceae"    "g__"
## [3,] "o__Legionellales"    "f__"                  "g__"
## [4,] "o__Burkholderiales"  "f__Comamonadaceae"    "g__Limnohabitans"
## [5,] "o__[Saprospirales]"  "f__Chitinophagaceae"  "g__Sediminibacterium"
## [6,] "o__Sphingomonadales" "f__Sphingomonadaceae" "g__Sphingomonas"
##      [,7]
## [1,] NA
## [2,] "s__"
## [3,] "s__"
## [4,] "s__"
## [5,] "s__"
## [6,] NA
```

Considering these are zebrafish microbiomes, and Proteobacteria are common to aquatic environments and fish microbiomes, there are no suprises here.

Build a multiple sequence alignment and phylogenetic tree

The following steps are conducted first in R and then in bash

After first preparing our files in R, we switch to bash and build a multiple sequence alignment using PyNAST. Next, we use FastTree to infer an approximately-maximum-likelihood phylogenetic tree. We carry out these steps in QIIME with the align_seqs.py and make_phylogeny.py scripts.

First we want to turn this set of RSVs into a fasta file. This is analagous to the "rep_set" or representative sequence file that is made in the QIIME pipeline. However, with DADA2 this is not an approximate

representative set of sequences that are at least 97% similar to their clusters but rather inferred sequence variants at 100% identity, and thus and taxonomic assignments to them are applicable to all sequences of their set and do not run the risk of being polyphyletic.

Make our representative fasta file:

```
uniquesToFasta(getUniques(seqtab.nochim), "~/Documents/QIIME_fish/forward_read_analysis/bowtie_dada2_phy
```

See how many counts per sequence in fasta file:

```
#File path to your fasta file
isevs = read.csv("~/Documents/QIIME_fish/forward_read_analysis/bowtie_dada2_phylo/R1_nophix_split_by_sam
#Print the head lines of this file
head(isevs)
```

```
##
## 1 TCTAGTTGATATACTACGGCGTAAAGGGTGGTTAAGGAACAATGTAAAATAGAGCCAAACGGCCCTTTAACTGTTATACGCTTTTAGATGCTAGAGGC
## 2
## 3 GCAAGCGTTAATCGGAATTACTGGGCGTAAAGCGCACGCAGGCGGTTGGATAAGTTAGATGTGAAAGCCCCGGGCTCAACCTGGGAATTGCATTTAAA
## 4
## 5 GCGAGCGTTAATCGGAATTACTGGGCGTAAAGCGTGTGTAGGTGGTTAGATAAGTTAGATGTGAAATCCCCGGGCTTAACCTGGGCACTGCGTTTAAG
## 6
```

In the sequence headers (e.g., >sq2;size=1391516) we can see the amount of sequences per sequence variant. We want to note these, rename the fasta file and RSV columns to match one another and then check to make sure these haven't changed.

Open up terminal and change to your directory with the newly made fasta file and rename this to simple sq1,sq2,sq3 etc... We're doing this for a couple of reasons.
1. FastTree makes it clear that the characters *:,()* should not appear in sequence names.
2. We are going to match this tree to our RSVs for our analyses relying on trees (e.g., Unifrac) and thus must make sure the sequences and tree tips have the same names.

```
Macintosh-24:R1_nophix_split_by_sample Dylan$ perl -ane 'if(/\>/){$a++;print ">sq$a\n"}else{print;}' is
```

Likewise, we are doing the same for our column names in our sequence table. It is crucial we do this because when we import these files in phyloseq the tip labels of the tree must match the inferred sequences names of the sequence table.

```
#Make a repeated list of number from 1 to the amount of sequences in our sequence table
sqrep = rep(1:dim(seqtab.nochim)[2])
#Paste sq before each of these numbers
sqrep = paste("sq",sqrep,sep="")
#Duplicate our seqtab.nochim file
seqtab.nochim.sq <- seqtab.nochim
#Save this sequence table object for later use
saveRDS(seqtab.nochim.sq,"seqtab.nochim.sq.Rdata")

#Make the sequence names in this file sq1, sq2, sq3 etc.. by using our new continuous sq list
colnames(seqtab.nochim.sq) <- sqrep

colnames(seqtab.nochim.sq)[1:5]
```

```
## [1] "sq1" "sq2" "sq3" "sq4" "sq5"
```

```
#Duplicate our taxa file
taxa.sq <- taxa
#Change the sequence names in our taxa file to sq1, sq2, sq3 etc...
rownames(taxa.sq) <- sqrep
```

```r
rownames(taxa.sq)[1:5]
```

```
## [1] "sq1" "sq2" "sq3" "sq4" "sq5"
```

```r
#Save taxa table for later use
saveRDS(taxa.sq,'taxa.sq.Rdata')
```

Sum of the most abundant reads?

```r
sum(seqtab.nochim.sq[,1])
```

```
## [1] 2279592
```

```r
sum(seqtab.nochim.sq[,2])
```

```
## [1] 1386706
```

```r
sum(seqtab.nochim.sq[,3])
```

```
## [1] 1061999
```

Now let's check to make sure that the sequences contain the same labels:

```r
#Read new renamed representative inferred sequence file
isevs.renamed = read.csv("~/Documents/QIIME_fish/forward_read_analysis/bowtie_dada2_phylo/R1_nophix_spli

head(isevs)
```

```
##
## 1 TCTAGTTGATATACTACGGCGTAAAGGGTGGTTAAGGAACAATGTAAAATAGAGCCAAACGGCCCTTTAACTGTTATACGCTTTTAGATGCTAGAGGC
## 2
## 3 GCAAGCGTTAATCGGAATTACTGGGCGTAAAGCGCACGCAGGCGGTTGGATAAGTTAGATGTGAAAGCCCCGGGCTCAACCTGGGAATTGCATTTAAA
## 4
## 5 GCGAGCGTTAATCGGAATTACTGGGCGTAAAGCGTGTGTAGGTGGTTAGATAAGTTAGATGTGAAATCCCCGGGCTTAACCTGGGCACTGCGTTTAAG
## 6
```

```r
head(isevs.renamed)
```

```
##
## 1 TCTAGTTGATATACTACGGCGTAAAGGGTGGTTAAGGAACAATGTAAAATAGAGCCAAACGGCCCTTTAACTGTTATACGCTTTTAGATGCTAGAGGC
## 2
## 3 GCAAGCGTTAATCGGAATTACTGGGCGTAAAGCGCACGCAGGCGGTTGGATAAGTTAGATGTGAAAGCCCCGGGCTCAACCTGGGAATTGCATTTAAA
## 4
## 5 GCGAGCGTTAATCGGAATTACTGGGCGTAAAGCGTGTGTAGGTGGTTAGATAAGTTAGATGTGAAATCCCCGGGCTTAACCTGGGCACTGCGTTTAAG
## 6
```

Great! We can see that we have matching labels for our sequences in the represenative infered sequence file and our column headers in the sequence table.

Multiple sequence alignment

Use PyNAST to build a multiple sequence alingment. This is done in terminal not in R. Alternatively you could use muscle or other alignment tools, but without a template alignment the runtime can be a bit long. Beware of your blast cutoff to your template aligment since high cutoff thresholds may result in sequences (and possibly top ranking sequences, as it was with this data) being omitted from the alignment and thus your tree and phylogeny based diversity comparisons. Here we use the greengenes alignment with 55% as a cutoff. This is a low threshold, but later we blast sequences against zebrafish DNA to remove possible contaminants.

```
#Make sure you're in the correct directory
MacQIIME Macintosh-24:R1_nophix_split_by_sample $ align_seqs.py -i isevs_renamed.fasta  -o align_seqs/ 
```

Build tree

Then we run FastTree to infer an approximately-maximum-likelihood phylogenetic tree from our alignment. This is QIIME's default building method.

```
MacQIIME Macintosh-24:R1_nophix_split_by_sample $ make_phylogeny.py -i align_seqs/isevs_renamed_aligned
```

Handoff to phyloseq and add metadata

For the first step we are building metadata for our sequences by writing out our sample names and importing some metadata that was reported during the study and constructed in excel. Its a pretty simple file with column headers as metadata variables and rows as samples.

Metadata

```
#Writing out our sample names
samples.out <- rownames(seqtab.nochim)

#Import metdata. These should also have your sample names so that when importing them you can make sure
metad <- read.csv('~/Documents/QIIME_fish/forward_read_analysis/bowtie_dada2_phylo/R1_nophix_split_by_sa

#How does our metadata look?
metad
```

```
##    SampleID Treatment AsConc Treatment_alph PresAbs Description De_factor
## 1   DahanA1   Control      0              a Control     DahanA1    absent
## 2  DahanA10     10ppb     10              b Arsenic    DahanA10   present
## 3  DahanA11     50ppb     50              c Arsenic    DahanA11   present
## 4  DahanA12     50ppb     50              c Arsenic    DahanA12   present
## 5   DahanA2   Control      0              a Control     DahanA2    absent
## 6   DahanA3   Control      0              a Control     DahanA3    absent
## 7   DahanA4   Control      0              a Control     DahanA4    absent
## 8   DahanA5   Control      0              a Control     DahanA5    absent
## 9   DahanA6     10ppb     10              b Arsenic     DahanA6   present
## 10  DahanA7     10ppb     10              b Arsenic     DahanA7   present
## 11  DahanA8     10ppb     10              b Arsenic     DahanA8   present
## 12  DahanA9     10ppb     10              b Arsenic     DahanA9   present
## 13  DahanB1     50ppb     50              c Arsenic     DahanB1   present
## 14  DahanB2     50ppb     50              c Arsenic     DahanB2   present
## 15  DahanB3     50ppb     50              c Arsenic     DahanB3   present
## 16  DahanB4    100ppb    100              d Arsenic     DahanB4   present
## 17  DahanB5    100ppb    100              d Arsenic     DahanB5   present
## 18  DahanB6    100ppb    100              d Arsenic     DahanB6   present
## 19  DahanB7    100ppb    100              d Arsenic     DahanB7   present
## 20  DahanB8    100ppb    100              d Arsenic     DahanB8   present
##           int1
## 1         <NA>
## 2  0.049601407
## 3  0.009343824
```

```
## 4  0.147134776
## 5  0.116874742
## 6  0.034567927
## 7  0.048260372
## 8  0.007808735
## 9           NA
## 10 0.002283649
## 11          NA
## 12 0.026945112
## 13 0.034107247
## 14 0.013675693
## 15 0.021503047
## 16 1.342358962
## 17 0.228928924
## 18 0.569488991
## 19 0.155760142
## 20 0.121098659
```

```
#Name the rows after our samples from the DADA2 workflow.
rownames(metad) <- samples.out

#Save new metadata to R object for later use in phyloseq
saveRDS(metad,'~/Documents/QIIME_fish/forward_read_analysis/bowtie_dada2_phylo/R1_nophix_split_by_sampl
```

Pretty good. We have sample names and the appropriate metadata, like arsenic concentration.

Make phyloseq object

Now we make our phyloseq object by importing the tree and making object from our sequence table, phylogenetic tree, metadata and taxa table. We also prune samples that don't have more than 100 reads. In this case, we do this to remove the one sample that failed sequencing. We also make an object without our phylogenetic tree to retain all of our RSVs, which will be used to compare raw RSVs output from DADA2 v. OTUs from uclust.

```
#Import tree using the ape package
phytpy <- ape::read.tree("~/Documents/QIIME_fish/forward_read_analysis/bowtie_dada2_phylo/R1_nophix_spl


pspy <- phyloseq(otu_table(seqtab.nochim.sq, taxa_are_rows=FALSE),
               phytpy,
               sample_data(metad),
               tax_table(taxa.sq))
#Remove sample DAHANA2, which failed sequencing and only returned 8 reads
pspy = prune_samples(sample_sums(pspy)>100,pspy)
saveRDS(pspy, "~/Documents/QIIME_fish/forward_read_analysis/bowtie_dada2_phylo/R1_nophix_split_by_sampl

#Also save an object with sequences that were removed in the mutliple alignment. This is ONLY so that w
psnopy <- phyloseq(otu_table(seqtab.nochim.sq, taxa_are_rows=FALSE),
               sample_data(metad),
               tax_table(taxa.sq))
#Remove sample DAHANA2, which failed sequencing and only returned 8 reads
psnopy = prune_samples(sample_sums(psnopy)>1000,psnopy)
saveRDS(psnopy, "~/Documents/QIIME_fish/forward_read_analysis/bowtie_dada2_phylo/R1_nophix_split_by_sam
```

This is the end of the DADA2 processing methods. Next we use this phyloseq object and move onto the

phyloseq/DESeq2/vegan analysis.
