# Supplementary material for: Exposure to Arsenic Alters the Microbiome of Larval Zebrafish
Source: Front Microbiol. 2018 Jun 21;9:1323. doi: 10.3389/fmicb.2018.01323 (PMC6021535; doi:10.3389/fmicb.2018.01323)
Supplement: Datasheet 2 — R markdown file with scripts outlining community analyses. [file Data_Sheet_2.PDF]

phyloseq/DESeq2/vegan analysis on RSVs

Arsenic Alters the Larval Zebrafish Microbiome, Dahan et al. 2017

October 2017

Contents

Load libraries and phyloseq object . . . 1

Preprocessing and taxa composition visualization **2**
- Filter taxa . . . 2
- Define order plotting function . . . 3
- Plot phylum . . . 5
- Plot family . . . 7

Core microbiome **9**

Alpha diversity analyses **12**
- Calculate alpha diversity . . . 12
- Make models and plot alpha diversity . . . 13

Beta diversity analyses **25**
- Run and plot ordinations . . . 25
- Run ANOSIM and ADONIS . . . 27
- Biplot . . . 30

DESeq2 differential abundance **32**
- Differential abundance analysis and plotting dispersion estimates . . . 32
- Extract differential abundance results . . . 33
- Plot DESeq2 results . . . 35

Alternative analysis on QIIME processed OTU table (clustered w/ USEARCH) **37**

Here is an analysis on our ribosomal sequence variants (RSVs) following our forward read processing with DADA2. We use several packages, primarily including phyloseq, vegan and DESeq2. We begin by applying some pre-processing to our ISeV table (via phyloseq object) and then move on to visualizing taxonomy and analysing the core microbiome, alpha diversity, beta diversity and ISeV differential abundance. At the end you can also see how to load QIIME objects and run an OTU table through the same pipeline.

IN AN EARLIER VERSION OF THIS MANUSCRIPT WE CALLED RSVs ISeVs, SO IF SEEN, THE TWO ARE INTERCHANGEABLE

Load libraries and phyloseq object

```
library(phyloseq);packageVersion("phyloseq")
```

```
## [1] '1.20.0'
```

```
library(ggplot2);packageVersion("ggplot2")
```

```
## [1] '2.2.1'
```

```r
library(ShortRead);packageVersion("ShortRead")
```

```
## [1] '1.34.1'
```

```r
library(data.table);packageVersion("data.table")
```

```
## [1] '1.10.4'
```

```r
library(plyr);packageVersion("plyr")
```

```
## [1] '1.8.4'
```

```r
library(vegan);packageVersion("vegan")
```

```
## [1] '2.4.4'
```

```r
library(grid);packageVersion("grid")
```

```
## [1] '3.4.1'
```

```r
#library(optparse);packageVersion("optparse")
library(DESeq2);packageVersion("DESeq2")
```

```
## [1] '1.16.1'
```

```r
#library(biom);packageVersion("biom")


pspy <- readRDS("~/Documents/QIIME_fish/forward_read_analysis/bowtie_dada2_phylo/R1_nophix_split_by_samp[illegible]

set.seed(9999)
```

Preprocessing and taxa composition visualization

Filter taxa

First, we will remove taxa that were identified as zebrafish mitochondria (zebrafish gDNA). We noticed the possbility that 16S primers can amplify zebrafish mitochondria and some of our sequences are identified as mitochondria and thus after a blast and identification, we remove these. Evolutionarily, this is pretty cool and interesting, but for the sake of investigating microbial diversity we must remove them. These steps require blast

How to remove these mitochondrial sequences (done in terminal and then R):

In terminal first make a blast database from the zebrafish mitochondria sequences.

```
dylan@zoo-hydra:~/zebrafish_16S$ makeblastdb -in zfishmito.fasta -dbtype nucl -out zfishmito
```

Then blast the list of inferred sequence variants agains the database. Descriptions of the various parameters can be found in the blast command line applications user manual

```
dylan@zoo-hydra:~/zebrafish_16S$ blastn -query isevs_renamed.fasta -db zfishmito -task blastn-short -eva[illegible]
```

294 sequences were identified here.

Then remove make a list with the names of these sequences (e.g., sq1, sq20,sq23)

```
dylan@zoo-hydra:~/zebrafish_16S$ cut -f1 zfishmito_blast > zfish_mito_isevs.csv
```

We then read this csv and remove them using phyloseq's `prune_taxa` function. This part is done in R.

```
#load csv with mitochondrial seq names
zmito = read.csv('~/Documents/QIIME_fish/forward_read_analysis/bowtie_dada2_phylo/R1_nophix_split_by_sa[illegible]
zmito = as.character(as.list(zmito)$V1)
#make a list of all taxa
allTaxa = names(sort(taxa_sums(pspy),TRUE))

#remove zebfrafish mitochondria seqs from the taxa
myTaxa = allTaxa[!allTaxa %in% zmito]


#make a new phyloseq object sans zebfrafish mitochondria
pspy <- prune_taxa(myTaxa,pspy)
```

Preprocess samples to remove taxa not seen at least 3 times in at least 15% of the samples and make a sequence table with relative abundance. This preprocessing allows us to visualize and analyze high-level patterns in the dataset, in specific with the following taxa plots and beta diversity measurements.

In the initial filtering step we protect against an OTU (or in this case, ISeV) with small mean & trivially large C.V.. In the phyloseq tutorial they filter to taxa seen at least 3 times at least 20% of samples, but we chose 15% since we have many fewer taxa to begin with (2873 here vs. 19216 in the tutorial) and all of our singletons were either removed or corrected to real sequence variants with DADA2. Preprocessing steps, however, are dataset dependent.

```
#Filter
psF = filter_taxa(pspy, function(x) sum(x>2) > (0.15*length(x)), TRUE)

#Take relative abundance
psFR = transform_sample_counts(psF, (function(x) x/sum(x)))
```

Define order plotting function

Define a function for plotting purposes

This is implementing a user developed function called plot_ordered_bar. Basically what it does is rather than plot the abundance starting from the bottom with the most abundant and decreasing upwards, it stacks the same colors on top of one another in each column, in a way, allowing more related bacteria to be side by side.

```
# Make some example data
#Set seed
set.seed(22886)
# load phyloseq module
library(phyloseq)
# load ggplot2
library(ggplot2)
# Make some data
otumat = matrix(sample(1:100, 100, replace = TRUE)
                , nrow = 10, ncol = 10)
rownames(otumat) <- paste0("OTU", 1:nrow(otumat))
colnames(otumat) <- paste0("Sample", 1:ncol(otumat))
taxmat = matrix(sample(letters, 70, replace = TRUE),
                nrow = nrow(otumat),
```

```r
                ncol = 7)
rownames(taxmat) <- rownames(otumat)
colnames(taxmat) <- c("Domain",
                      "Phylum",
                      "Class",
                      "Order",
                      "Family",
                      "Genus",
                      "Species")
OTU = otu_table(otumat, taxa_are_rows = TRUE)
TAX = tax_table(taxmat)
physeq = phyloseq(OTU, TAX)

########
# Plot_ordered_bar function
# (name kept the same for back compatibility
# may want to change to avoid confusion!)
#Note: There may be some hangovers
# from another script I was writing
# that I pillaged for this one

###### Start of Function Here ######

plot_ordered_bar <- function (physeq, x = "Sample",
                              y = "Abundance",
                              fill = NULL,
                              leg_size = 0.5,
                              title = NULL) {
  require(ggplot2)
  require(phyloseq)
  require(plyr)
  require(grid)
  bb <- psmelt(physeq)

  samp_names <- aggregate(bb$Abundance, by=list(bb$Sample), FUN=sum)[,1]
  .e <- environment()
  bb[,fill]<- factor(bb[,fill], rev(sort(unique(bb[,fill])))) #fill to genus


  bb<- bb[order(bb[,fill]),] # genus to fill
  p = ggplot(bb, aes_string(x = x, y = y,
                            fill = fill),
             environment = .e, ordered = FALSE)


  p = p +geom_bar(stat = "identity",
                  position = "stack",
                  color = "black")

  p = p + theme(axis.text.x = element_text(angle = -90, hjust = 0))

  p = p + guides(fill = guide_legend(override.aes = list(colour = NULL), reverse=TRUE)) +
    theme(legend.key = element_rect(colour = "black"))
```

```
  p = p + theme(legend.key.size = unit(leg_size, "cm"))


  if (!is.null(title)) {
    p <- p + ggtitle(title)
  }
  return(p)
}

# END #
```

Plot phylum

Choosing the cutoff at all seqs above 0.01% abundance. This leaves us with four phyla and is visually informative. Then plotting the phylum. This is study dependent, so try different abundance cutoffs.

```
#Make a list of sample names for plotting
increaseAs = c("DahanA1","DahanA3","DahanA4","DahanA5","DahanA6","DahanA7","DahanA8","DahanA9","DahanA1[illegible]

#Filtering taxa <0.01% abundance
psFRphy = filter_taxa(psFR, function(x) mean(x) > 0.001, TRUE)

#Plotting
phyplot = plot_ordered_bar(psFRphy, fill = "Phylum") +
  scale_x_discrete(limits=increaseAs) + theme_classic() +
  theme(axis.text.x = element_text(angle = 90, hjust = 1))

phyplot
```

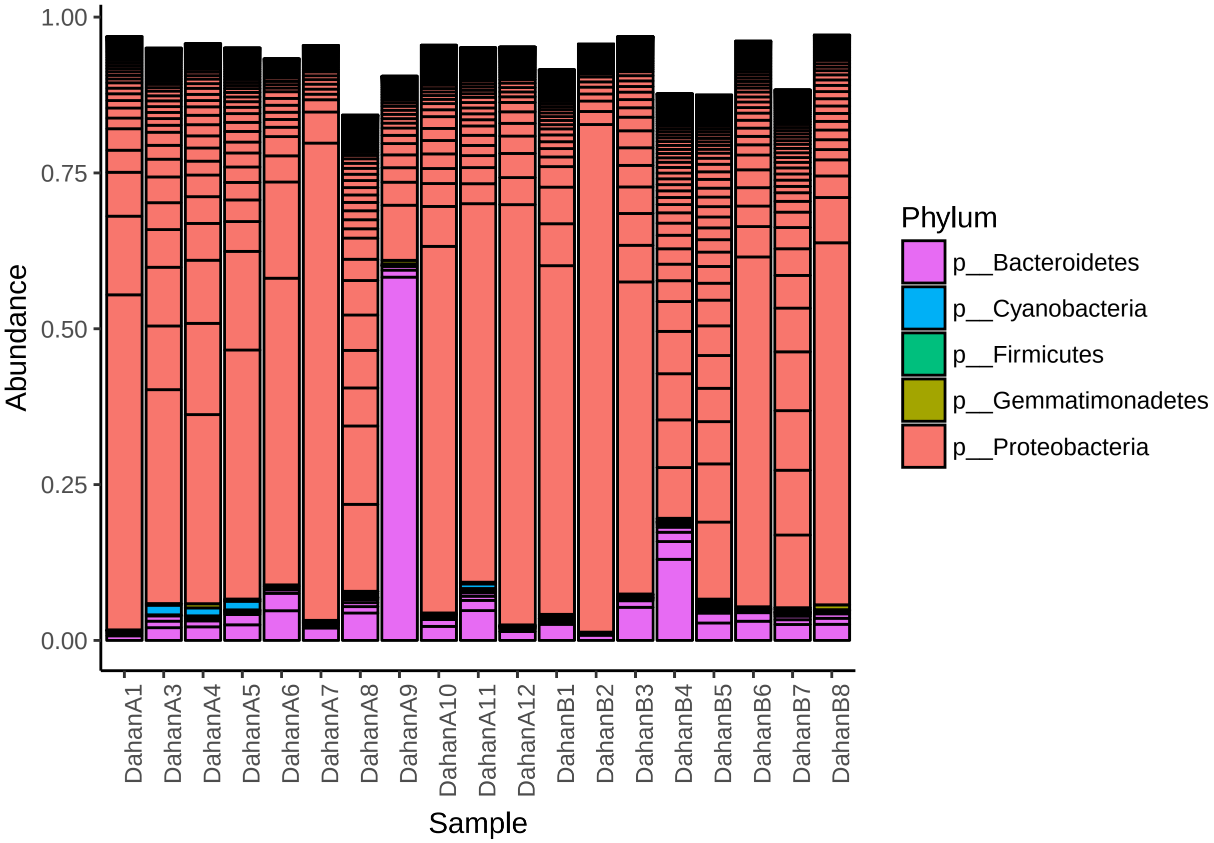

Plot with faceting rather than stacking

```
phyplot_facet = plot_ordered_bar(psFRphy, fill = "Phylum") +
  scale_x_discrete(limits=increaseAs) +
  facet_wrap(~Phylum,4) + theme_bw() +
  theme(axis.text.x = element_text(angle = 90, hjust = 1))

phyplot_facet
```

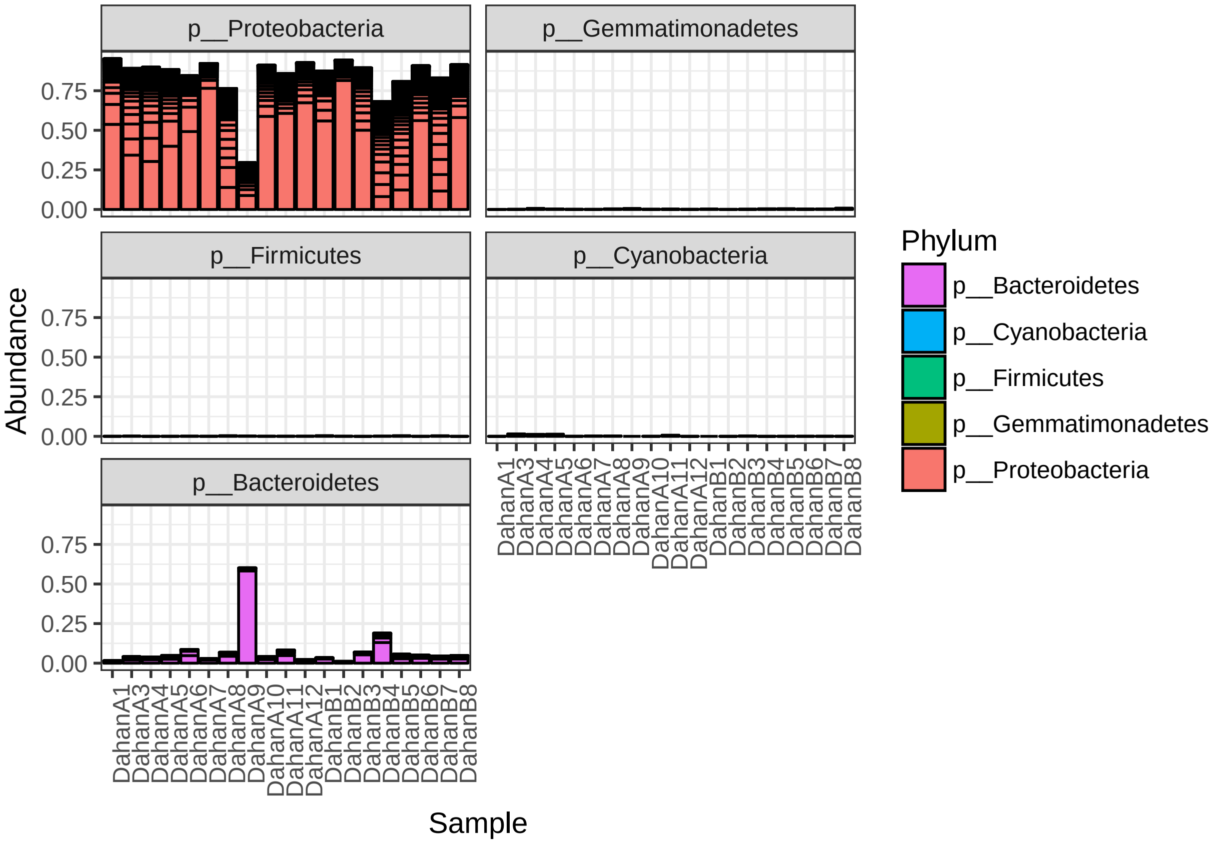

Plot family

Choosing the cutoff of all seqs above 0.30% abundance because this leaves a visually informative amount of taxa. Then plotting families. Again, analysis dependent so choose an appropriate cutoff.

```
psFRFam = filter_taxa(psFR, function(x) mean(x) > 0.003, TRUE)

famplot <- plot_ordered_bar(psFRFam, fill = "Family") +
  scale_x_discrete(limits=increaseAs) + theme_classic() +
  theme(axis.text.x = element_text(angle = 90, hjust = 1)) + ylim(0,1)


famplot
```

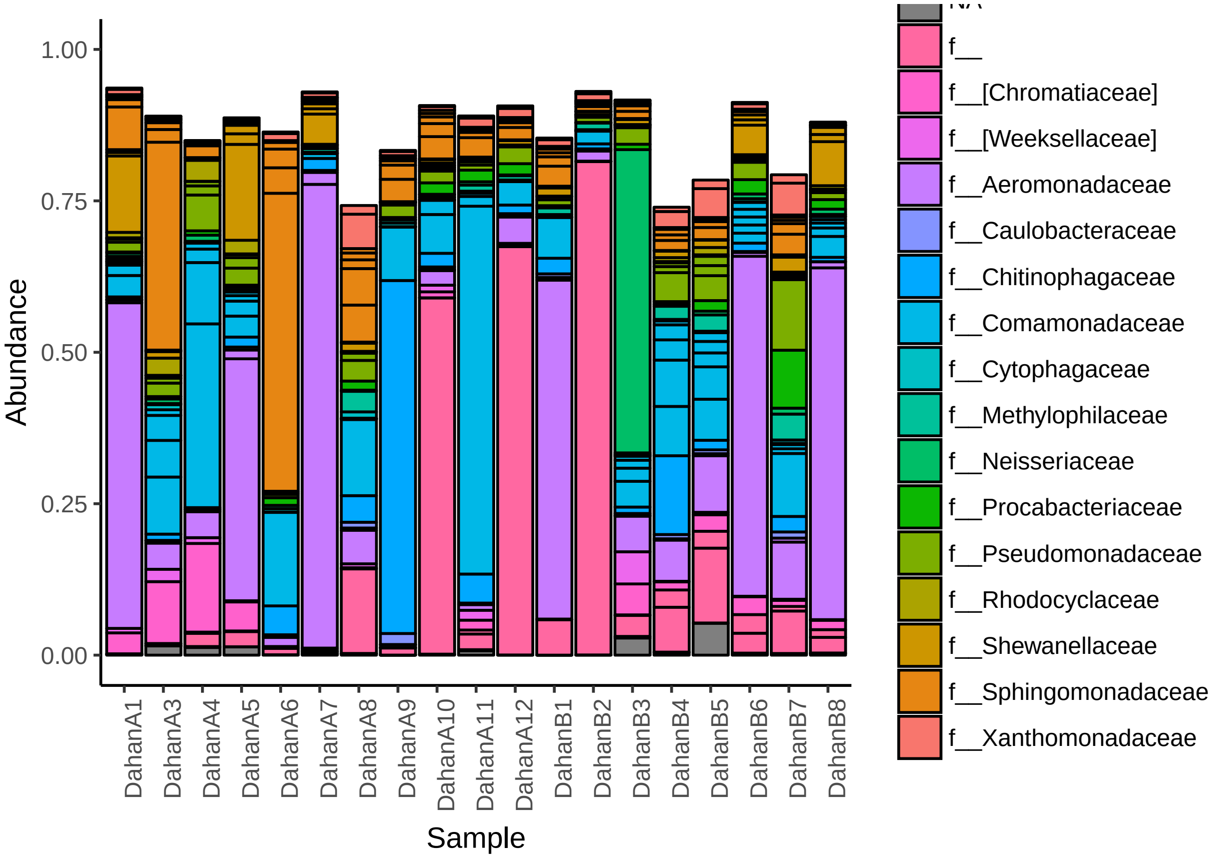

Plot with faceting rather than stacking

```
famplot_facet = plot_ordered_bar(psFRFam, fill = "Family") +
  scale_x_discrete(limits=increaseAs) +
  facet_wrap(~Family,16) + theme_bw() +
  theme(axis.text.x = element_text(angle = 90, hjust = 1)) + ylim(0,1)

famplot_facet
```

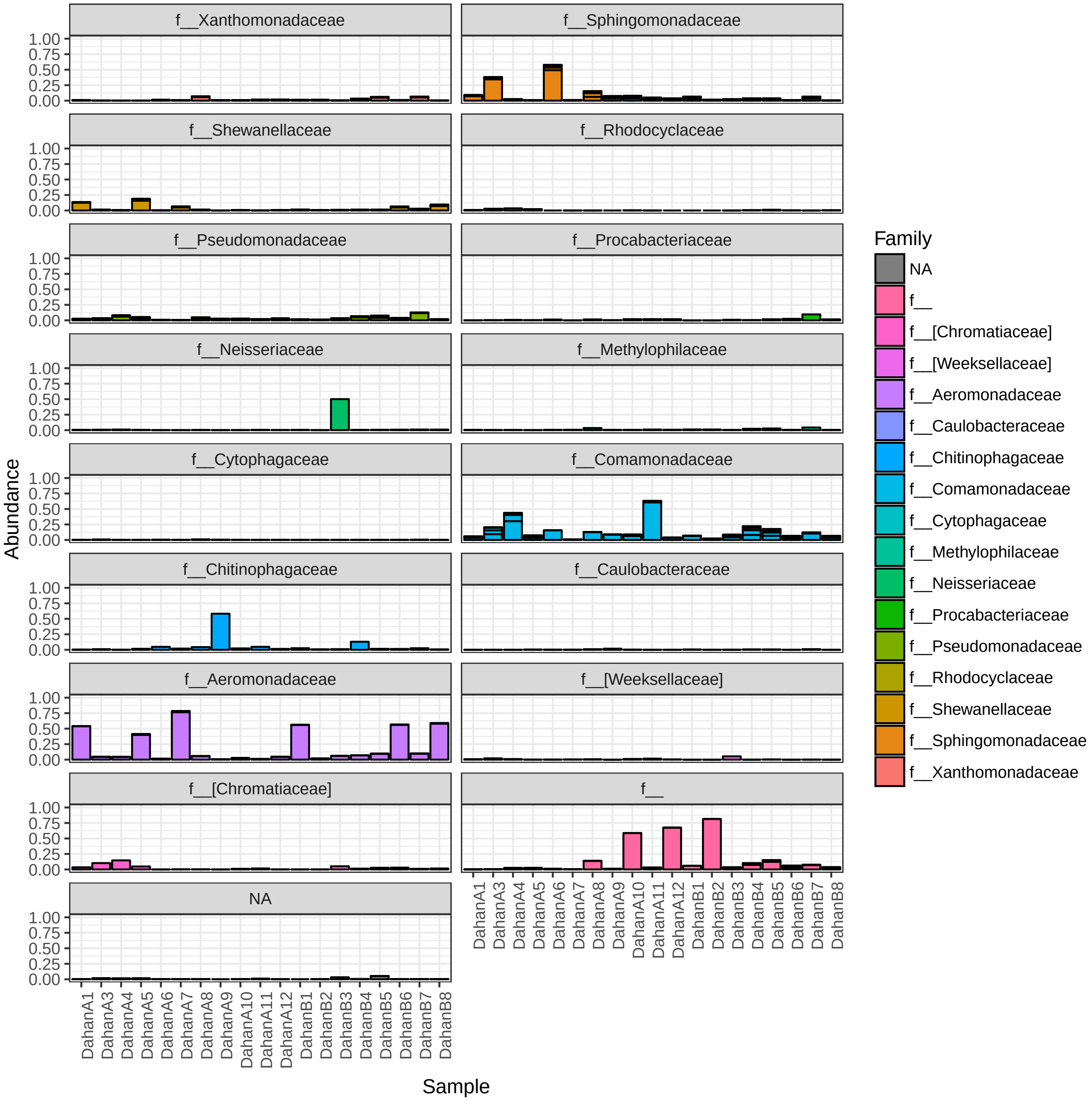

Core microbiome

Here we investigate the control core microbiome by looking at RSV found in every sample of the control. First we subset our phyloseq object to only contain these samples, then to only contain those RSVs with a sum > 0 in 99% of samples (or all four in this case).

We then create a taxa summary using the prototype function `summarize_taxa`, which can be found on the phyloseq github forum.

```
#Make phyloseq object w/ previously saved sequence table, metadata and taxa table

#Load sequence table, taxonomy and metadata from DADA2 processing. This does not work when we have a ph
seqtab.nochim.sq <- readRDS('~/Documents/QIIME_fish/forward_read_analysis/bowtie_dada2_phylo/R1_nophix_
```

```
taxa.sq <- readRDS('~/Documents/QIIME_fish/forward_read_analysis/bowtie_dada2_phylo/R1_nophix_split_by_s
metad <- readRDS('~/Documents/QIIME_fish/forward_read_analysis/bowtie_dada2_phylo/R1_nophix_split_by_sam


psCm <- phyloseq(otu_table(seqtab.nochim.sq, taxa_are_rows=FALSE),
                 sample_data(metad),
                 tax_table(taxa.sq))

psCm <- prune_taxa(myTaxa,psCm)

#Make list of control sample names
ctrl.samps <- levels(factor(metad[which(metad$Treatment == "Control"),][,"SampleID"]))
#Remove sample that failed sequencing
ctrl.samps <- ctrl.samps[-2]


#Subset phyloseq object to only contain these samps
psCmC <- subset_samples(psCm,SampleID == ctrl.samps)

#Filter ISeVs (taxa) to only those with abun > 0 and found in every sample
psCmC.core <- filter_taxa(psCmC, function(x) sum(x>0) > (0.99*length(x)), TRUE)
```

Here we see 122 taxa are part of the 99% core microbiome.

```
psCmC.core
```

```
## phyloseq-class experiment-level object
## otu_table()   OTU Table:         [ 120 taxa and 4 samples ]
## sample_data() Sample Data:       [ 4 samples by 8 sample variables ]
## tax_table()   Taxonomy Table:    [ 120 taxa by 7 taxonomic ranks ]
```

```
#Write this out to have a table of core microbes
write.csv(tax_table(psCmC.core),'~/Documents/QIIME_fish/forward_read_analysis/bowtie_dada2_phylo/R1_nopl
```

Here we use phyloseq's prototype functions `fast_melt` and `summarize_taxa` to summarize proportion each phylum in the core microbiome. Again, these can be found on the phyloseq github forum

```
fast_melt = function(physeq){
# supports "naked" otu_table as `physeq` input.
otutab = as(otu_table(physeq), "matrix")
if(!taxa_are_rows(physeq)){otutab <- t(otutab)}
otudt = data.table(otutab, keep.rownames = TRUE)
setnames(otudt, "rn", "taxaID")
# Enforce character taxaID key
otudt[, taxaIDchar := as.character(taxaID)]
otudt[, taxaID := NULL]
setnames(otudt, "taxaIDchar", "taxaID")
# Melt count table
mdt = melt.data.table(otudt,
id.vars = "taxaID",
variable.name = "SampleID",
value.name = "count")
# Remove zeroes, NAs
mdt <- mdt[count > 0][!is.na(count)]
# Calculate relative abundance
mdt[, RelativeAbundance := count / sum(count), by = SampleID]
```

```
if(!is.null(tax_table(physeq, errorIfNULL = FALSE))){
# If there is a tax_table, join with it. Otherwise, skip this join.
taxdt = data.table(as(tax_table(physeq, errorIfNULL = TRUE), "matrix"), keep.rownames = TRUE)
setnames(taxdt, "rn", "taxaID")
# Enforce character taxaID key
taxdt[, taxaIDchar := as.character(taxaID)]
taxdt[, taxaID := NULL]
setnames(taxdt, "taxaIDchar", "taxaID")
# Join with tax table
setkey(taxdt, "taxaID")
setkey(mdt, "taxaID")
mdt <- taxdt[mdt]
}
return(mdt)
}

se <- function(x) sqrt(var(x)/length(x))

summarize_taxa = function(physeq, Rank, GroupBy = NULL){
Rank <- Rank[1]
if(!Rank %in% rank_names(physeq)){
message("The argument to `Rank` was:\n", Rank,
"\nBut it was not found among taxonomic ranks:\n",
paste0(rank_names(physeq), collapse = ", "), "\n",
"Please check the list shown above and try again.")
}
if(!is.null(GroupBy)){
GroupBy <- GroupBy[1]
if(!GroupBy %in% sample_variables(physeq)){
message("The argument to `GroupBy` was:\n", GroupBy,
"\nBut it was not found among sample variables:\n",
paste0(sample_variables(physeq), collapse = ", "), "\n",
"Please check the list shown above and try again.")
}
}
# Start with fast melt
mdt = fast_melt(physeq)
if(!is.null(GroupBy)){
# Add the variable indicated in `GroupBy`, if provided.
sdt = data.table(SampleID = sample_names(physeq),
var1 = get_variable(physeq, GroupBy))
setnames(sdt, "var1", GroupBy)
# Join
setkey(sdt, SampleID)
setkey(mdt, SampleID)
mdt <- sdt[mdt]
}
# Summarize
Nsamples = nsamples(physeq)
summarydt = mdt[, list(meanRA = sum(RelativeAbundance)/Nsamples,
sdRA = sd(RelativeAbundance),
seRA = sd(RelativeAbundance)/sqrt(nsamples(physeq)),
seRA = se(RelativeAbundance),
```

```r
minRA = min(RelativeAbundance),
maxRA = max(RelativeAbundance)),
by = c(Rank, GroupBy)]
return(summarydt)
}


#Summarize taxa. Here we see what proportion of the overall core microbiome each of these phyla constit
summarize_taxa(psCmC.core, "Phylum")
```

```
##                  Phylum       meanRA         sdRA         seRA
##  1:   p__Proteobacteria 0.9413605099 0.0459000466 2.295002e-02
##  2:    p__Bacteroidetes 0.0416719145 0.0059801598 2.990080e-03
##  3:   p__Planctomycetes 0.0001456640 0.0001090513 5.452563e-05
##  4:       p__Firmicutes 0.0011767395 0.0004090226 2.045113e-04
##  5:              p__TM6 0.0001223926 0.0001180259 5.901295e-05
##  6:   p__Actinobacteria 0.0002150724 0.0001032765 5.163825e-05
##  7:                  NA 0.0013386149 0.0008536702 4.268351e-04
##  8:  p__Verrucomicrobia 0.0003604918 0.0002045140 1.022570e-04
##  9:    p__Cyanobacteria 0.0106761579 0.0070538181 3.526909e-03
## 10: p__Gemmatimonadetes 0.0029324425 0.0023339328 1.166966e-03
##             seRA        minRA        maxRA
##  1: 2.392705e-03 8.753425e-06 0.5437553336
##  2: 8.292990e-04 2.188356e-05 0.0255718245
##  3: 5.452563e-05 5.252055e-05 0.0003033186
##  4: 1.022556e-04 5.247271e-05 0.0017308122
##  5: 5.901295e-05 2.626027e-05 0.0002860846
##  6: 3.651376e-05 2.209377e-05 0.0003343398
##  7: 3.018180e-04 1.657033e-05 0.0017813219
##  8: 7.230662e-05 1.657033e-05 0.0006258699
##  9: 3.526909e-03 2.375080e-04 0.0156405866
## 10: 8.251699e-04 5.252055e-05 0.0068845686
```

Alpha diversity analyses

We are running all diveristy and abundance analyses on subsampled reads, since we are using observed species as part of our metrics. Using our DADA2 sequences this is acceptable and highly advised. However, when looking at differential abundance we will not subsample since we can use a Negative Binomial to accurately capture our distribution, for more information see Waste Not, Want Not: Why Rarefying Microbiome Data is Inadmissable, McMurdie and Holmes 2014

Calculate alpha diversity

First run `estimate_richness` on the phyloseq object. We can ignore the warnings since we processed our reads with DADA2, which relies on multiple observation of reads to correct sequences and so does not output singletons, and the warning is in regards to diversity metrics (e.g., Chao1) that require singletons. Singletons are likely just sequencing errors and so were removed and since chao1 relies on singleton presence these methods do not mix well..

Additionally, we are conducting alpha diversity analyses on our unfiltered table to allow rare species and fine-scale differences between samples to be accounted for.

```
psrar = rarefy_even_depth(pspy,sample.size = min(sample_sums(pspy)))
```

```
## You set `rngseed` to FALSE. Make sure you've set & recorded
##  the random seed of your session for reproducibility.
## See `?set.seed`

## ...

## 274OTUs were removed because they are no longer
## present in any sample after random subsampling

## ...
```

```
sample_sums(psrar)
```

```
##  DahanA1 DahanA10 DahanA11 DahanA12  DahanA3  DahanA4  DahanA5  DahanA6
##   119609   119609   119609   119609   119609   119609   119609   119609
##  DahanA7  DahanA8  DahanA9  DahanB1  DahanB2  DahanB3  DahanB4  DahanB5
##   119609   119609   119609   119609   119609   119609   119609   119609
##  DahanB6  DahanB7  DahanB8
##   119609   119609   119609
```

```
alph <- estimate_richness(psrar)
```

Then add metadata to the alpha richness table.

```
#Make a dataframe from metadata
metadt <- as.data.frame(sample_data(pspy))

#Add treatment and asconc (categorical and continuous) arsenic concentrations to alpha div table
alph["Treatment"] = metadt[,"Treatment"]
alph["AsConc"] = metadt[,"AsConc"]



#Save this out for making tables.
setwd("/Users//Dylan/Documents/QIIME_fish/forward_read_analysis/bowtie_dada2_phylo/R1_nophix_split_by_sa
write.csv(alph,"~/Documents/QIIME_fish/forward_read_analysis/bowtie_dada2_phylo/R1_nophix_split_by_sampl
```

Make models and plot alpha diversity

Next plot the diversity metrics according to metadata categories and run linear and polynomial regressions. Here I plot the Shannon (richness and eveness) index, observed species and Pielou's evennes indices. You can plot any you'd like but again be careful with those that rely on singletons. We chose to use the sequence table with no preprocessing of lowly abundant RSVs, since rare species can play a large role in defining a samples' alpha diversity.

Observed species

Here we are plotting Observed species, which in this case is technically observed RSVs.

```
obs <- ggplot(alph, aes(Treatment,Observed)) +
geom_boxplot(aes(fill = Treatment))  +
scale_x_discrete(limits =c("Control", "10ppb", "50ppb", "100ppb")) +
ylab("Observed species") +
```

```
xlab(" ") + theme_classic() +
stat_summary(fun.y=mean, geom = "point", shape = 5, size = 4) + scale_fill_manual(values=c("#D55E00","#
```

```
obs
```

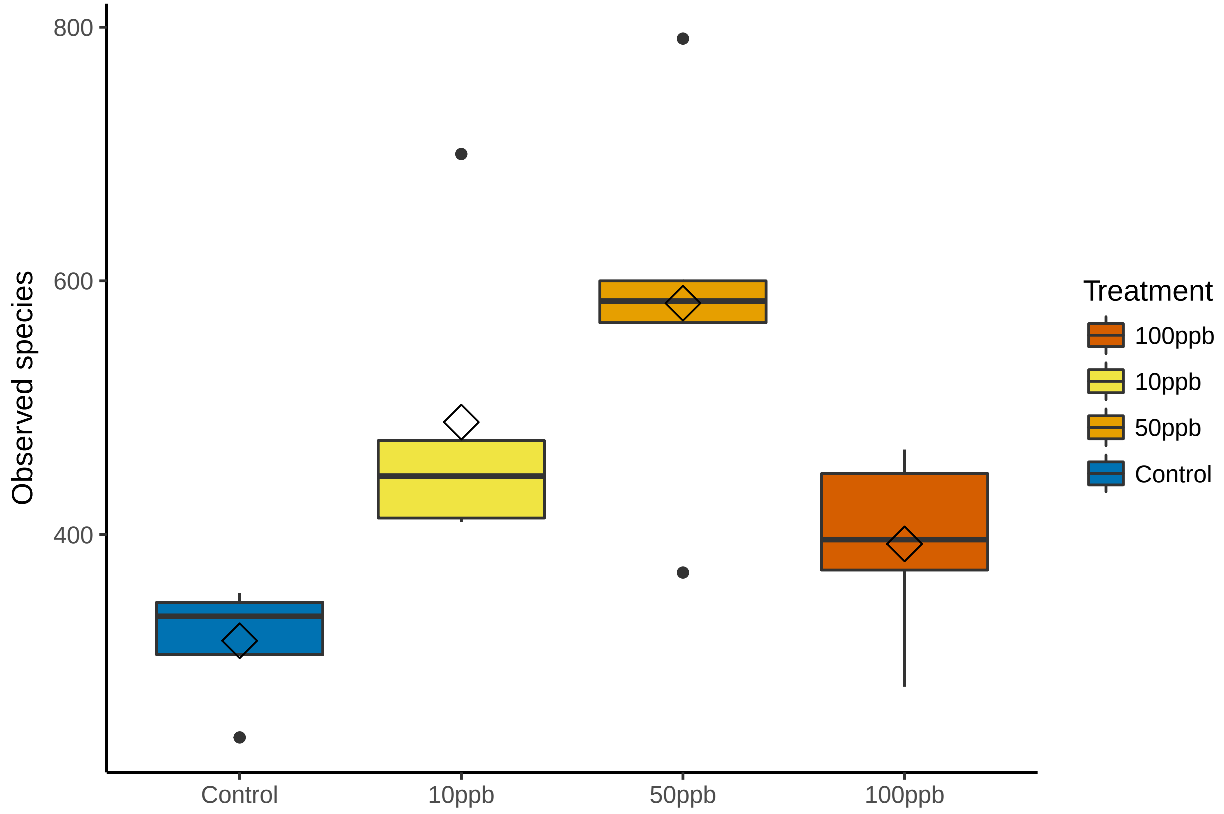

Some of those values look like outliers. We can use the package outliers to investigate this. Let's look if the outlying values in the 10 and 50ppb groups can be identified as statistical outliers..

```
library(outliers)
```

```
##
## Attaching package: 'outliers'

## The following object is masked from 'package:vegan':
##
##     scores
```

```
tenout = subset(alph, Treatment == "10ppb")
dixon.test(tenout$Observed)
```

```
##
##  Dixon test for outliers
##
## data:  tenout$Observed
## Q = 0.77931, p-value = 0.02021
## alternative hypothesis: highest value 700 is an outlier
```

It looks so! We now remove this sample for the diversity measurement.

```
alph = alph[-10,]
```

For the value at 50ppb…

```
fifout = subset(alph, Treatment == "50ppb")
dixon.test(fifout$Observed)
```

```
##
##  Dixon test for outliers
##
## data:  fifout$Observed
## Q = 0.46793, p-value = 0.3625
## alternative hypothesis: lowest value 370 is an outlier
```

Here we see the lowest value at 50ppb not an outlier.

Let's replot w/ out our outlier..

```
obs <- ggplot(alph, aes(Treatment,Observed)) +
geom_boxplot(aes(fill = Treatment))  +
scale_x_discrete(limits =c("Control", "10ppb", "50ppb", "100ppb")) +
ylab("Observed species") +
xlab(" ") + theme_classic() +
stat_summary(fun.y=mean, geom = "point", shape = 5, size = 4) + scale_fill_manual(values=c("#D55E00","#I


obs
```

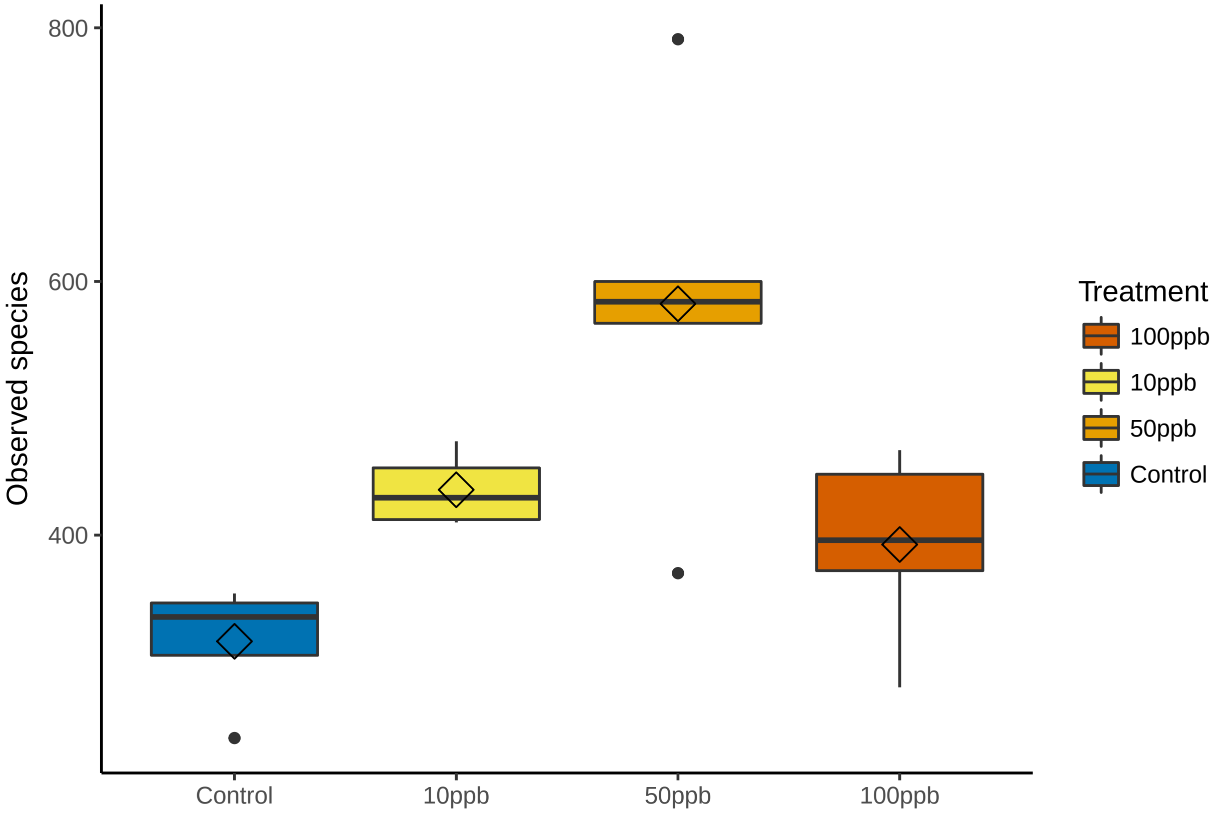

Let's go ahead and make and summarize our models…

```r
model01<- lm(log(Observed) ~ AsConc,data=alph)
summary(model01)
```

```
##
## Call:
## lm(formula = log(Observed) ~ AsConc, data = alph)
##
## Residuals:
##     Min      1Q  Median      3Q     Max
## -0.5105 -0.1469  0.0052  0.1431  0.6239
##
## Coefficients:
##             Estimate Std. Error t value Pr(>|t|)
## (Intercept) 5.991181   0.102818   58.27   <2e-16 ***
## AsConc      0.001165   0.001739    0.67    0.512
## ---
## Signif. codes:  0 '***' 0.001 '**' 0.01 '*' 0.05 '.' 0.1 ' ' 1
##
## Residual standard error: 0.2922 on 16 degrees of freedom
## Multiple R-squared:  0.02729,    Adjusted R-squared:  -0.0335
## F-statistic: 0.4489 on 1 and 16 DF,  p-value: 0.5124
```

```r
model02 <- lm(log(Observed) ~ AsConc + I(AsConc^2),data=alph)
summary(model02)
```

```
##
## Call:
## lm(formula = log(Observed) ~ AsConc + I(AsConc^2), data = alph)
##
## Residuals:
##      Min       1Q   Median       3Q      Max
## -0.44891 -0.02009  0.02978  0.09655  0.31089
##
## Coefficients:
##               Estimate Std. Error t value Pr(>|t|)
## (Intercept)  5.804e+00  8.358e-02  69.443  < 2e-16 ***
## AsConc       2.085e-02  4.755e-03   4.385 0.000532 ***
## I(AsConc^2) -1.937e-04  4.526e-05  -4.280 0.000658 ***
## ---
## Signif. codes:  0 '***' 0.001 '**' 0.01 '*' 0.05 '.' 0.1 ' ' 1
##
## Residual standard error: 0.2025 on 15 degrees of freedom
## Multiple R-squared:  0.5621, Adjusted R-squared:  0.5037
## F-statistic: 9.627 on 2 and 15 DF,  p-value: 0.002043
```

```r
model03 <- lm(log(Observed) ~ AsConc + I(AsConc^2) + I(AsConc^3),data=alph)
summary(model03)
```

```
##
## Call:
## lm(formula = log(Observed) ~ AsConc + I(AsConc^2) + I(AsConc^3),
##     data = alph)
##
## Residuals:
##      Min       1Q   Median       3Q      Max
```

```
## -0.42530 -0.04846  0.02807  0.09311  0.33450
##
## Coefficients:
##               Estimate Std. Error t value Pr(>|t|)
## (Intercept)  5.745e+00  1.010e-01  56.877   <2e-16 ***
## AsConc       4.015e-02  1.924e-02   2.087   0.0556 .
## I(AsConc^2) -7.509e-04  5.400e-04  -1.390   0.1861
## I(AsConc^3)  3.706e-06  3.579e-06   1.035   0.3181
## ---
## Signif. codes:  0 '***' 0.001 '**' 0.01 '*' 0.05 '.' 0.1 ' ' 1
##
## Residual standard error: 0.202 on 14 degrees of freedom
## Multiple R-squared:  0.5932, Adjusted R-squared:  0.5061
## F-statistic: 6.806 on 3 and 14 DF,  p-value: 0.004643
```

Let's plot the models to see how well the fit the data.

```r
plot(alph$AsConc, log(alph$Observed), type="p", lwd=3)
points(alph$AsConc,predict(model01), type="l", col="green",lwd=2)
points(alph$AsConc, predict(model02), type = "l", col="red",lwd=2)
points(alph$AsConc, predict(model03), type="l", col="blue", lwd=2)
```

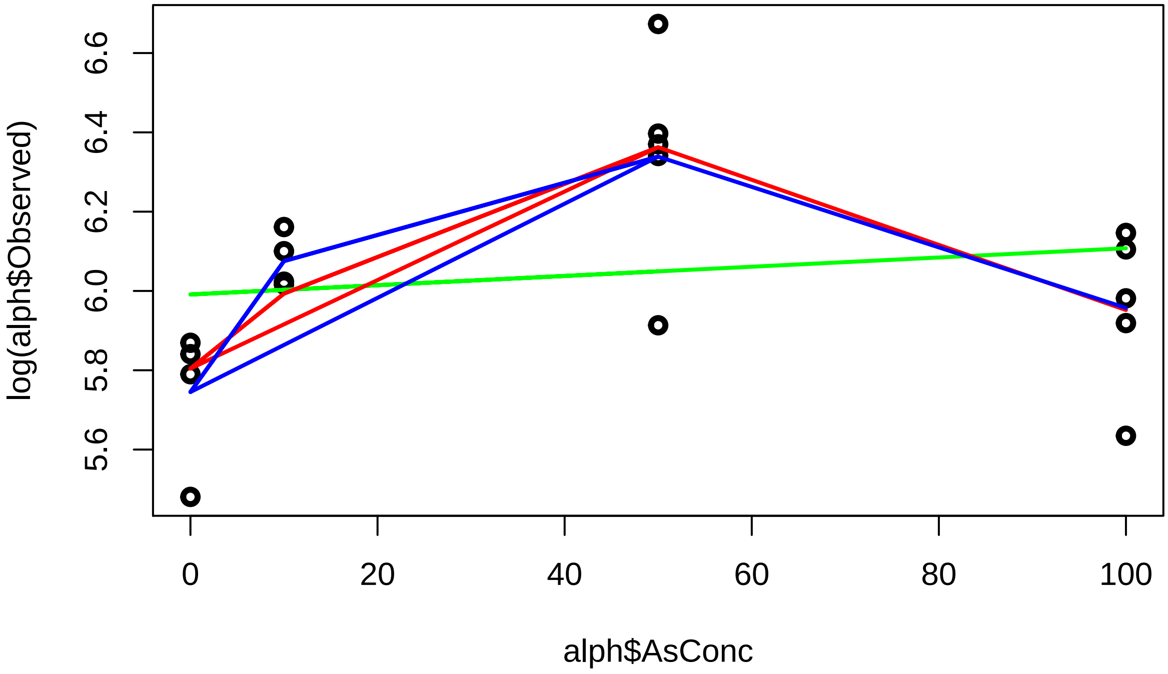

What model is the best to use? One simple metric to use is Akaike information criterion (AIC).A lower AIC value means the model is preferred. The AIC deals with the tradeoff between the goodness of fit and parsimony. More details can be found in Michael J. Crawley's book on stats in R. Of course, being that it is a quick and dirty tool, there are proponents who rightfully advocate heedfulness.

```r
AIC(model01,model02,model03)
```

```
##         df       AIC
## model01  3 10.673598
## model02  4 -1.692000
## model03  5 -1.019875
```

As we see, the second model offers the lowest AIC value, and therefore explains the data best while retaining

a reasonable amount of parsimony.

Shannon metric

Plotting Shannon diversity. With median and hinges as 25th and 75th quartiles and means as diamonds.

```
shan <- ggplot(alph, aes(Treatment,Shannon)) +
  geom_boxplot(aes(fill = Treatment)) +
  scale_x_discrete(limits =c("Control", "10ppb", "50ppb", "100ppb")) +
  stat_summary(fun.y=mean, geom = "point", shape = 5, size = 4) +
  scale_fill_manual(values=c("#D55E00","#F0E442", "#E69F00", "#0072B2"))  +
  theme_classic()

shan
```

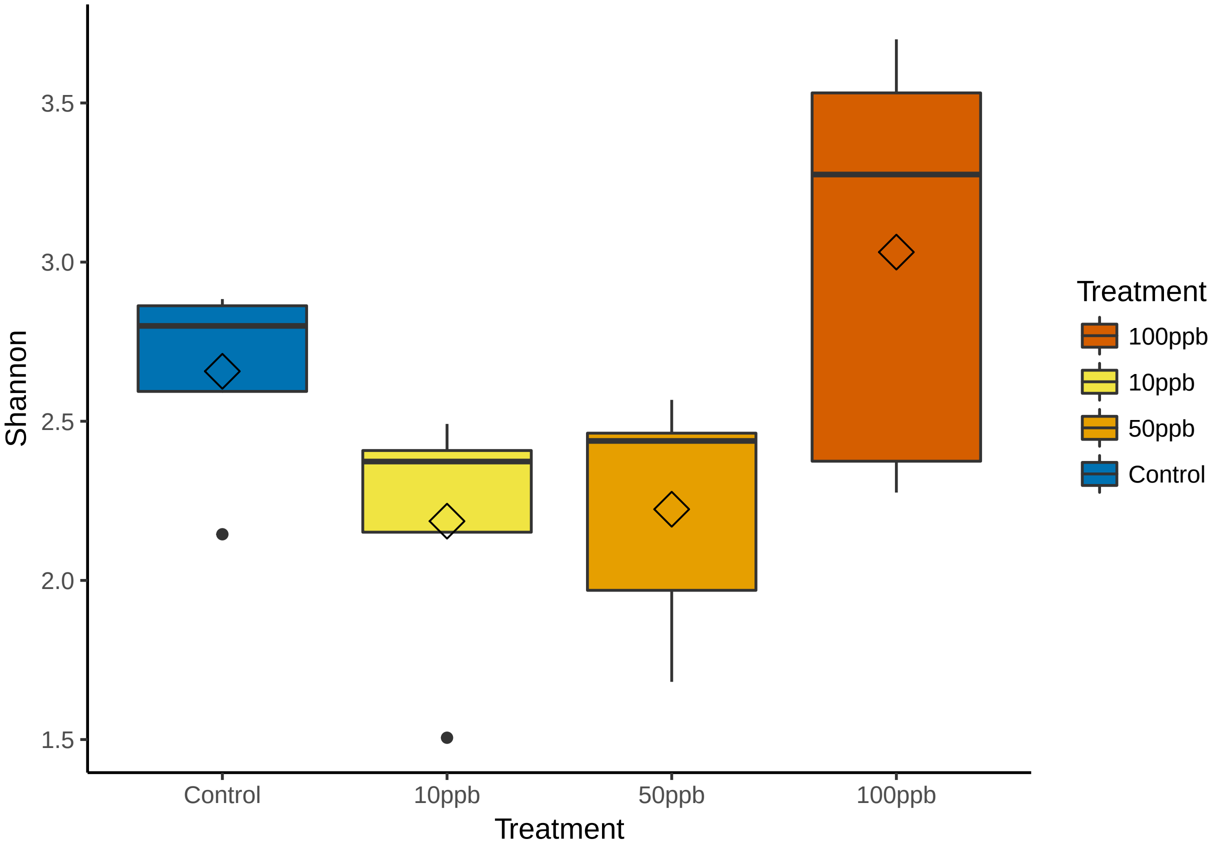

Making and showing anova tables of models, using Shannon diversity as our response and arsenic concentration as our explanatory.

```
models1 <- lm(Shannon ~ AsConc,data=alph)
summary(models1)
```

```
##
## Call:
## lm(formula = Shannon ~ AsConc, data = alph)
##
## Residuals:
##      Min       1Q   Median       3Q      Max
## -0.88842 -0.39039  0.00755  0.44448  0.85459
```

```
##
## Coefficients:
##             Estimate Std. Error t value Pr(>|t|)
## (Intercept) 2.293965   0.191339  11.989 2.08e-09 ***
## AsConc      0.005514   0.003237   1.704    0.108
## ---
## Signif. codes:  0 '***' 0.001 '**' 0.01 '*' 0.05 '.' 0.1 ' ' 1
##
## Residual standard error: 0.5438 on 16 degrees of freedom
## Multiple R-squared:  0.1535, Adjusted R-squared:  0.1006
## F-statistic: 2.902 on 1 and 16 DF,  p-value: 0.1078
```

```
models2 <-lm(Shannon ~ AsConc + I(AsConc^2),data=alph)
summary(models2)
```

```
##
## Call:
## lm(formula = Shannon ~ AsConc + I(AsConc^2), data = alph)
##
## Residuals:
##     Min      1Q  Median      3Q     Max
## -0.8555 -0.3406  0.1714  0.3160  0.6573
##
## Coefficients:
##               Estimate Std. Error t value Pr(>|t|)
## (Intercept)  2.5309152  0.2001090  12.648  2.1e-09 ***
## AsConc      -0.0194279  0.0113838  -1.707   0.1085
## I(AsConc^2)  0.0002455  0.0001084   2.265   0.0388 *
## ---
## Signif. codes:  0 '***' 0.001 '**' 0.01 '*' 0.05 '.' 0.1 ' ' 1
##
## Residual standard error: 0.4848 on 15 degrees of freedom
## Multiple R-squared:  0.3692, Adjusted R-squared:  0.2851
## F-statistic: 4.391 on 2 and 15 DF,  p-value: 0.03155
```

```
models3  <-lm(Shannon ~ AsConc + I(AsConc^2) + I(AsConc^3),data=alph)
summary(models3)
```

```
##
## Call:
## lm(formula = Shannon ~ AsConc + I(AsConc^2) + I(AsConc^3), data = alph)
##
## Residuals:
##     Min      1Q  Median      3Q     Max
## -0.7555 -0.4477  0.1965  0.2426  0.6685
##
## Coefficients:
##               Estimate Std. Error t value Pr(>|t|)
## (Intercept)  2.657e+00  2.437e-01  10.902 3.17e-08 ***
## AsConc      -6.066e-02  4.642e-02  -1.307    0.212
## I(AsConc^2)  1.436e-03  1.303e-03   1.102    0.289
## I(AsConc^3) -7.915e-06  8.636e-06  -0.917    0.375
## ---
## Signif. codes:  0 '***' 0.001 '**' 0.01 '*' 0.05 '.' 0.1 ' ' 1
##
```

```
## Residual standard error: 0.4874 on 14 degrees of freedom
## Multiple R-squared:  0.405,  Adjusted R-squared:  0.2774
## F-statistic: 3.176 on 3 and 14 DF,  p-value: 0.05734
```

Again, we can plot the models against the data and see how well they fit.

```
plot(alph$AsConc, alph$Shannon, type="p", lwd=3)
points(alph$AsConc,predict(models1), type="l", col="green",lwd=2)
points(alph$AsConc, predict(models2), type = "l", col="red",lwd=2)
points(alph$AsConc, predict(models3), type="l", col="blue", lwd=2)
```

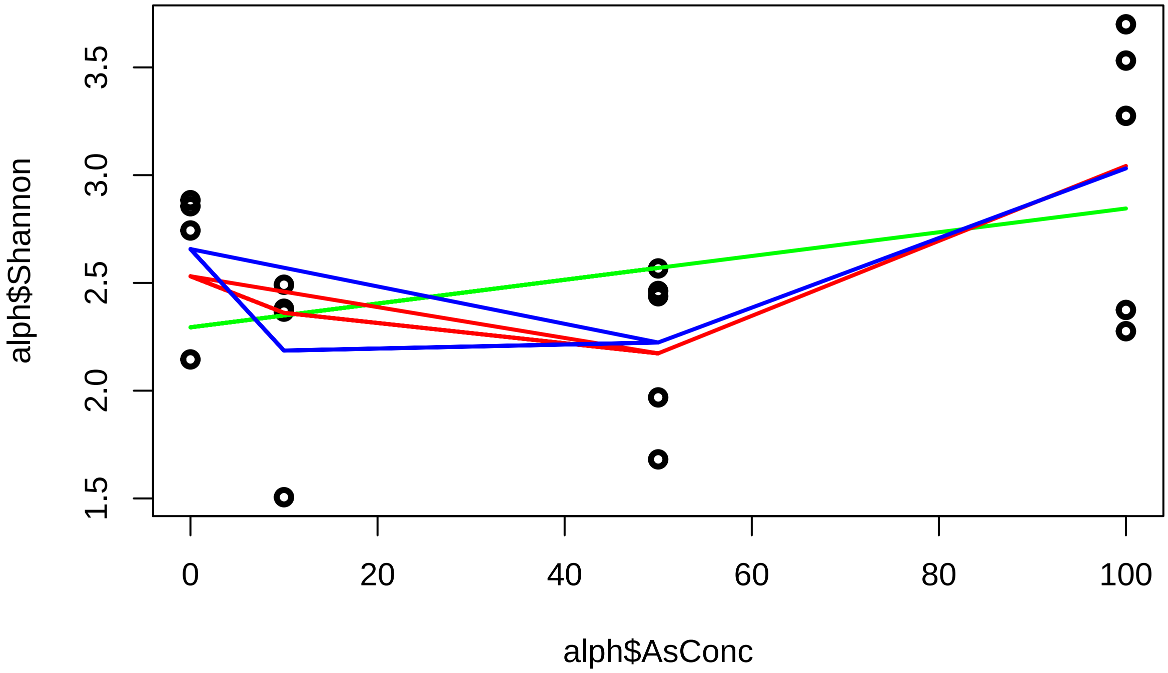

Here we see our first model, the linear regression, explains the data best.

```
AIC(models1,models2,models3)
```

```
##         df      AIC
## models1  3 33.03260
## models2  4 29.73762
## models3  5 30.68875
```

As we see, the second model offers the lowest AIC value.

Pielou's evenness

Plotting Pielou's evenness measurement. We are doing this because Shannon diversity considers both observations and ecosystem equality, and since the observed species trend was rather different than our Shannon diversity trend, it seems apparant that the Shannon trend was due to evenness. This metric is not part of the standard output of Phyloseq's but we can easily calculate it with the simple equaton developed by Pielou 1966. It's a throwback!

```
#Calculate Pielou's Evenness Metric
#Where Pielou's evenness metric is simply J' = H'/H'max
#Where H' = the Shannon index and H'max = logS, where S is species number or observed ISeVs in this cas
Pielou = (alph$Shannon/log(alph$Observed))
alph["Pielou"] = Pielou
```

```
piel <- ggplot(alph, aes(Treatment,Pielou)) +
  geom_boxplot(aes(fill = Treatment)) +
  scale_x_discrete(limits =c("Control", "10ppb", "50ppb", "100ppb")) +
  ylab("Pielou's evenness") +
  theme_classic()+
  stat_summary(fun.y=mean, geom = "point", shape = 5, size = 4) + scale_fill_manual(values=c("#D55E00",


piel
```

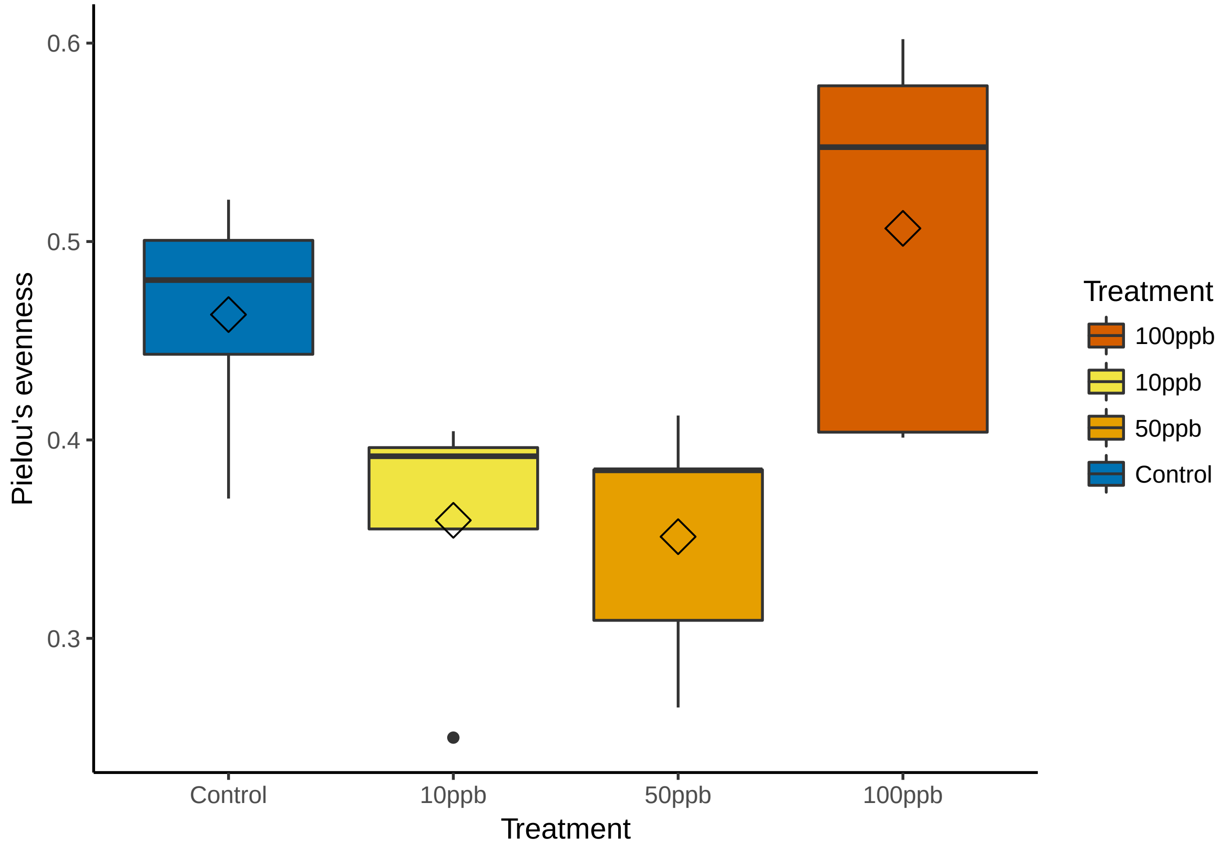

Making models with Pielou's evenness…

```
modelp1 <- lm(Pielou ~ AsConc,data=alph)
summary(modelp1)
```

```
##
## Call:
## lm(formula = Pielou ~ AsConc, data = alph)
##
## Residuals:
##       Min        1Q    Median        3Q       Max
## -0.160885 -0.057269 -0.008637  0.081606  0.135496
##
## Coefficients:
##              Estimate Std. Error t value Pr(>|t|)
## (Intercept) 0.3856210  0.0336746  11.451 4.04e-09 ***
## AsConc      0.0008086  0.0005697   1.419    0.175
```

```
## ---
## Signif. codes:  0 '***' 0.001 '**' 0.01 '*' 0.05 '.' 0.1 ' ' 1
##
## Residual standard error: 0.09571 on 16 degrees of freedom
## Multiple R-squared:  0.1118, Adjusted R-squared:  0.05633
## F-statistic: 2.015 on 1 and 16 DF,  p-value: 0.175
```

```
modelp2 <-lm(Pielou ~ AsConc + I(AsConc^2),data=alph)
summary(modelp2)
```

```
##
## Call:
## lm(formula = Pielou ~ AsConc + I(AsConc^2), data = alph)
##
## Residuals:
##      Min       1Q   Median       3Q      Max
## -0.14634 -0.05754  0.01942  0.05391  0.09300
##
## Coefficients:
##               Estimate Std. Error t value Pr(>|t|)
## (Intercept)  4.367e-01  3.208e-02  13.613 7.58e-10 ***
## AsConc      -4.564e-03  1.825e-03  -2.501  0.02445 *
## I(AsConc^2)  5.287e-05  1.737e-05   3.044  0.00821 **
## ---
## Signif. codes:  0 '***' 0.001 '**' 0.01 '*' 0.05 '.' 0.1 ' ' 1
##
## Residual standard error: 0.07772 on 15 degrees of freedom
## Multiple R-squared:  0.451,  Adjusted R-squared:  0.3777
## F-statistic:  6.16 on 2 and 15 DF,  p-value: 0.01115
```

```
modelp3  <-lm(Pielou ~ AsConc + I(AsConc^2) + I(AsConc^3),data=alph)
summary(modelp3)
```

```
##
## Call:
## lm(formula = Pielou ~ AsConc + I(AsConc^2) + I(AsConc^3), data = alph)
##
## Residuals:
##      Min       1Q   Median       3Q      Max
## -0.10952 -0.07510  0.03208  0.04392  0.09535
##
## Coefficients:
##               Estimate Std. Error t value Pr(>|t|)
## (Intercept)  4.632e-01  3.821e-02  12.120 8.23e-09 ***
## AsConc      -1.323e-02  7.278e-03  -1.818   0.0905 .
## I(AsConc^2)  3.031e-04  2.043e-04   1.484   0.1601
## I(AsConc^3) -1.664e-06  1.354e-06  -1.229   0.2393
## ---
## Signif. codes:  0 '***' 0.001 '**' 0.01 '*' 0.05 '.' 0.1 ' ' 1
##
## Residual standard error: 0.07643 on 14 degrees of freedom
## Multiple R-squared:  0.5044, Adjusted R-squared:  0.3982
## F-statistic:  4.75 on 3 and 14 DF,  p-value: 0.01734
```

Plotting our models..

```r
plot(alph$AsConc, alph$Pielou, type="p", lwd=3)
points(alph$AsConc,predict(modelp1), type="l", col="green",lwd=2)
points(alph$AsConc, predict(modelp2), type="l", col="red", lwd=2)
points(alph$AsConc, predict(modelp3), type="l", col="blue", lwd=2)
```

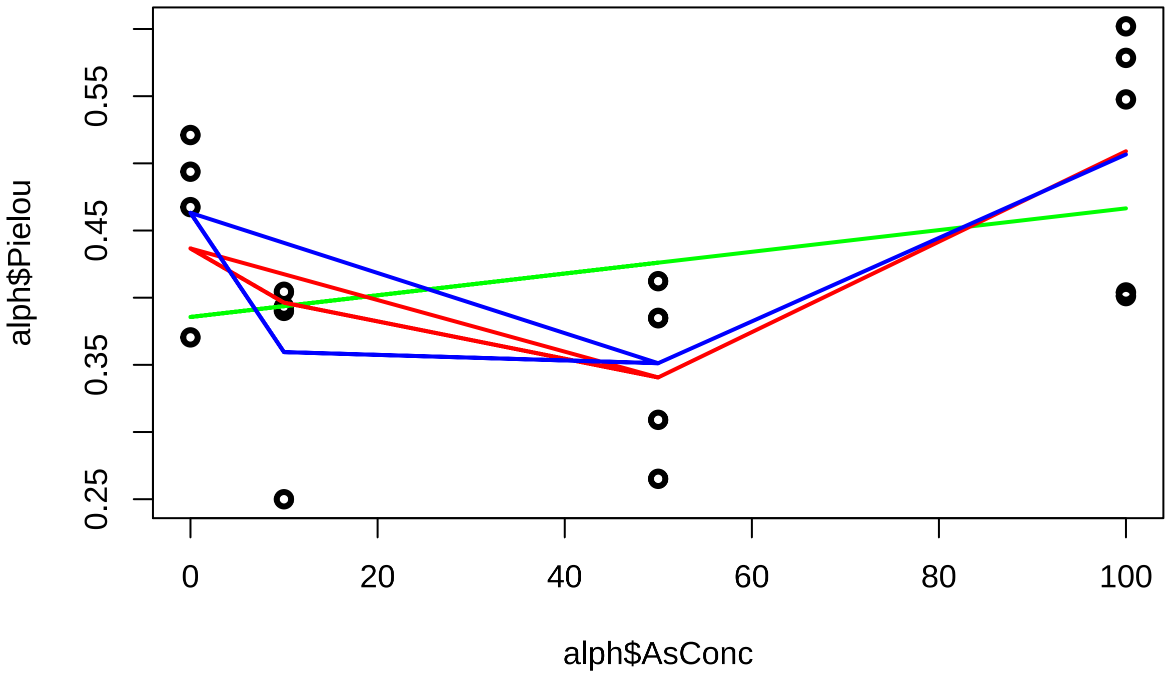

Calculating AIC for our models on Pielou's evenness..

```r
AIC(modelp1,modelp2,modelp3)
```

```
##         df       AIC
## modelp1  3 -29.51037
## modelp2  4 -36.16765
## modelp3  5 -36.01207
```

Similar to our models on Shannon diversity, we see the second model is best.

Summarize metrics in table

Then make a table of means of Observed, ACE, Shannon, Simpson and Fisher and Pielou metrics. This is for publication purposes. You can freely change the columns of diversity metrics to retain.

```r
alphmean = aggregate(alph[,c('Observed',"Shannon","Simpson","Fisher","Pielou")],list(alph$Treatment),mea
alphse = aggregate(alph[,c('Observed',"Shannon","Simpson","Fisher","Pielou")],list(alph$Treatment),se)

alphmean
```

```
##   Group.1 Observed  Shannon   Simpson   Fisher    Pielou
## 1  100ppb   392.60 3.031439 0.8221081 50.67077 0.5066237
## 2   10ppb   435.75 2.186070 0.6345519 56.98035 0.3594885
## 3   50ppb   582.40 2.223582 0.6308060 80.06221 0.3512393
## 4 Control   316.25 2.656996 0.8054348 39.52262 0.4631748
```

```r
write.csv(alphmean,"~/Documents/QIIME_fish/forward_read_analysis/bowtie_dada2_phylo/R1_nophix_split_by_
```

```r
# Multiple plot function

# ggplot objects can be passed in ..., or to plotlist (as a list of ggplot objects)
# - cols:   Number of columns in layout
# - layout: A matrix specifying the layout. If present, 'cols' is ignored.
#
# If the layout is something like matrix(c(1,2,3,3), nrow=2, byrow=TRUE),
# then plot 1 will go in the upper left, 2 will go in the upper right, and
# 3 will go all the way across the bottom.

multiplot <- function(..., plotlist=NULL, file, cols=1, layout=NULL) {
  library(grid)

  # Make a list from the ... arguments and plotlist
  plots <- c(list(...), plotlist)

  numPlots = length(plots)

  # If layout is NULL, then use 'cols' to determine layout
  if (is.null(layout)) {
    # Make the panel
    # ncol: Number of columns of plots
    # nrow: Number of rows needed, calculated from # of cols
    layout <- matrix(seq(1, cols * ceiling(numPlots/cols)),
                     ncol = cols, nrow = ceiling(numPlots/cols))
  }

 if (numPlots==1) {
    print(plots[[1]])

  } else {
    # Set up the page
    grid.newpage()
    pushViewport(viewport(layout = grid.layout(nrow(layout), ncol(layout))))

    # Make each plot, in the correct location
    for (i in 1:numPlots) {
      # Get the i,j matrix positions of the regions that contain this subplot
      matchidx <- as.data.frame(which(layout == i, arr.ind = TRUE))

      print(plots[[i]], vp = viewport(layout.pos.row = matchidx$row,
                                      layout.pos.col = matchidx$col))
    }
  }
}
```

To plot all three, take the pound sign out and run code..

```r
#multiplot(obs,shan,piel, cols = 3)
```

Beta diversity analyses

Here we will run Principle Coordinate Anlaysis (PCoA) on UniFrac distance scores to analyze beta diversity differences between communities

Load colorblind friendly palette

```
cbpaletteshort = c("#D55E00","#F0E442","#E69F00", "#0072B2")
```

Run and plot ordinations

Here I chose to use PCoA on unweighted UniFrac distance scores. There are a number of other distance metrics available, such as Jaccard, Bray Curtis and Weighted UniFrac; and other multivariate analysis available such as multidimensional scaling (NMDS) and multidimensional scaling (MDS). You can see these in the phyloseq documentaion. Also, phyloseq has a great tutorial on using the `plot_ordination` function and making scree plots.

```
#First run ordination
psFR.pc.uni <- ordinate(psF, method="PCoA", distance = "unifrac")
#Then we can see how much of total distance is captured by the eigenvalues
plot_scree(psFR.pc.uni, 'Scree plot, Unifrac/PCoA')
```

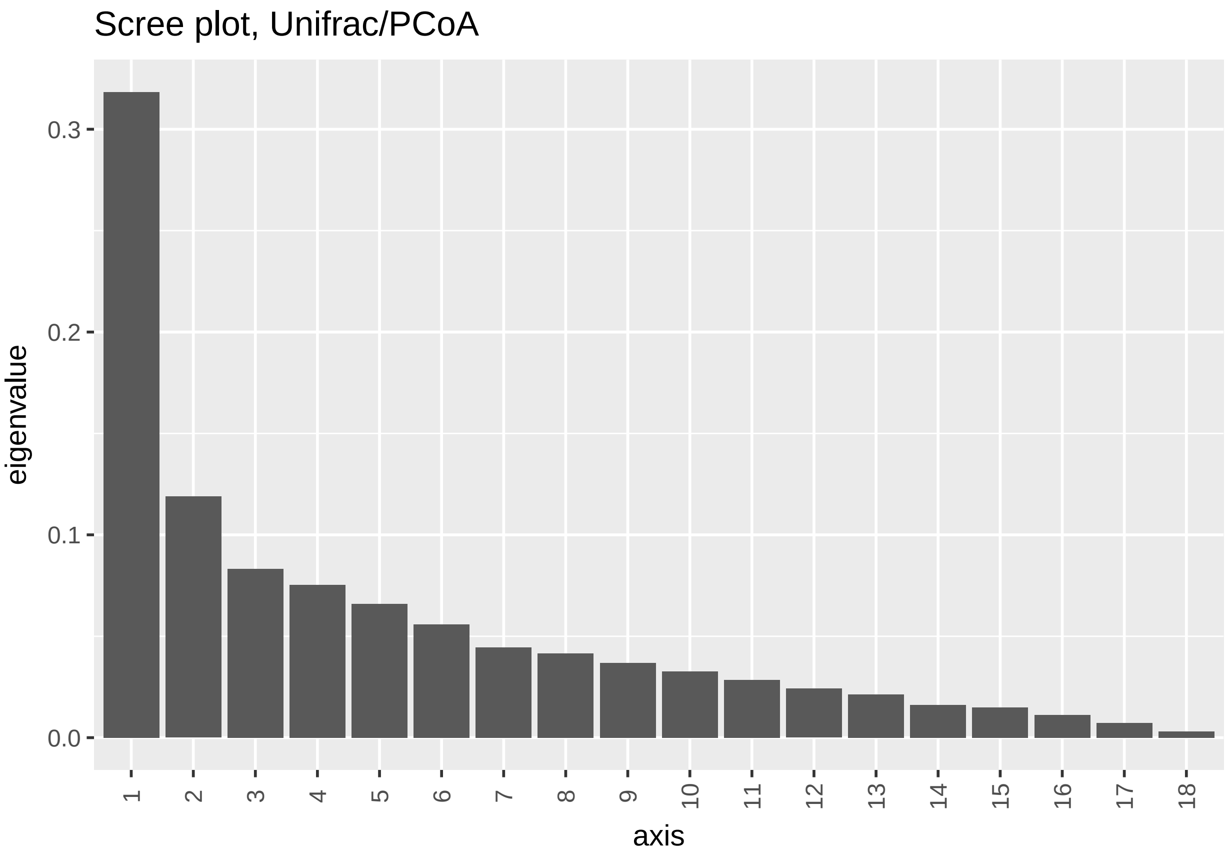

How much variation do the first two axes (ones we will plot) explain?

```
(100*sum(psFR.pc.uni$values$Relative_eig[1:2]))
```

```
## [1] 43.72482
```

Now we plot ordinations with arsenic in seperate concentrations and as a group variable.

```
#We use t-dist for ellipse b.c n < 30
p1 = plot_ordination(psF, psFR.pc.uni,color="Treatment", title="unifrac") +
  geom_point(size = 5) + scale_colour_manual(values=c("#D55E00","#F0E442", "#E69F00", "#0072B2")) + ther

p1
```

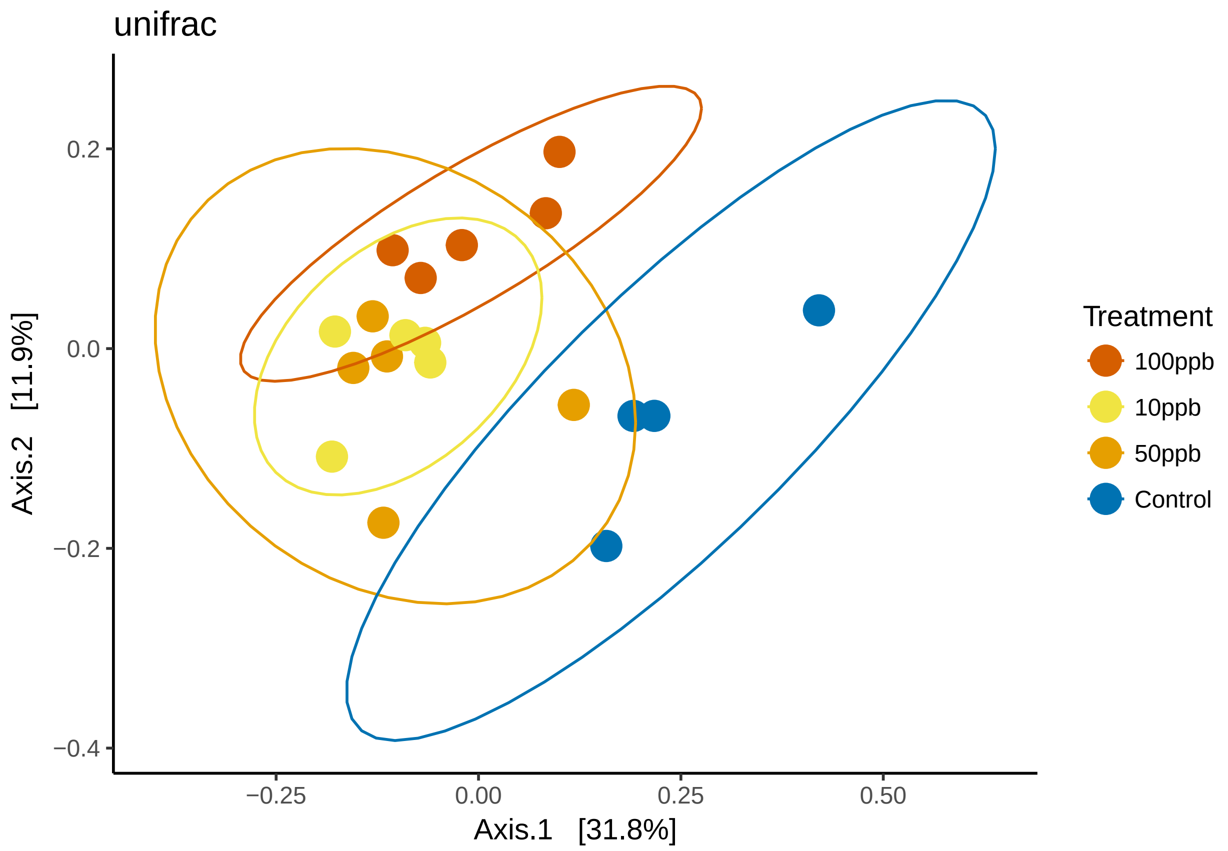


```
 p2 = plot_ordination(psF, psFR.pc.uni,color="PresAbs", title="unifrac") +
  geom_point(size = 5) +
  scale_colour_manual(values = c("#D55E00","#0072B2")) + theme_classic()  + stat_ellipse(type = "t")

p2
```

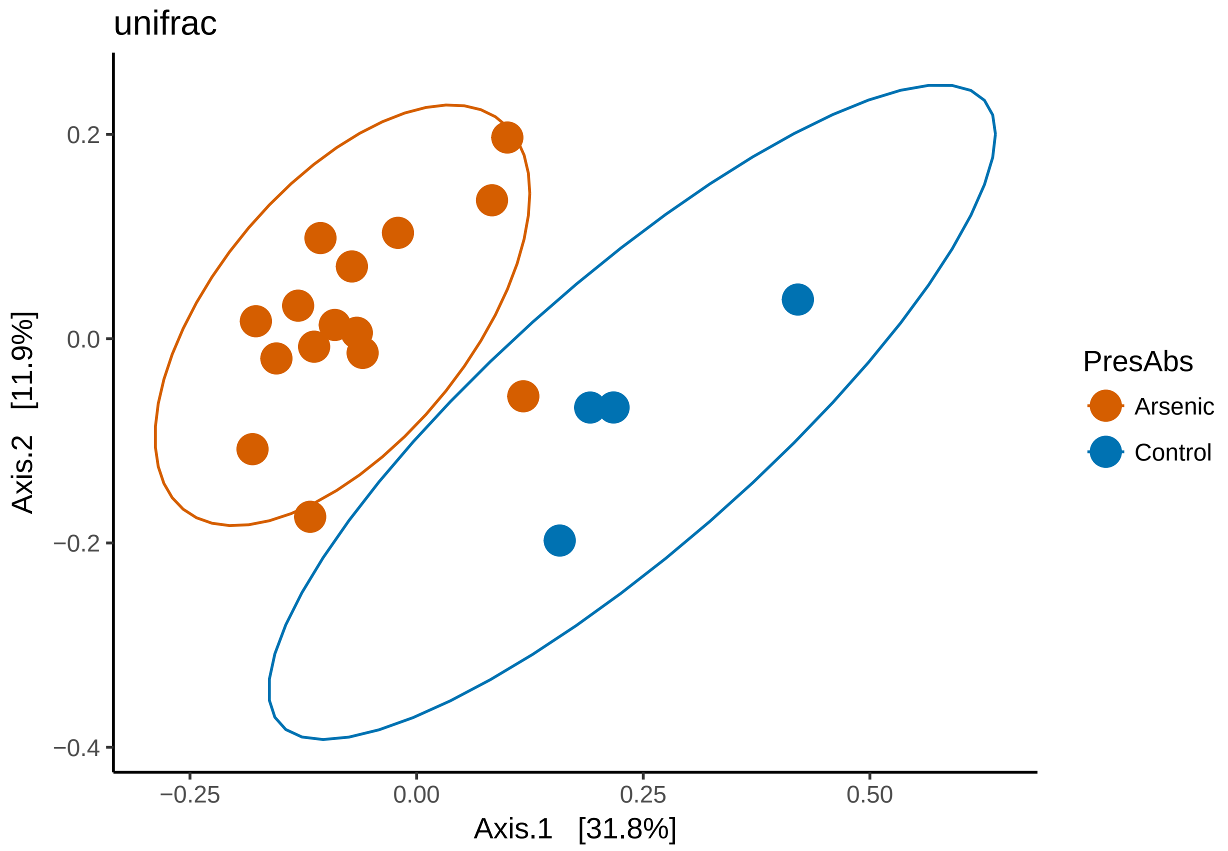


```
#Again, can plot both with multiplot
#multiplot(p1,p2,cols=2)
```

Run ANOSIM and ADONIS

We use ADONIS (see `vegan::adonis`) when we have continuous variables but have to use an ANOSIM (see `vegan::anosim`) when there are categorical variables. We are looking at the P and R2/R values. A R2/R value of 0 means that there is no dissimilarity between the groups and a value of 1.0 suggest high dissimilarity. A value <0 suggests that the within group difference is in fact greater than between group differences. If we receive significant values when conducting these tests, we want to make sure the differencs are not due to a lack of homogeneity of group dispersions, and so we verify our results using `vegan::betadisper`. Below we see our test statistics, p-values and anova tables printed.

First lets run an ADONIS..

```
#Recalculating distance and running  an adonis
df = as(sample_data(psF),"data.frame")
d=phyloseq::distance(psF,"uniFrac")
treatments_ado = adonis(d ~ sqrt(AsConc),df)
```

ADONIS table..

```
treatments_ado
```

```
##
## Call:
## adonis(formula = d ~ sqrt(AsConc), data = df)
##
```

```
## Permutation: free
## Number of permutations: 999
##
## Terms added sequentially (first to last)
##
##               Df SumsOfSqs  MeanSqs F.Model      R2 Pr(>F)
## sqrt(AsConc)  1   0.18832 0.188320  2.4204 0.12463  0.018 *
## Residuals    17   1.32270 0.077806         0.87537
## Total        18   1.51102                  1.00000
## ---
## Signif. codes:  0 '***' 0.001 '**' 0.01 '*' 0.05 '.' 0.1 ' ' 1
```

It looks like arsenic is a significant factor in predicting clustering, though modestly.

Then ANOSIM..

```
presabs = get_variable(psF, "PresAbs")
treatments_ano = anosim(phyloseq::distance(psF,"uniFrac"),presabs)
```

Significant? If so, what's the R value?

```
treatments_ano
```

```
##
## Call:
## anosim(dat = phyloseq::distance(psF, "uniFrac"), grouping = presabs)
## Dissimilarity:
##
## ANOSIM statistic R: 0.7835
##       Significance: 0.002
##
## Permutation: free
## Number of permutations: 999
```

```
summary(treatments_ano)
```

```
##
## Call:
## anosim(dat = phyloseq::distance(psF, "uniFrac"), grouping = presabs)
## Dissimilarity:
##
## ANOSIM statistic R: 0.7835
##       Significance: 0.002
##
## Permutation: free
## Number of permutations: 999
##
## Upper quantiles of permutations (null model):
##   90%   95% 97.5%   99%
## 0.236 0.315 0.380 0.492
##
## Dissimilarity ranks between and within classes:
##         0%   25%   50%    75% 100%   N
## Between 42 109.5 134.5 154.25  171  60
## Arsenic  1  28.0  55.0  90.00  157 105
## Control 22  62.5  85.5 103.25  158   6
```

The R value above is telling us how dissimilar they are. Between 0 and 1. 0 meaning not diff and 1 meaning

very diff. Ours is 0.78.

```
#Use betadisper to to test for homogeneity of groups dispersions on both of these...
#Continous As conc first
zu_45 <- as(sample_data(psF), "data.frame")
groups <- zu_45[["AsConc"]]
mod<-betadisper(d,groups)
a = anova(mod)
a
```

```
## Analysis of Variance Table
##
## Response: Distances
##           Df   Sum Sq   Mean Sq F value Pr(>F)
## Groups     3 0.004696 0.0015654  0.5082 0.6825
## Residuals 15 0.046203 0.0030802
```

```
#Categorical second
groups2 <- zu_45[["PresAbs"]]
mod2<-betadisper(d,groups2)
a2 = anova(mod2)
a2
```

```
## Analysis of Variance Table
##
## Response: Distances
##           Df   Sum Sq   Mean Sq F value Pr(>F)
## Groups     1 0.000073 0.0000734  0.0237 0.8796
## Residuals 17 0.052751 0.0031030
```

```
par(mfrow = c(2,2))
plot(mod)
boxplot(mod)
plot(mod2)
boxplot(mod2)
```

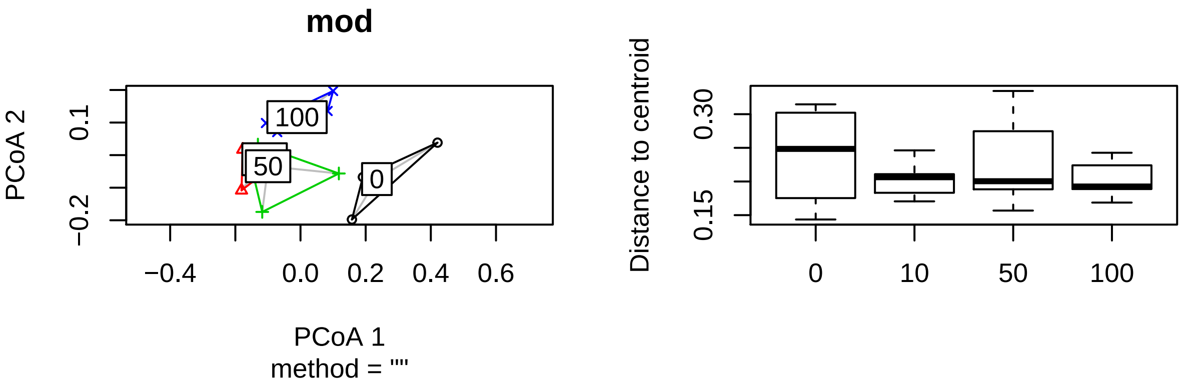

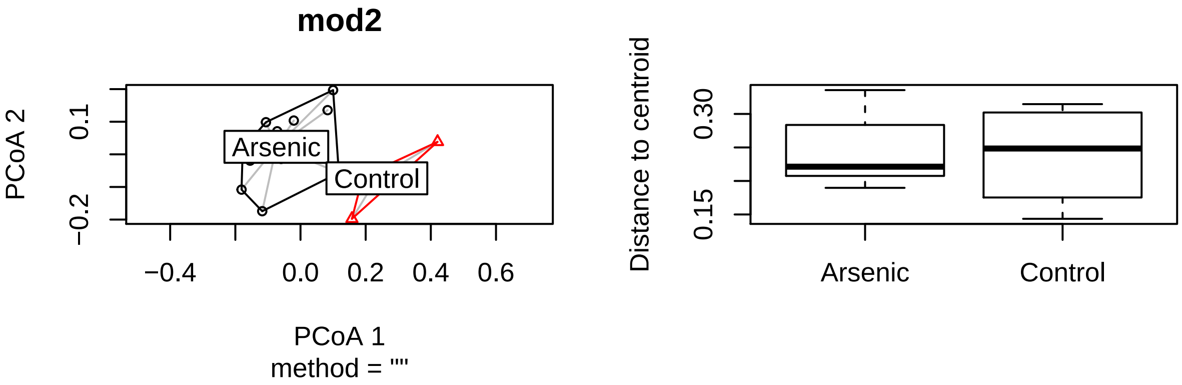


```
#Adjust the p-values for multiple stats run on the distance matrix
p.adjust(c(treatments_ado$aov.tab$`Pr(>F)`[1],a$`Pr(>F)`[1],treatments_ano$signif, a2$`Pr(>F)`[1]), met
```

```
## [1] 0.0360000 0.8795768 0.0080000 0.8795768
```

Biplot

We can plot taxa alongside our samples and labeled treatments to try and make observations as to how these taxa might be driving differences in sample composition. The threshold to plot is rather arbitrary, but we want to plot few enough genera so that we can see labels and make inferences about the genera that are within treatment clusters.

First let's plot all the taxa in our minimally filtered table to see large-scale patterns..

```
p3 = plot_ordination(psF,psFR.pc.uni, type="biplot",color  ="Phylum",title="biplot",shape = "PresAbs",la
```

```
## Warning: Ignoring unknown aesthetics: na.rm
```

```
p3 + theme_classic()
```

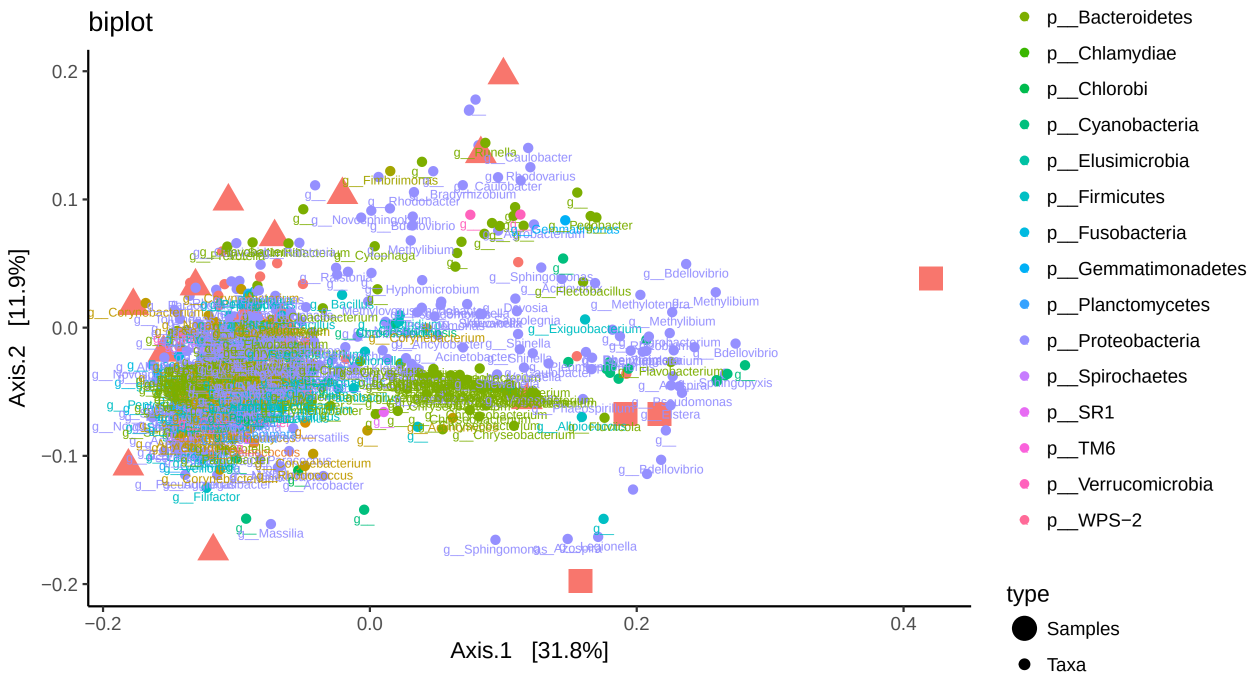

This is actually pretty interesting. It shows many more genera around the 10ppb and 50ppb treatments. This could very well be related to the increased observed species and decreased Pielou's evenness that we see in these samples compared to the control and 100ppb.

However to make inferences as to how some of the more abundant species might be driving differences in communities, we will use our filtered table that only contains the 15 most abundant families.

```
p4 = plot_ordination(psFRFam,psFR.pc.uni, type="biplot",color  ="Phylum",title="biplot",shape = "PresAb
```

```
## Warning: Ignoring unknown aesthetics: na.rm
```

```
p4 + theme_classic()
```

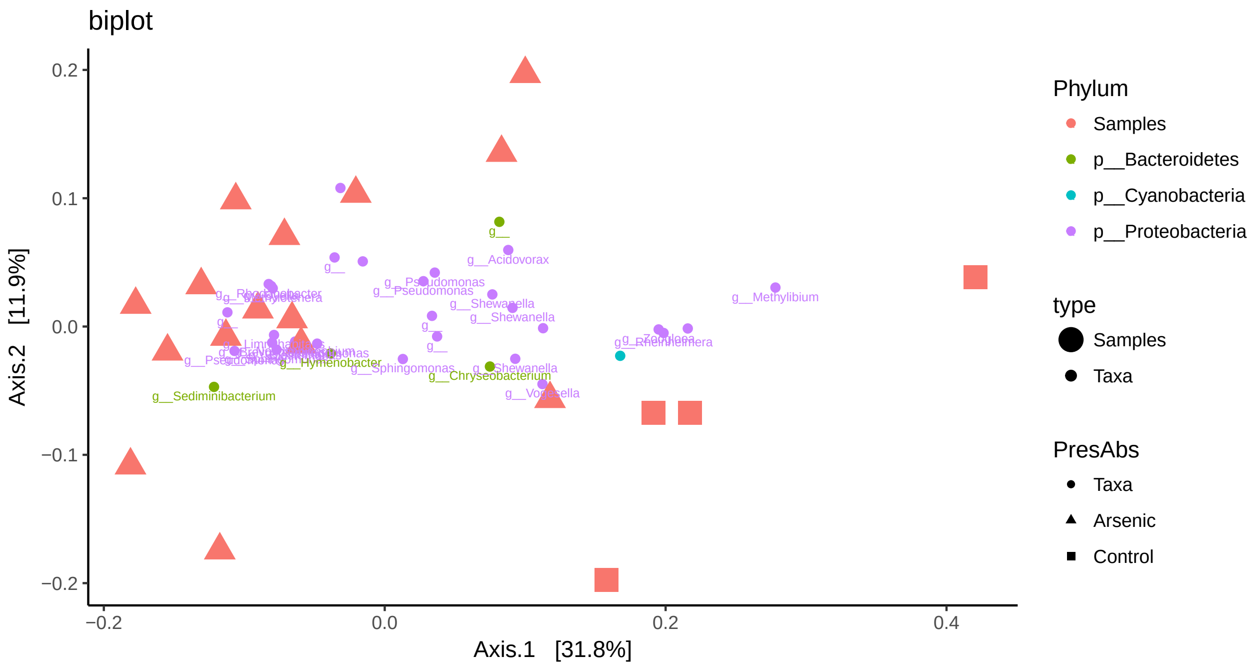

Here we see that genera such as Sediminibacterium and Methylotenera cluster with the arsenic samples. This is just observational and we now go on to the DESeq2 analysis to make more statistically motivated inferences about differentially abundant genera.

DESeq2 differential abundance

The methods in this script are borrowed from a couple of different places. In short, I combine some methods from the phyloseq extension with parts from QIIME's open source DESeq2 script. Uniquely, QIIMErs suggest to add 1 to the feature abundance data, which allows for a better fit with a local model.

Differential abundance analysis and plotting dispersion estimates

There are three fitTypes available (local, paramteric and mean). After testing all three, I found the local fit was best. Though this is data dependent, I haven't seen anything other than a local fit used. De_factor is our metadata category that has all the samples organized based on presence and absence of arsenic.

```
#Make ISeV table a data frame
psdt<-as.data.frame(otu_table(pspy))

colnames <- sample_data(pspy)

#Transpose the ISeV table.
psdtt<-t(psdt)
#Make a matrix
psdtt = as(psdtt,"matrix")
#Add 1 to all values
psdtt = psdtt + 1

#Make a DESeq dataset from our matrix
dds<- DESeqDataSetFromMatrix(psdtt,colnames,design=as.formula(paste("~","De_factor")))
```

```
## Warning in class(from) <- NULL: Setting class(x) to NULL; result will no
## longer be an S4 object

## converting counts to integer mode
```

```
#Run the DESeq2's main function - a differential expression analysis based on the Negative Binomial dis
dds<- DESeq(dds, fitType = "local", quiet = TRUE)
plotDispEsts(dds)
```

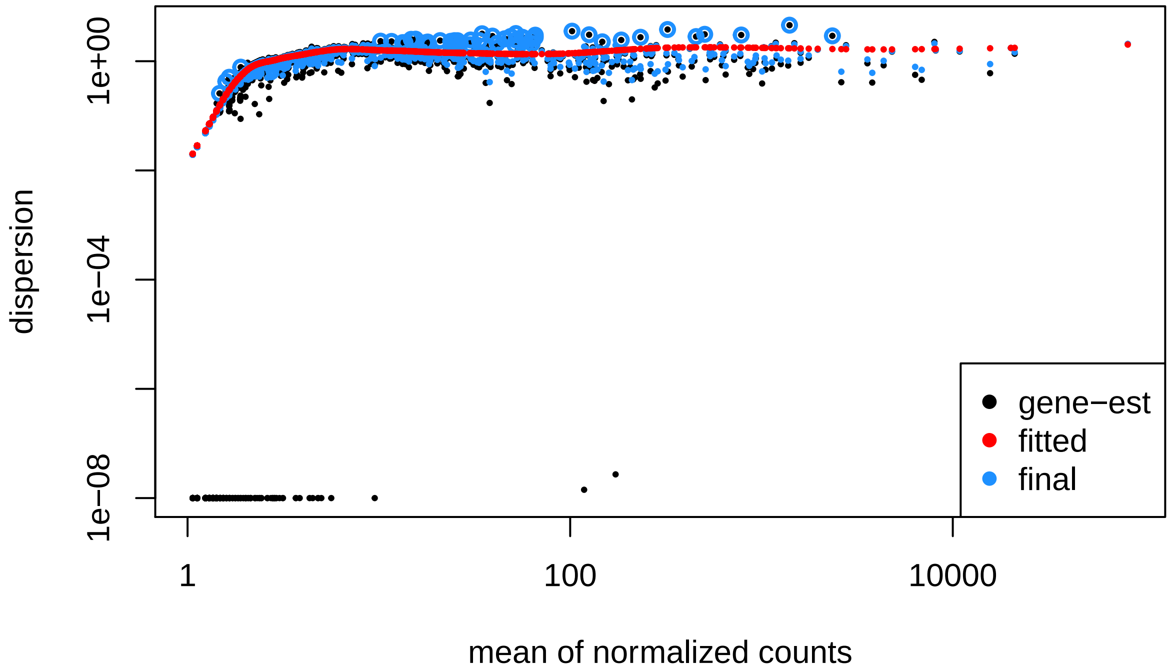

Extract differential abundance results

Here we compare the experimental group to the control group. The order is important! We put the experimental first and control second, allowing us to eventually see the log2fold change in bacterial abundance in the presence of arsenic. We then write this out to a table. We can do this where P<0.05 and P<=0.01.

```
res <- results(dds, contrast = c("De_factor", "present", "absent"))
res = res[order(res$padj, na.last=NA), ]
sigtab05 = res[(res$padj < 0.05), ]

dim(sigtab05)
```

```
## [1] 186   6
```

```
#Export this to use as a table (for supplementary)
sigtab05 = cbind(as(sigtab05, "data.frame"), as(tax_table(pspy)[rownames(sigtab05), ], "matrix"))
write.csv(sigtab05,"~/Documents/QIIME_fish/forward_read_analysis/bowtie_dada2_phylo/R1_nophix_split_by_

#For some reason there are no genus classification in some spots and they just read g__ followed by bla

#First, asting the factors to characters so they can be replaced..
sigtab05$Genus <-as.character(sigtab05$Genus)
#Replacing the values with NA
sigtab05$Genus <- replace(sigtab05$Genus, sigtab05$Genus=="g__", NA)



#For more stringent significance parameters (i.e. p <= 0.01)

#Subset results where p <= 0.01
sigtab01 = res[(res$padj <= 0.01), ]
```

```r
dim(sigtab01)
```

```
## [1] 79  6
```

```r
#Bind as a dataframe with taxa names
sigtab01 = cbind(as(sigtab01, "data.frame"), as(tax_table(pspy)[rownames(sigtab01), ], "matrix"))

#Write out as csv
write.csv(sigtab01,"~/Documents/QIIME_fish/forward_read_analysis/bowtie_dada2_phylo/R1_nophix_split_by_s

#Same as above for 0.05 - change g__
sigtab01$Genus <-as.character(sigtab01$Genus)
sigtab01$Genus <- replace(sigtab01$Genus, sigtab01$Genus=="g__", NA)
sigtabna01 = subset(sigtab01, !is.na(Genus))
```

We can also make tables to show us how many of each genera increase and decrease in abundance at the 0.01 significant levels. This is easily switched to 0.05 by changing threshold below. We can use this to compare to those genera that change in abundance using an OTU table generated via the QIIME pipeline.

Same thing at different significance level.

```r
#Subset those that increased in abundance and where p<=0
possigtab01 = sigtabna01[(sigtabna01$log2FoldChange >=0) & (sigtabna01$padj <= 0.01),]
#Subset those that decreased in abundance and where p<=0
negsigtab01 = sigtabna01[(sigtabna01$log2FoldChange <=0) & (sigtabna01$padj <= 0.01),]

#Make tables
posc01 <- as.data.frame(table(possigtab01$Genus))
negc01 <- as.data.frame(table(negsigtab01$Genus))

#Add increase and decrease labels
posc01[["change"]] <- (rep(c("Increase"),dim(posc01)[1]))
negc01[["change"]] <- (rep(c("Decrease"),dim(negc01)[1]))

#Bind the two dataframes
posnegc01 = rbind(posc01,negc01)
#Write out
write.csv(posnegc01, "~/Documents/QIIME_fish/forward_read_analysis/bowtie_dada2_phylo/R1_nophix_split_by
posnegc01
```

```
##                     Var1 Freq   change
## 1      g__Acinetobacter    2 Increase
## 2        g__Actinomyces    1 Increase
## 3        g__Alcanivorax    1 Increase
## 4        g__Dyadobacter    1 Increase
## 5  g__Janthinobacterium    2 Increase
## 6            g__Kaistia    1 Increase
## 7    g__Methylobacillus    1 Increase
## 8      g__Methylotenera    1 Increase
## 9         g__Rhodoferax    1 Increase
## 10            g__Rothia    1 Increase
## 11 g__Sediminibacterium   10 Increase
## 12     g__Agrobacterium    1 Decrease
## 13         g__Azospira    1 Decrease
## 14      g__Bdellovibrio    1 Decrease
## 15       g__Caulobacter    1 Decrease
```

```
## 16           g__Elstera    1 Decrease
## 17    g__Flavobacterium    1 Decrease
## 18        g__Legionella    1 Decrease
## 19         g__Mycoplana    1 Decrease
## 20       g__Pseudomonas    1 Decrease
## 21         g__Rhizobium    1 Decrease
## 22      g__Sphingopyxis    1 Decrease
## 23          g__Zoogloea    1 Decrease
```

Plot DESeq2 results

We choose to color and fill by Phylum and label by Genus.

```
#Define our discrete palette
#scale_fill_discrete <- function(palname = "Set1", ...) {
#scale_fill_brewer(palette = palname, ...)
#}

#Sort our table
x = tapply(sigtab05$log2FoldChange, sigtab05$Phylum, function(x) max(x))
x = sort(x, TRUE)
#Cast as factors
sigtab05$Phylum = factor(as.character(sigtab05$Phylum), levels=names(x))
#Same for genus
x = tapply(sigtab05$log2FoldChange, sigtab05$Genus, function(x) max(x))
x = sort(x, TRUE)
sigtab05$Genus = factor(as.character(sigtab05$Genus), levels=names(x))

#Count how many NAs increase and decrease in abundance, make a note of it and them omit them for plotti
sigtab05pos = sigtab05[(sigtab05$log2FoldChange >=0),]
pos.row.has.na <- apply(sigtab05pos, 1, function(x){any(is.na(x))})
sum(pos.row.has.na)
```

```
## [1] 46
```

We see there were 43 NAs that increased in abundance

```
sigtab05neg = sigtab05[(sigtab05$log2FoldChange <=0),]
neg.row.has.na <- apply(sigtab05neg, 1, function(x){any(is.na(x))})
sum(neg.row.has.na)
```

```
## [1] 37
```

We see there were 37 NAs that decreased in abundance.

```
#Filter these NAs fro mthe table we will use for plotting..
row.has.na <- apply(sigtab05, 1, function(x){any(is.na(x))})
sigtab05.filtered <- sigtab05[!row.has.na,]



#Plot
de = ggplot(sigtab05.filtered, aes(x=Genus, y  =log2FoldChange, color=Phylum, fill=Phylum)) + geom_poin
  theme(axis.text.x = element_text(angle = -90, hjust = 0, vjust=0.5),
        panel.grid.minor = element_blank()) +
  ylab("log2FoldChange")
```

```r
de + scale_fill_hue(l=80,c=150) + ylim(-6,6)
```

```
## Warning: Removed 3 rows containing missing values (geom_point).
```

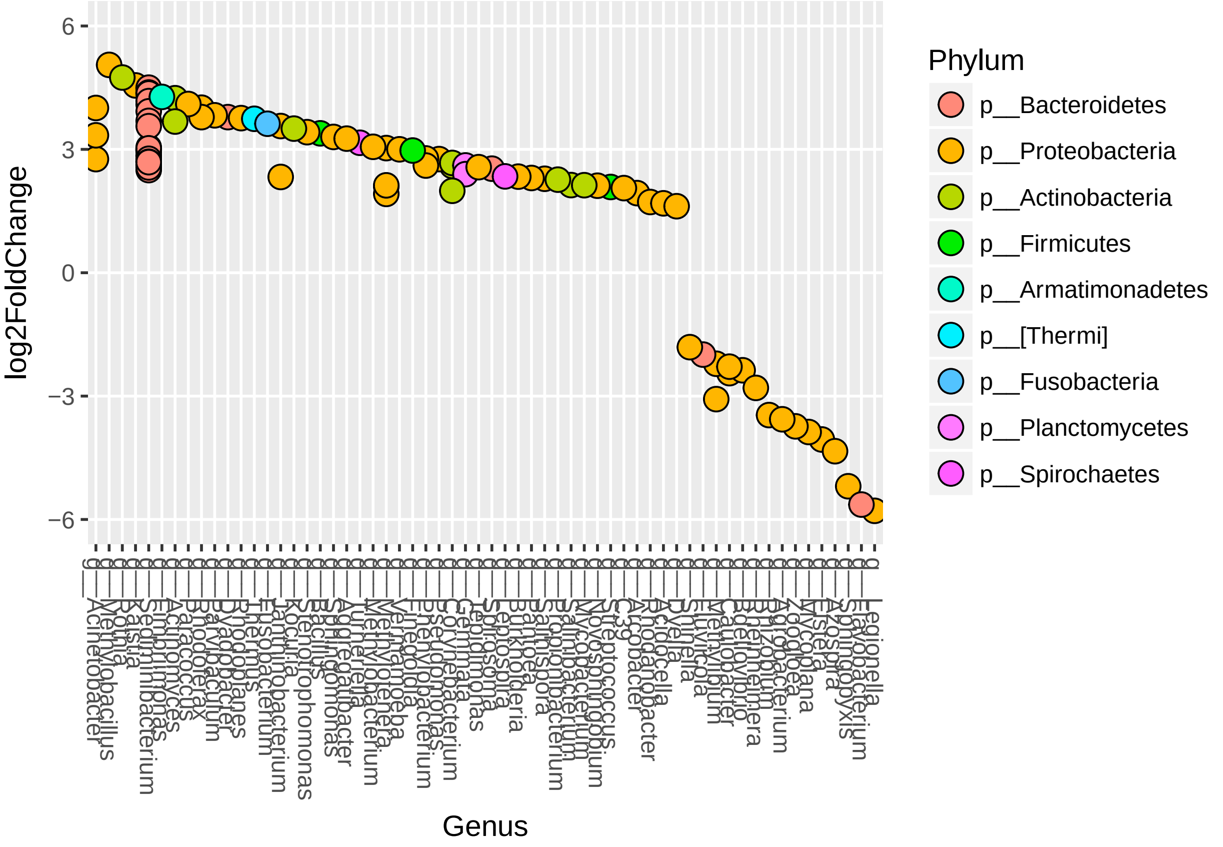

For turning 90 and making a vertical fig in Illustrator, you can turn this figure by changing lims to (6,-6)

Then we can apply the same functions and make a figure for where p<=0.01..

```r
#Time for 0.01 figure..
x = tapply(sigtab01$log2FoldChange, sigtab01$Phylum, function(x) max(x))
x = sort(x, TRUE)
sigtab01$Phylum = factor(as.character(sigtab01$Phylum), levels=names(x))
x = tapply(sigtab01$log2FoldChange, sigtab01$Genus, function(x) max(x))
x = sort(x, TRUE)
sigtab01$Genus = factor(as.character(sigtab01$Genus), levels=names(x))

de01 = ggplot(sigtab01, aes(x=Genus, y  =log2FoldChange, color=Phylum, fill=Phylum)) + geom_point(colou
  theme(axis.text.x = element_text(angle = -90, hjust = 0, vjust=0.5),
        panel.grid.minor = element_blank()) +
  ylab("log2FoldChange")

#With colorblind friendly palette
de01 + scale_fill_manual(values=c("#E69F00", "#56B4E9", "#009E73", "#F0E442", "#0072B2", "#D55E00", "#C
```

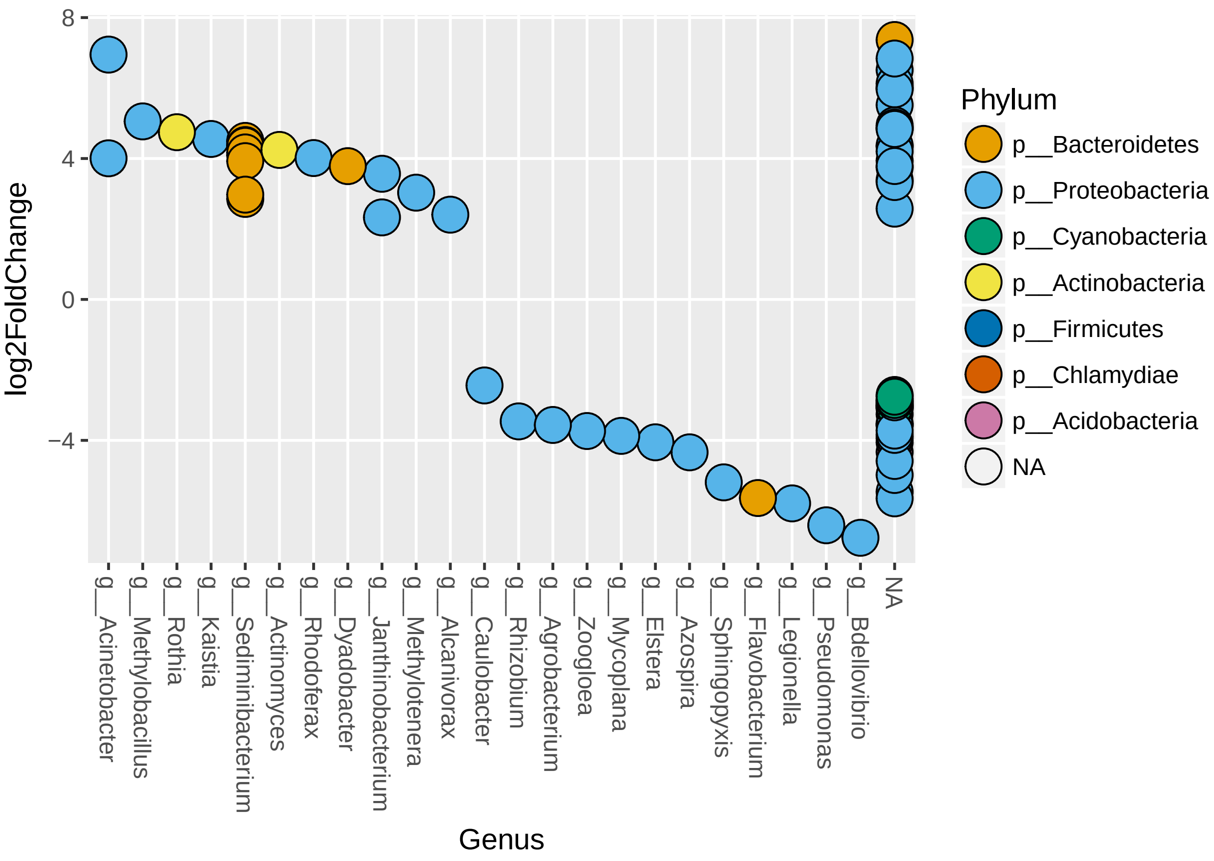

phyloseq/DESeq2/vegan analysis on DADA2 processed ISeVs ends here

---

Alternative analysis on QIIME processed OTU table (clustered w/ USEARCH)

To learn more about how to prepare these files and QIIME's defaults, check the QIIME script index and QIIME Illumina tutorial. Also, before importing them to phyloseq you need to convert QIIME's biom files to json format with the `biom convert` script.

For example…

`biom convert -i otu_table_mc2_w_tax.biom --to-json --table-type "OTU table" -o otu_table_mc2_w_tax_json`

Import OTU table, tree and metadata.

```
#Path to OTU table from QIIME - converted to JSON format
#Make sure biom file is in json format
jsonbiomfile = "~/Documents/QIIME_fish/forward_read_analysis/bowtie_dada2_phylo/dada2_usearch/qiime_anal

#OR... for benchmarking
jsonbiomfileallOTUs = "~/Documents/QIIME_fish/forward_read_analysis/bowtie_dada2_phylo/dada2_usearch/qi

treefilename = '~/Documents/QIIME_fish/forward_read_analysis/bowtie_dada2_phylo/dada2_usearch/qiime_anal

#Make phloseq object with biom file
```

```r
otups = import_biom(jsonbiomfile, treefilename, parseFunction = parse_taxonomy_default)


#Change rank names from Rank1, Rank2 etc.. to Kingdom, Phylum etc..
colnames(tax_table(otups)) <- c(Rank1 = "Kingdom", Rank2 = "Phylum", Rank3 = "Class", Rank4 = "Order", 


#Merge metadata with phyloseq object. Again, you can do this for all of the phyloseq objects.
otupsmd = merge_phyloseq(metad,otups)

otupsmd
```

```
## phyloseq-class experiment-level object
## otu_table()   OTU Table:         [ 8263 taxa and 20 samples ]
## tax_table()   Taxonomy Table:    [ 8263 taxa by 7 taxonomic ranks ]
## phy_tree()    Phylogenetic Tree: [ 8263 tips and 8261 internal nodes ]
```

Looks good!

Let's see sample sums..

```r
#Sample sums
sample_sums(otupsmd)
```

```
##  DahanA6  DahanA7 DahanA12  DahanB1  DahanB7  DahanA8  DahanA9  DahanB5
##   252699   324543   284752   270687   101902   222903   244352   132074
##  DahanA1  DahanB4  DahanB6  DahanB8 DahanA11  DahanA4 DahanA10  DahanA5
##   321148    96879   175150   236016   241687   243167   235135   313676
##  DahanB2  DahanA3  DahanB3  DahanA2
##   167151   285118   233293        4
```

```r
#Lets not forget to remove DahanA2
otupsmd <- prune_samples(sample_sums(otupsmd)>=20, otupsmd)
```

How many taxa were there in the table before PyNAST filtering?

```r
otupsnopy = import_biom(jsonbiomfileallOTUs, parseFunction = parse_taxonomy_default)

otupsnopy
```

```
## phyloseq-class experiment-level object
## otu_table()   OTU Table:         [ 20015 taxa and 20 samples ]
## tax_table()   Taxonomy Table:    [ 20015 taxa by 7 taxonomic ranks ]
```

As we might expect we have the most taxa with our raw, unfiltered OTU table (20015) and fewer with our PyNAST aligned table (8263). We used the table that included PyNAST failed sequences object to compare raw OTU v.s. ISeV counts post the initial read processing and the one without PyNAST failed sequences for community analyses. Likewise, our DADA2 reads retained all sequences for the initial comparison but not for benchmarking the two methods. To be clear - use the `otupsmd` object for community analyses. **So that we really limit our comparison between the OTU and ISeV table with respects to uclust v. DADA2, treat the filtering and processing, once in phyloseq, the same!**
