# Supplementary material for: Exposure to Arsenic Alters the Microbiome of Larval Zebrafish
Source: Front Microbiol. 2018 Jun 21;9:1323. doi: 10.3389/fmicb.2018.01323 (PMC6021535; doi:10.3389/fmicb.2018.01323)
Supplement: Tables S4, S5 — ANOVA tables for PCoA comparisons. [file Table_4.DOC]

**Table S4. ANOVA table for effect of treatments on sample distance to centroid in PCoA**

| ***Response: treatment distances*** | ***Df*** | ***SS*** | ***MS*** | ***F*** | ***Adj-P*** |
| --- | --- | --- | --- | --- | --- |
| Groups | 3 | 0.004696 | 0.0015654 | 0.5082 | 0.8796 |
| Residuals | 15 | 0.046203 | 0.0030802 |  |  |
