# Supplementary material for: Exposure to Arsenic Alters the Microbiome of Larval Zebrafish
Source: Front Microbiol. 2018 Jun 21;9:1323. doi: 10.3389/fmicb.2018.01323 (PMC6021535; doi:10.3389/fmicb.2018.01323)
Supplement: Supplementary file 15 [file Table_5.DOC]

**Table S5. ANOVA table for effect of pooled arsenic treatment vs control group on sample distance to centroid in PCoA**

| ***Response: group distances*** | ***Df*** | ***SS*** | ***MS*** | ***F*** | ***Adj-P*** |
| --- | --- | --- | --- | --- | --- |
| Groups | 1 | 0.000073 | 0.0000734 | 0.0237 | 0.8796 |
| Residuals | 17 | 0.052751 | 0.003103 |  |  |
