# Supplementary material for: Exposure to Arsenic Alters the Microbiome of Larval Zebrafish
Source: Front Microbiol. 2018 Jun 21;9:1323. doi: 10.3389/fmicb.2018.01323 (PMC6021535; doi:10.3389/fmicb.2018.01323)
Supplement: Table S7 — Genera counts of differentially abundant ASVs and OTUs. [file Table_7.DOC]

**Table S7. Genera counts of differentially abundant RSVs and OTUs.**

| ***Genus*** | ***RSV Increase*** | ***OTU increase*** | ***RSV decrease*** | ***OTU decrease*** |
| --- | --- | --- | --- | --- |
| *Zoogloea* |  |  | 1 | 10 |
| *Sphingopyxis* |  |  | 1 |  |
| *Rhizobium* |  |  | 1 |  |
| *Pseudomonas* |  |  | 1 | 7 |
| *Mycoplana* |  |  | 1 |  |
| *Elstera* |  |  | 1 |  |
| *Caulobacter* |  |  | 1 |  |
| *Bdellovibrio* |  |  | 1 | 2 |
| *Azospira* |  |  | 1 |  |
| *Agrobacterium* |  |  | 1 | 7 |
| *Sediminibacterium* | 10 |  |  |  |
| *Janthinobacterium* | 2 | 2 |  |  |
| *Acinetobacter* | 2 | 2 |  |  |
| *Turneriella* | 1 |  |  |  |
| *Rothia* | 1 |  |  |  |
| *Rhodoplanes* | 1 |  |  |  |
| *Rhodoferax* | 1 |  |  |  |
| *Parvibaculum* | 1 |  |  |  |
| *Methylotenera* | 1 |  |  |  |
| *Methylobacillus* | 1 |  |  |  |
| *Kaistia* | 1 |  |  |  |
| *Dyadobacter* | 1 |  |  |  |
| *Alcanivorax* | 1 | 1 |  |  |
| *Actinomyces* | 1 |  |  |  |
| *Streptococcus* |  | 1 |  |  |
| *Shewanella* |  |  |  | 1 |
| *Salinispora* |  | 1 |  |  |
| *Salinibacterium* |  | 1 |  |  |
| *Rheinheimera* |  |  |  | 8 |
| *Ramlibacter* |  |  |  | 1 |
| *Phenylobacterium* |  | 1 |  | 2 |
| *Legionella* |  |  |  | 1 |
| *Kaistia* |  | 1 |  |  |
| *Exiguobacterium* |  |  |  | 1 |
| *Dechloromonas* |  |  |  | 1 |
| *Corynebacterium* |  | 2 |  |  |
| *C39* |  | 1 |  |  |
| *Acidovorax* |  |  |  | 1 |
