# Supplementary material for: Exposure to Arsenic Alters the Microbiome of Larval Zebrafish
Source: Front Microbiol. 2018 Jun 21;9:1323. doi: 10.3389/fmicb.2018.01323 (PMC6021535; doi:10.3389/fmicb.2018.01323)
Supplement: Tables S8–S10 — Polynomial regressions for effect of arsenic on OTU alpha diversity. [file Table_8.DOC]

**Table S8. Polynomial regression summary output from modeling arsenic as predictor on observed OTUs.** Community richness based on OTU table.

| ***Coefficients:*** | ***Estimate*** | ***SE*** | | ***T*** | | ***P*** | |
| --- | --- | --- | --- | --- | --- | --- | --- |
| (Intercept) | 7.03E+00 | 7.11E-02 | | 98.9 | | <2e-16 | |
| Arsenic conc. | 7.92E-03 | 4.18E-03 | | 1.896 | | 0.0761 | |
| Arsenic conc.^2 | -6.52E-05 | 3.98E-05 | | -1.637 | | 0.121 | |
| ***Residual standard error:*** | 0.178;16 dfs | ***F*_(2,15)_*:*** | 2.12 | | | |  |
| ***Multiple R-squared:*** | 0.209 | ***P:*** | 0.153 | |  | |  |
| ***Adjust R-squared:*** | 0.111 |  | |  | |  | |
