# Supplementary material for: Exposure to Arsenic Alters the Microbiome of Larval Zebrafish
Source: Front Microbiol. 2018 Jun 21;9:1323. doi: 10.3389/fmicb.2018.01323 (PMC6021535; doi:10.3389/fmicb.2018.01323)
Supplement: Supplementary file 19 [file Table_9.DOC]

**Table S9. Polynomial regression summary output from modeling arsenic as predictor on Shannon diversity of OTUs.** Community richness and evenness based on OTU table.

| ***Coefficients:*** | ***Estimate*** | | ***SE*** | | ***T*** | ***P*** |
| --- | --- | --- | --- | --- | --- | --- |
| (Intercept) | 3.26E+00 | | 1.57E-01 | | 20.7 | 5.43E-13 |
| Arsenic conc. | 9.16E-03 | | 9.24E-03 | | 0.991 | 0.336 |
| Arsenic conc.^2 | -4.27E-05 | | 8.81E-05 | | -0.485 | 0.635 |
| ***Residual standard error:*** | | 0.394; 16 dfs | | ***F*_(2,15)_*:*** | 2.31 | |
| ***Multiple R-squared:*** | | 0.224 | | ***P:*** | 0.131 | |
| ***Adjust R-squared:*** | | 0.127 | |  |  | |
