# Supplementary material for: Exposure to Arsenic Alters the Microbiome of Larval Zebrafish
Source: Front Microbiol. 2018 Jun 21;9:1323. doi: 10.3389/fmicb.2018.01323 (PMC6021535; doi:10.3389/fmicb.2018.01323)
Supplement: Supplementary file 20 [file Table_10.DOC]

**Table S10. Polynomial regression summary output from modeling arsenic as predictor on Pielou’s evenness of OTUs.** Community evenness based on OTU table.

| ***Coefficients:*** | ***Estimate*** | | ***SE*** | | ***T*** | | | ***P*** | | |  |
| --- | --- | --- | --- | --- | --- | --- | --- | --- | --- | --- | --- |
| (Intercept) | 4.65E-01 | | 2.04E-02 | | 22.8 | | | 1.23E-13 | | |  |
| Arsenic conc. | 7.36E-04 | | 1.20E-03 | | 0.615 | | | 0.547 | | |  |
| Arsenic conc.^2 | -1.51E-06 | | 1.14E-05 | | -0.133 | | | 0.896 | | |  |
| ***Residual standard error:*** | | 0.051; 16 dfs | | ***F*_(2,15)_*:*** | | 1.91 | | |  |  |  |
| ***Multiple R-squared:*** | | 0.193 | | ***P:*** | | 0.180 | | | |  |  |
| ***Adjust R-squared:*** | | 0.092 | |  | | |  | | | | |
